# Genomic Analysis of the Hydrocarbon-Producing, Cellulolytic, Endophytic Fungus *Ascocoryne sarcoides*

**DOI:** 10.1371/journal.pgen.1002558

**Published:** 2012-03-01

**Authors:** Tara A. Gianoulis, Meghan A. Griffin, Daniel J. Spakowicz, Brian F. Dunican, Cambria J. Alpha, Andrea Sboner, A. Michael Sismour, Chinnappa Kodira, Michael Egholm, George M. Church, Mark B. Gerstein, Scott A. Strobel

**Affiliations:** 1 Department of Genetics, Harvard Medical School, Boston, Massachusetts, United States of America; 2 Wyss Institute for Biologically Inspired Engineering, Boston, Massachusetts, United States of America; 3 Program in Computational Biology and Bioinformatics, Yale University, New Haven, Connecticut, United States of America; 4 Department of Molecular Biophysics and Biochemistry, Yale University, New Haven, Connecticut, United States of America; 5 Roche 454 Life Sciences, Branford, Connecticut, United States of America; 6 Pall Corporation, Long Island City, New York, United States of America; Université du Littoral Cote d'Opale, France

## Abstract

The microbial conversion of solid cellulosic biomass to liquid biofuels may provide a renewable energy source for transportation fuels. Endophytes represent a promising group of organisms, as they are a mostly untapped reservoir of metabolic diversity. They are often able to degrade cellulose, and they can produce an extraordinary diversity of metabolites. The filamentous fungal endophyte *Ascocoryne sarcoides* was shown to produce potential-biofuel metabolites when grown on a cellulose-based medium; however, the genetic pathways needed for this production are unknown and the lack of genetic tools makes traditional reverse genetics difficult. We present the genomic characterization of *A. sarcoides* and use transcriptomic and metabolomic data to describe the genes involved in cellulose degradation and to provide hypotheses for the biofuel production pathways. In total, almost 80 biosynthetic clusters were identified, including several previously found only in plants. Additionally, many transcriptionally active regions outside of genes showed condition-specific expression, offering more evidence for the role of long non-coding RNA in gene regulation. This is one of the highest quality fungal genomes and, to our knowledge, the only thoroughly annotated and transcriptionally profiled fungal endophyte genome currently available. The analyses and datasets contribute to the study of cellulose degradation and biofuel production and provide the genomic foundation for the study of a model endophyte system.

## Author Summary

A renewable source of energy is a pressing global need. The biological conversion of lignocellulose to biofuels by microorganisms presents a promising avenue, but few organisms have been studied thoroughly enough to develop the genetic tools necessary for rigorous experimentation. The filamentous-fungal endophyte *A. sarcoides* produces metabolites when grown on a cellulose-based medium that include eight-carbon volatile organic compounds, which are potential biofuel targets. Here we use broadly applicable methods including genomics, transcriptomics, and metabolomics to explore the biofuel production of *A. sarcoides*. These data were used to assemble the genome into 16 scaffolds, to thoroughly annotate the cellulose-degradation machinery, and to make predictions for the production pathway for the eight-carbon volatiles. Extremely high expression of the gene swollenin when grown on cellulose highlights the importance of accessory proteins in addition to the enzymes that catalyze the breakdown of the polymers. Correlation of the production of the eight-carbon biofuel-like metabolites with the expression of lipoxygenase pathway genes suggests the catabolism of linoleic acid as the mechanism of eight-carbon compound production. This is the first fungal genome to be sequenced in the family *Helotiaceae*, and *A. sarcoides* was isolated as an endophyte, making this work also potentially useful in fungal systematics and the study of plant–fungus relationships.

## Introduction

Global climate change and decreasing fuel reserves are driving a push towards biologically derived fuels from plant wastes. The optimal biofuel for immediate implementation is one that functions within the context of current infrastructure, in particular with existing engines and distribution systems. This would require chemical similarity to gasoline, which is a mixture of hydrocarbons with an average chain length of eight [1]. Fungi have been recognized as producers of eight carbon (C8) volatiles for nearly 80 years and are a major global carbon recycler [2],[3]; however, despite the interest in these compounds, the genes responsible for their production remain largely undefined.

One such producer of C8 volatiles is the endophyte *Ascocoryne sarcoides* (NRRL 50072). Originally identified as *Gliocladium roseum*, this organism was shown to produce a series of molecules of potential interest as biofuels when grown on a cellulose-based medium [4]. The taxonomy was later revised to *A. sarcoides* and its production profile of Volatile Organic Compounds (VOCs) was amended to remove branched-chain alkanes. However, this follow-up work also confirmed the production of straight-chain alkanes from C6 to C9, as well as branched-chain alcohols varying in length from C3 (2-methyl-1-propanol) to C7 (5-methyl-1-hexanol) (Table S1) [5]–[7]. Understanding and optimizing biological production of such molecules is an area of active research (reviewed in [8]).

Bacteria have been shown to produce alkenes through “head-to-head” condensation of fatty acids; however, products with fewer than 23 carbons, like those from *A. sarcoides*, are not known to be synthesized by this mechanism [9],[10]. Odd-chain alkanes and alkenes of chain lengths 13–19 have been observed in bacteria as products of the decarbonylation of aldehydes and the decarboxylation of fatty acids, respectively [11],[12]. However, currently there are no known eukaryotic homologs for these enzymes. C8 alcohols and ketones have been identified as the products of linoleic acid breakdown; however, the genes responsible for the downstream reductions that generate C8 alkenes and alkanes are still unknown [13]–[17]. In order to gain a better perspective on these pathways and the cellulolytic machinery used by an endophyte, we coupled genome sequencing and short and long RNA-seq with metabolomic profiling of *A. sarcoides*.

Generation of metabolic pathway predictions in organisms for which genetic tools have not yet been developed remains a difficult problem. Techniques such as gene expression analyses and metabolomics profiling have the advantage that genetic tractability is not required. In a pioneering study, Askenazi *et al*, showed that gene expression could be linked to specific metabolite production [18]. The authors profiled the level of lovastatin production in engineered strains of the fungus *Aspergillus terreus* and showed that strains with similar transcriptional profiles also had similar amounts of lovastatin production [18]. Furthermore, extensive metabolic network analyses have demonstrated the ability to link the transcription of individual genes to metabolites [19],[20]. Metabolite-transcriptional coupling has since been validated extensively for the monitoring of different stress responses [21]–[23].

We used RNA-seq based gene expression measurements to accurately map gene structures and to generate candidate gene lists for novel metabolic pathways. In particular, we used gene expression and the co-occurrence of a compound across multiple experimental perturbations to generate candidate genes and pathways for the production of C8 volatiles and several other alkanes and alkenes that currently have no known eukaryotic pathway. In addition, we extensively mapped and annotated the *A. sarcoides* cellulose breakdown machinery using RNA-seq expression analysis after growth on different carbon substrates. Together with the high quality genome assembly and annotation, these data provide the most complete genomic characterization of any fungal endophyte to date. The analyses and datasets contribute to the development of biofuels from microbial metabolites and the related study of cellulose degradation and may be a reservoir of information for studying the plant-endophyte relationship.

## Results

### Genome assembly and annotation

**Figure 1:**
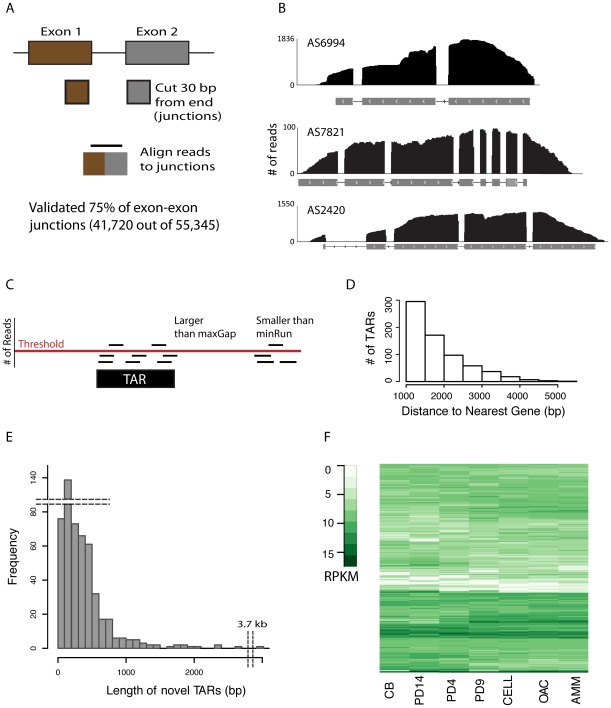
Validating gene models and novel TARs. (A) Schematic showing splice junction library generation. (B) For each of the three gene models shown, the x-axis is the genomic coordinates and the gray boxes represent individual exons, with arrows indicating strand. Reads having any overlap with the genic region are represented by black lines, the height of which correspond to the number of reads covering a particular base pair. Note that a read can align outside the exonic region, but that this was not observed at intron boundaries, although it did occur in the UTRs. (C) Schematic illustrating *de novo* assembly of reads into transcriptionally active regions (TARs). Three parameters are shown: threshold, min run, and max gap. Threshold sets the number of reads required for the region to be considered in the assembly. minRun sets the number of base pairs in the contiguous region required, and maxGap sets the number of discontiguous base pairs permitted to still be considered part of the assembly. Only the black box has sufficient base pairs above the threshold with the permitted contiguous length to be considered a TAR. (D) The minimum distance between each TAR and its nearest neighboring gene was computed. The number of TARs at least 1 kb away from any gene are shown (novel TARs). (E) Histogram of the length of novel TARs. Note the break in both the x and y-axis to indicate the outliers for TAR length and frequency. (F) Columns represent the culture growth conditions, rows individual novel TARs, and elements are color coded according to their RPKM value from white (no expression) to dark green (high expression).

The *A. sarcoides* NRRL 50072 genome was sequenced resulting in approximately 38-fold coverage of the estimated 34 Mb genome [24]. Reads were assembled into 16 scaffolds incorporating 99.5% of the total genomic base pairs. The genome size and overall GC content (45%) is within the average range for other Leotiomycetes fungi [25]. We predicted 10,831 genes resulting in 100% recovery of annotated Core Eukaryotic Genes Mapping Approach (CEGMA) genes which is a benchmark for a high quality genome assembly (Text S1) [26]. Roughly 70% of the gene models had at least one match to one of the 42 available fully sequenced fungal genomes. Approximately 22% of the gene models are seemingly species-specific and did not match to anything currently in GenBank [27]; the remaining 8% were homologous to genes outside of the fungal kingdom. Eighty-seven percent of the gene models were validated with long-read transcriptome profiling (Text S1) and 75% of the potential exon-exon junctions were confirmed (see Figure 1A and 1B). Although a subset of the unvalidated gene models and exon junctions may be spurious, the majority are most likely true genes that are silent under these specific conditions [28],[29].

### RNA-seq analysis and novel TAR identification

We subjected *A. sarcoides* to seven different growth conditions to assay diversity in both transcription and compound production (Table S2). Volatile metabolite production was analyzed by gas chromatography mass spectrometry (GC/MS) for six of these seven conditions (no GC/MS dataset was obtainable on the day 9 potato dextrose harvest; Table S1 and S2). We monitored *A. sarcoides* cultures for production of volatiles and selected this subset of six conditions for RNA-seq analysis, which provided differential compound production profiles. Under these six conditions, *A. sarcoides* produced 48 identifiable volatile metabolites including 18 alcohols and 7 alkanes/alkenes including heptane, octane, and nonane. All volatile metabolites were scored with a binary scale to indicate their presence or absence in each culture headspace. We chose this digitized scoring because different analyses required variation in culture and headspace volumes and our method of detection of VOCs (Solid Phase Micro Extraction (SPME), see Materials and Methods) is sensitive to such variation [30]. The large number of functionally diverse metabolites in the headspaces also precluded the use of external or internal standards to determine the absolute amount detected for each compound across all conditions.

Coupled transcriptional profiles for the six conditions obtained via RNA-seq resulted in more than 200 million reads alignable to the reference genome or exon junctions (Table S3) and greater than 99% similarity between the two technical replicates (Figures S1 and S2). Six diverse sampling conditions were chosen for the RNA-seq analysis *in lieu* of replicates in order to more thoroughly explore the transcriptional landscape of *A. sarcoides* and more completely map gene structure throughout the genome. The genome and transcriptome data can be accessed at http://asco.gersteinlab.org.

In addition to the 10,831 gene models predicted, we identified a number of RNA-seq reads which map outside of the gene models. A subset of these reads formed well-defined regions on the reference genome. 602 of these regions are at least 1 kb away from any annotated genes and are designated as transcriptionally active regions (TARs) (Figure 1C, Figures S3 and for sensitivity analysis and examples). These TARs were seemingly devoid of open reading frames and in some cases were quite long (up to 3.7 kb in length). Forty percent of these TARs illustrated condition-specificity (standard deviation greater than 1; see Figure 1D–1F) as has previously been observed in *S. cerevisiae* and *H. sapiens* [31]. The importance of these polyadenylated non-coding RNAs in regulating gene expression has only recently been discovered [31],[32] and their exact role remains an active area of research.

### Annotation and expression of cellulose degradation machinery

**Figure 2:**
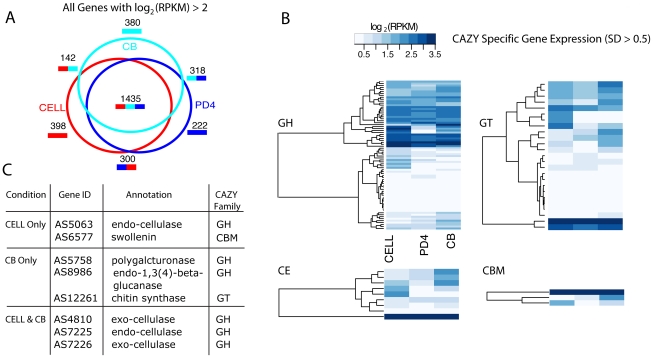
Analysis of cellulose-related expression. *A. sarcoides* transcription was profiled when grown on potato-dextrose media for 4 days (PD4), cellulose (CELL) and cellobiose (CB). (A) The total number of genes with quantile normalized log_2_(RPKM) greater than 2 was computed for each condition. The venn diagram shows the overlap of these genes across the three conditions. (B) Genes were partitioned according to their homology to the four main CAZY families: Glycoside Hydrolase (GH), Glucosyl Transferase (GT), Carbohydrate Esterase (CE), Carbohydrate Binding Modules (CBM). The homologs were then filtered to include only those genes which showed a standard deviation across the three conditions greater than 0.5. Each family was separately clustered (hierarchical, Euclidean distance, single linkage). The colorbar represents the quantile normalized log_2_ (RPKM) value from white (low expression) to dark blue (high expression). Note: CBM can co-occur with all families. Only those genes that had exclusively a CBM domain were clustered in the CBM matrix to avoid duplication. (C) A table of the most highly expressed genes includes genes not directly involved in degradation, such as swollenin and chitin synthase (see Results for more details).

Given the emphasis on cellulose breakdown and utilization for the development of alternative fuels, we were interested in exploring and annotating the cellulolytic capabilities of *A. sarcoides*. We analyzed the transcription profiles of *A. sarcoides for* growth on three different carbon sources: cellulose (CELL), cellobiose (CB), and potato dextrose (PD4). While cellulose and cellobiose share the same β(1–4) linkage between monomer units, potato dextrose contains predominantly glucose-monomer. Differential gene expression between the potato dextrose and the two more complex substrates (CELL and CB) provides information on the pathways and mechanisms of cellulose breakdown; whereas, differences between the CELL and CB provides information on the genes necessary to utilize a soluble versus an insoluble polymer. Such differences are particularly useful as they can inform methods aimed at increasing cellulose breakdown efficiency. We first examined the differential expression across these three conditions (Table S15) [29],[33]. There were 1,435 genes that were expressed under all three conditions (Figure 2A). A smaller number, 142 genes, were only expressed during growth on cellulose or cellobiose, including the endo- and exo-cellulases, as expected based on their role in cellulose utilization. 398 and 380 genes were exclusively expressed on cellobiose and cellulose, respectively, reflecting the significant differences in the machinery necessary to utilize a soluble disaccharide versus an insoluble polymer and in the resulting downstream changes in the cellular state. We focused on the subset of genes with homologs in the CAZY database, a manually curated repository for carbohydrate metabolism (see Text S1) [34]. In total, 52% (89 of 169) of glycosyl-hydrolase homologs (GH), 45% (25 of 56) of glycosyl-transferases (GT), 50% (3 of 6) of carbohydrate-binding module genes (CBM), 41% (9 of 22) of carbohydrate esterases (CE), and 0% (0 of 1) of polylyase (PL) were differentially expressed across the three conditions (Figure 2B; Table S4).

The most highly expressed gene in the cellulose condition was AS6577, which is homologous to the gene encoding the protein swollenin. Swollenin was first identified in the cellulolytic model organism, *Trichoderma reesei*. Heterologous expression in yeast and *Aspergillus niger* showed that swollenin mediates disruption of plant cell walls without releasing monomeric sugars [35]. Supplementation of a cellulase mixture with swollenin increased saccharification rates suggesting this protein may play an important role in efficient cellulose breakdown [36]. While *A. sarcoides* growth on a lignin-containing medium was not analyzed, we identified the full pathways for 5-carbon sugar utilization e.g. arabinose and xylose, sugars which comprise 10–25% of carbohydrates resulting from hemi-cellulolysis [37]. We further validated the presence of these pathways by demonstrating *A. sarcoides* growth on media with either xylose or arabinose as the sole carbon source (Materials and Methods).

### Identification of genes for biosynthesis of secondary metabolites

The genes responsible for both cellulose degradation and the production of secondary metabolites are non-randomly distributed in a number of sequenced genomes, such that they are clustered into regions of higher than average gene density [38],[39]. Therefore, we searched for clusters in *A. sarcoides* as a strategy to identify genes involved in these processes. We generated a simulated set of scaffolds where the number of genes was kept constant but the placement was randomized to identify regions of the genome with higher than expected gene density. We identified 77 clusters ranging in length from 10–72 kb (*p*<.05, Text S1). Twenty-six clusters contained genes or domains known to be involved in secondary metabolism, particularly oxidoreductases and permeases. We noted five gene-clusters that were involved in the production of secondary metabolites usually restricted to plants, including two clusters containing genes homologous to those involved in the synthesis of patatin (Table S5). Patatin is a plant storage glycoprotein implicated in plant-fungal communication [40]. Expression of this protein in *Arabidopsis* negatively affects resistance to *Botrytis cinerea* and *Pseudomonas syringae*, but it increases resistance to the cucumber mosaic virus [40]. Interestingly, all genes in this cluster were transcriptionally silent under the conditions we tested. Given their known functional role in mediating plant-fungal interactions, it is possible they are strictly regulated by interactions with the plant host.

The classes of genes most frequently involved in secondary metabolite production are Polyketide synthases (PKS) and Non-ribosomal peptide synthetases (NRPS). We identified 19 PKS and NRPS clusters through fungal-specific Hidden Markov Models of beta ketoacyl synthase (KS) and acyltransferase (AT) domains and an additional 8 gene clusters and 11 gene models composed solely of enoyl reductase and/or dehydratase accessory domains (Text S1, Tables S6 and S7). The identified PKS genes ranged in size from a few kb to the 13 kb and 13-exon hybrid PKS/NRPS AS8071, which is by far the largest predicted gene model in *A. sarcoides*. Examination of the 3 kb region upstream and downstream of each PKS element also revealed a number of major facilitator superfamily transporters and permeases which may confer resistance to both PKS-derived and exogenous toxins [41]. However, comprehensive searches of previously identified PKS clusters [42], *laeA* element identification to delineate possible cluster boundaries [43], and use of domain to structure software [44] failed to yield any predictions for possible biosynthetic products. Intriguingly, one PKS, AS1082 was first found to contain a beta ketoacyl synthase domain, but subsequent searches revealed that it contained two distinct KS domains and an acyl carrier protein domain. However, no acyl transferase domain, which typically functions in substrate loading, was identified. While separately encoded acyl transferase enzymes that act *in trans* have been found in bacteria, only *trans*-acting enoyl reductase domains have yet been characterized in fungi [45].

### Correlating VOC production with gene expression to elucidate biosynthetic pathways

**Figure 3:**
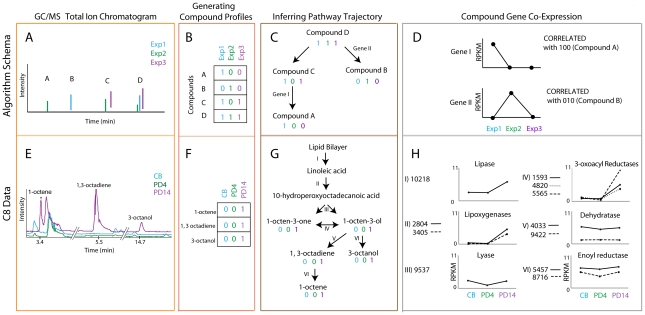
Coupled transcriptomics and metabolomics to generate pathway predictions. The top panels (A–D) represent the algorithm schema and the bottom panels (E–H) represent the corresponding steps with data for an example pathway, C8 production. Cyan, green, and purple are used to denote different experimental conditions (1, 2, and 3 and CB, PD4, and PD14 for the schematic and the C8 pathway data, respectively). GC/MS total ion chromatograms (orange box, A & E) are used to generate compound co-occurrence profiles (red box, B & F). These compound co-occurrence profiles are used to group and order the compounds based on patterns of correlation and anti-correlation to build a possible biosynthetic pathway (brown box C & G). Genes for which the expression profile matches the compound profile are considered correlated and therefore likely candidates for the biosynthetic pathway of interest (gray box D & H). Retrosynthesis is then used to match correlated genes with a reaction in the pathway, represented by roman numerals denoted on pathway arrows (brown box, C&G).

A more direct method to investigate the *A. sarcoides* genes responsible for production of the novel metabolites is the use of association analysis. As mentioned above, the concordance of gene expression and metabolite production can be used to guide prediction of genes involved in metabolic pathways [18]. A complication in the application of these methods for novel metabolic pathways, as opposed to those generated either via PKS or as part of conserved metabolism, is that we know neither the genes that are involved nor the pathway structure (i.e. the reactant-product pairings that result in the downstream compound). For example, we do not know the genes responsible for the production of octane, nor do we definitively know the starting compound or what intermediates may have been subsequently generated. Thus, we need a series of analyses that simultaneously infer the potential genes and the pathway trajectory as defined by the chemical elements (Figure 3A–3D; Figures S5, S6, S7).

It was previously shown that by examining the “correlation” and “anti-correlation” of sets of genes across a wide spread of phylogenetic space, the importance, ordering, operons, and additional members of the pathway can be discerned [46]–[50]. Furthermore, genes belonging to the same pathway or complex often show both coordinated regulation and conservation [50]. By substituting the phylogenetic profiles from these previous studies with our compound profiles generated from compound presence or absence across all conditions, the resulting character matrix can be used to determine the relatedness of these compounds (Figure 3B and 3F, Figures S7 and S8). On the basis of these relationships, compounds can be then grouped into pathways. To apply this correlation analysis, each metabolite produced by *A. sarcoides* under each of the six growth conditions was assigned a “1” if it was detected in the particular condition and “0” if it was not detected, as depicted in the schema in Figure 3A–3B and 3E–3F). To further inform the metabolite analysis, we also used a recent meta-analysis that profiled the production of 10 *Ascocoryne* isolates under varying growth conditions resulting in 20 different GC/MS profiles [5]. Compounds that consistently co-occurred across the genus are more likely to be in the same pathway and were given more weight than those showing inconsistent behavior (Figure S7). We then grouped sets of compounds that co-occurred into single or related sets of pathways (Figure 3C, compounds A and C) and those that rarely or never co-occurred into different pathways (Figure 3C, compound B). To identify possible metabolite-gene linkages, we then computed the correlation between the compound profile and expression of each gene under the different conditions. Correlations between compounds and expression were used instead of strictly quantitative changes in gene expression because this more effectively integrated the expression analysis with the binary compound production data. To ensure the correlations were significant, we computed a *p*-value for the compound co-expression scores (See Text S1, Tables S8, S9, S10, S11, S12, S13). For a set of compounds with the same compound profile, there may be many genes with correlated expression, not only those involved in the compound production. Therefore, retrosynthesis was used to disambiguate which of the significantly correlated genes were most likely involved in the production of those compounds (Figure S8).

As one example of this method identifying candidate genes, we identified 60 genes with homology to putative alcohol dehydrogenases (EC 1.1.1.1), which have a wide range of specificities and annotation quality. However, only three of the identified alcohol dehydrogenases were significantly co-expressed with any compound production profile. In particular, AS5307 was co-expressed with the compound profile that had a predominance of branched medium chain alcohols, including 3-methyl butanol, 3-methyl-3-buten-1-ol, and 2-methyl-1-propanol. We predict that these three dehydrogenases, from amongst the 60, play a key role in the production of the observed medium-chain alcohol metabolites.

**Figure 4:**
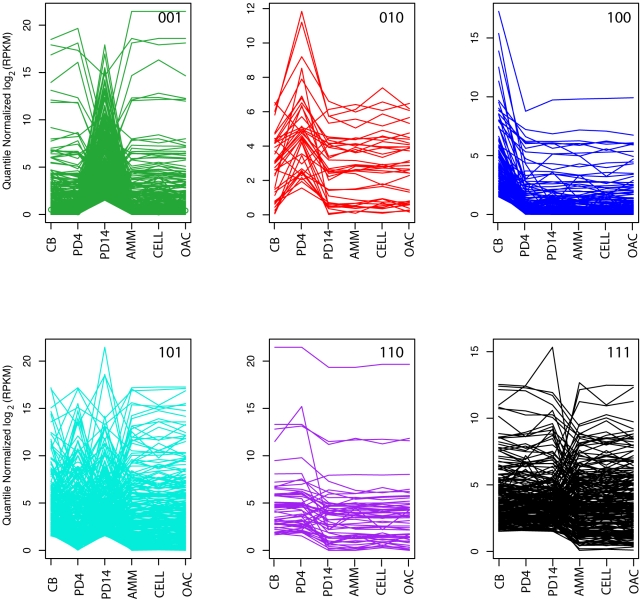
Compound gene co-expression profiles. Each plot shows the quantile-normalized log_2_ (RPKM) for each set of genes of co-expressed with a particular compound profile (green 001, red 010, blue 100, cyan 101, purple 110, and black 111) across all 6 conditions (CB, PD4, PD14, AMM, CELL, and OAC). The first three conditions (CB, PD4, and PD14) represent the conditions where the compounds analyzed in this study were detected. The remaining conditions serve as the nulls (see Text S1for details). Within the plots, each line corresponds to a single gene.

Co-expression has been used to assign functions to genes with known homologs as well as to genes without primary sequence or domain level annotations [51]. All genes co-expressed with a particular compound profile were examined as shown in Figure 4, where each line represents a single gene. A subset of the genes was homologous to well-known secondary metabolite pathway elements, but some had no known function (Figures S9, S10, S11). In the latter cases, gene co-expression was used to infer additional pathway elements as well as associated regulators and transporters. Below, we provide an example set of predictions for a C8 product pathway. The full set of predicted pathway schemas and potential enzymes are provided in the supplement. An R package containing the code and documentation for RNA-seq processing and the association analysis is provided in Text S2.

### Pathway predictions for biosynthesis of C8 metabolites

Given the average chain length is about eight for hydrocarbons in gasoline, the production of molecules with similar lengths represents an obvious starting point for next generation biofuels that will be compatible with pre-existing infrastructure [52],[53]. We identified candidate pathway elements for the production of reduced C8 volatiles in *A. sarcoides* and assigned correlated genes to each step of the reconstructed C8 pathway (Figure 3). As an example, lipoxygenases (EC 1.13.12.12) are known to be involved in the formation of C8 alcohols and ketones in fungi via the catabolism of linoleic acid [3],[54]. There are five lipoxygenases in the *A. sarcoides* genome, and two of these are correlated with C8 production (AS2804 and AS3405, Figure 3H, II). The most strongly correlated lipoxygenase, AS2804 is homologous to the *Aspergillus* gene *ppoC* (Figure 3H, II) (*p*<.01). Recently, Brodhun *et al* showed that expression of *ppoC* is sufficient to catalyze the breakdown of linoleic acid into a wide range of compounds including: 1-octen-3-ol, 2-octen-1-ol, 2-octenal, and 3-octanone in a crude *E. coli* lysate [17]. All of these compounds were observed as products of *A. sarcoides* with the exception of 2-octenal (Table S1). The original hypotheses for the production of these C8 volatiles from linoleic acid involved two enzymes, a lipoxygenase to form a peroxidated intermediate, and a lyase (EC 4.1.2.-) to catalyze its breakdown into smaller, volatile products. However, an active lyase has yet to be successfully purified in fungi [14]–[16],[55], and recent work argues against the need for this activity [17]. We identified one lyase, AS9537; however, its expression did not correlate with the production of C8 volatiles (Figure 3G, III), arguing against the dual-enzyme hypothesis for C8 production and supporting the more central role for the lipooxygenase (AS2804).

In addition to the oxygenated C8 volatiles observed by Brodhun *et al.* from *Aspergillus*, *A. sarcoides* produces the reduced compounds 1,3-octadiene; 1,3-*trans*-5-*cis*-octatriene; 1,5-octadien-3-ol; 1-octene; and 3-octanol suggesting that downstream processing of linoleic acid breakdown products has occurred. One potential route to these compounds is shown in Figure 3G, whereby 1-octen-3-one is further reduced to 1-octen-3-ol by FabG (EC 1.1.1.100), a 3-oxoacyl-[acyl-carrier protein] reductase (Figure 3G, IV). In total, *A. sarcoides* has 10 genes with strong homology to FabG (Table S14), however, only the three co-expressed with C8 production are shown in Figure 3H, IV (AS1593, AS4820, and AS5565, with AS5565 exhibiting the largest expression change). The nearest sequenced relatives of *A. sarcoides*, *Botryotinia fuckeliana*, and *Sclerotinia sclerotiorum*, have only two and three FabG genes, respectively (Figures S13 and S14). Since the reduced C8 compounds have not previously been found outside the *Ascocoryne* genus and the expression of some FabG genes do correlate with these compound production profiles, it is possible that at least some of these additional FabG genes may participate in the reduction of eight carbon volatile compounds. In addition to the FabG homologs, 317 oxidoreductases particularly aldo-keto reductases, were identified in *A. sarcoides*. Of these 11 were correlated with C8 production (Table S14). Oxidreductases are able to reduce various functional groups, such as ketones and alcohols, and are expected to participate in the biosynthesis of the C8 reduced products and other volatiles (Figure 3G, 3H; IV and VI). In addition, of all sequenced fungal genomes, only *A. fumigatus* (626) and *T. reesei* (494) have a commensurate number. Both *B. fuckeliana* and *S. sclerotiorum* have less than 200 oxidoreductases, which is approximately the median number for sequenced fungi. The above average number of oxidoreductases found within *A. sarcoides* suggests a large reducing capability and extensive secondary metabolism potential.

## Discussion

The unknown pathway for the production of potential biofuel compounds in *A. sarcoides* is part of a more general trend. Microorganisms produce an extraordinary diversity of natural products that have the potential to be used in numerous applications from medicines to biofuels to commodity chemicals [37],[56],[57]. However, identifying the genes responsible for their production remains a major hurdle for organisms that are not genetically tractable. Despite promising developments in pathway prediction algorithms, a substantial gap remains between metabolic capabilities and genetic characterization [58]–[61]. As an example, Metacyc, a repository of metabolic pathways, contains 8,869 compounds linked to 1,908 known pathways, but this represents less than 1% of the compounds estimated to be produced by micro-organisms [62],[63]. An integrated omics approach could provide a relatively simple means of exploring the biosynthetic potential of uncharacterized non-model organisms.

By examining changes in the *A. sarcoides* transcriptome across a diverse array of conditions, we were able to explore a wide fraction of genes and refine gene and exon boundaries to improve the genome annotation quality. Additionally, with co-expression patterns we generated hypotheses for the genes involved in undefined metabolic pathways and regulatory mechanisms. Through TAR building we identified a number of long, highly expressed regions seemingly devoid of open reading frames that may have a regulatory role. The recovery of 100% of all CEGMA [26] genes suggests a high quality genome assembly, and the number of scaffolds is on par with the number of expected chromosomes in Ascomycete fungi [64]. We used an expanded version of association analysis to generate hypotheses for products from unknown pathways. Such methods are flexible enough to integrate coupled transcriptome and metabolomics data and will take on increasing importance as the throughput of both transcriptome and metabolomics continues to increase. The means to leverage these datasets will be key to our understanding of novel metabolite production particularly for genetically intractable organisms. From its plant mediators to its oxidoreductases and its cellulases, *A. sarcoides*'s gene complement represents several avenues for further research and its diverse array of enzymatic capabilities will contribute to the study of cellulose degradation and secondary metabolite production.

## Materials and Methods

### Genomic DNA isolation

Isolate NRRL 50072 was obtained under a material transfer agreement from Montana State University (GA Strobel, Bozeman, MT). Genomic DNA was isolated using the Plant DNeasy MaxiPrep kit (Qiagen) according to the manufacturer's instructions with the following modifications: mycelia were grown in potato dextrose broth for approximately 3 weeks at 25°C, shaking at 150 rpm and were harvested via filtration. The filtrate (1 g) was homogenized by mortar and pestle under liquid nitrogen before the addition of 80 µL RNase (100 mg/mL), 80 µL proteinase K (10 mg/mL) and lysis buffer P1 (Qiagen). Homogenized material was heated for 10 min at 65°C and then processed through the remainder of the Qiagen protocol.

### Sample preparation of RNA for Illumina RNA-seq

Please see Table S2 for detailed growth and inoculation conditions for CB, PD4, and PD14 as distinguished by the short code referred to in both the text and figure legends. For the remaining 3 conditions (OAC, AMM, and CELL), media were prepared and inoculated with 50 mg filtered culture (1× PD) as reported in Griffin et al., 2010 [5]. Carbon starvation (OAC) was prepared as a minimal medium base with sodium acetate (50 mM) as the sole carbon source. Nitrogen starvation (AMM) was prepared as a minimal medium base with no ammonium chloride and with 83.3 mM glucose. Cellulose substrate (CELL) was prepared as a minimal medium base with cellulose (15 g/L) as the sole carbon source. All were titrated to a pH to 6.0 with NaOH. Vials were incubated for 2 days at 23°C before GC/MS analysis and RNA extraction. For each of these conditions, seven vials were inoculated, with three subjected to GC/MS analysis while the remaining four vials were concurrently used for RNA harvesting. Total RNA was isolated using the Ambion RiboPure Kit (California, USA), and then poly-A purified and prepared for sequencing as in Nagalakshmi *et al.*, 2008 [28].

### Sample preparation of RNA for long-read transcriptome (454)

Sample PD9 was selected for RNA preparation and long-read transcriptomics, which was used to confirm gene models. RNA was extracted from a 9-day old 1 L PDB culture grown at 23°C, 150 rpm (Table S2). Extraction performed as in Nagalakshmi et al., 2008 [28].

### Metabolomics profiling

All conditions were as specified under the RNA-seq preparation. GC/MS was carried out in parallel with cultures harvested for RNA seq with the exception of PD9, which was not profiled. Control samples for each media condition were prepared for use in GC/MS analysis with the same methods as described in the RNA seq conditions section above, but without the addition of inoculums. Analysis of culture headspaces was performed on a gas-chromatograph coupled to a time-of-flight mass spectrometer (GCT Premier, Waters). Automated culture sampling was mediated by a CTC CombiPAL Autosampler (Leap Technologies) and all cultures were sampled with a 50/30 µm divinylbenzene/carboxen/polydimethylsiloxane StableFlex Fiber (Supelco). GC injection and column parameters, GC temperature program and MS data acquisition parameters were as described previously [5]. Parameters for SPME headspace sampling were as follows. OAC, CELL, and AMM vial cultures were analyzed via automated sampling with a pre-extraction SPME fiber conditioning (7 min, 250°C), 35 min headspace extraction at 30°C, and a splitless GC injection (30 sec, 240°C, 0.75 mm ID injection liner). Manual headspace sampling of CB, PD4, and PD14 flask cultures used the following sampling parameters: pre-extraction SPME fiber conditioning (12 min, 250°C), 30 min headspace extraction (room temperature, approximately 20–25°C), and splitless GC injection (30 seconds, 240°C, 0.75 mm ID injection liner). Data were analyzed with the MassLynx Software Suite™ (Waters). Chromatographic peaks were identified with a combination of spectral search comparisons with the Wiley Registry™ of Mass Spectral Data, 8th Edition, elemental composition analysis and the comparison of retention times and spectra with pure standards for compounds where noted (Sigma-Aldrich). Compounds identified during the analysis of control media samples, including contaminants resulting from the SPME fiber and Wax capillary column, as well as media derived compounds, were excluded from the final compound report for each condition. See Table S1 for the full compound profiles.

### C5 and C6 growth assays

Growth assays were performed in 96 well plates in 200 µL media containing trace metals as in Griffin *et al.*, 2010 [65], 0.67 g/L Yeast Nitrogen Base (Difco) and supplemented with 100 mM of either glucose, xylose, arabinose, mannose, cellobiose, or sodium acetate, titrated to a pH of 6 with KOH. Wells containing no added carbon source served as the control. The cultures were inoculated by adding 5 µL of 5×10^7^ spores/mL in Phosphate Buffered Saline (Gibco), and the cultures were grown for 5 days at 23°C. Growth was determined by visual inspection.

### Genome assembly and annotation

Initial assembly with single end shot gun titanium reads with Roche's GS DeNove Assembler (Newbler) resulted in 137 scaffolds with an N50 of 2.8 Mb [24]. Following addition of a paired end 3 kb-insert sequencing run, these were assembled into 16 scaffolds encompassing 99.5% of the total sequenced base pairs. Called genes were first aligned to the GenBank non-redundant database using blastx (v2.2.24) [27],[66]. A hit was defined as a match when overlap with the length of the query protein was greater than 60% and E-value<1e-10. We extracted the subset of genes found in the CAZY database, a repository for manually curated carbohydrate machinery, and performed a similar procedure [67].

Domains were identified using the hmmsearch function from HMMer [68] with both a set of fungal-specific protein domains [69] and the entire PFAM database [70]. A domain was considered a match if the E-value was greater than 1 and the length of the match was at least 15. Pathway predictions and enzyme classification was completed through KEGG/KAAS [71],[72]. GO predictions were made by first mapping the set of *A. sarcoides* genes to their corresponding *Aspergillus nidulans* homolog [73],[74]. Please see Text S1for a full description of the gene cluster and PKS/NRPS identification strategies.

### RNA-seq analysis

In the case of the Illumina runs, mapping was done via building bowtie indices for splice junction libraries, and the genome respectively using default parameters (tolerated up to 2 mismatches and screened for quality scores) [75]. Splice junction libraries were generated as described in Habeggar et al., with 30 bp exon ends [76]. The bowtie reads were converted to mapped read format (MRF) and mrfQuantifier was used to compute a variation of reads per kilobase of exon per million mapped sequence reads (RPKM) for each gene using RSEQtools [76]. Briefly, we computed RPKM as the number of nucleotides that map per kilobase of exon model per million mapped nucleotides for each gene rather than the read count. It is computed by summing the total number of reads that cover each base pair of an annotation feature of interest (in this case of exons) and normalizing by the total length of the feature. For the conditions denoted by CELL, OAC, and AMM, technical replicates were performed yielding one lane per replicate. In the case of PD4, there were two technical replicates performed 2 months apart. A comparison of the RPKM of the genes between lane replicates showed greater than 99% agreement (Figure S2), although the correlation was slightly less between the two AMM replicates than between any other conditions.

The 454 long reads (average size 410 bp) were mapped against the gene models and the genome using BLAT with default parameters [77]. In all cases, only reads that unambiguously mapped to a single location were used for the downstream analysis. For each gene, we calculated the RPKM score as described above. To estimate depth of coverage, the percentage of genes that were detectable using subsamples of reads was computed where detectable was defined as having at least 1, 2, 5, or 10 reads, respectively, overlapping the gene (Figure S12).

### Identification of transcriptionally active regions (TARs)

A database of transcriptionally active regions (TARs) was constructed from those RNA-seq long reads that map uniquely to the genome via BLAT [77]. The TAR database was built by employing the minrun/maxgap segmenting module [76]. Gene coverage values were calculated for a range of minrun/maxgap parameters to assess their impact on observed gene coverage. Included in the coverage analysis were TAR file sets with maximum read gaps between zero and five and minimum read run from 30 to 40 (See Text S1for a full description; Figures S3 and S4).

## Supporting Information

Figure S1 All versus All comparison of transcriptional profiles for Illumina runs in all seven different growth conditions. Axes are log_2_ RPKM values.
(PDF)

Figure S2 Technical Replicates of Illumina transcription data. There is greater than 99% similarity between the two replicates for each of four conditions.
(PDF)

Figure S3 Determining the appropriate parameter values for identifying Transcriptionally Active Regions (TARs). Each line represents a particular threshold in the TAR sensitivity analysis (see legend for Threshold levels). The x-axis is sorted first by the MaxGap value (0,1,2,3,4,and 5) and secondly, for each by the MinRun length value (30, 35, 40).
(PDF)

Figure S4 Examples of novel TAR read stacks. The top panel illustrates a well-defined novel TAR that is most likely a missed gene call. The bottom panel demonstrates another novel TAR that is at least 1 kb away from any annotated genes and yet remains part of a long expression tail adjacent to an actively translated exon.
(PDF)

Figure S5 Hypothetical compound gene co-expression profiles demonstrating the need to include null data sets. (A), the purple line and the green line (square and circle, respectively) represent two possible compound profiles (110 and 001). The gene expression of the black line (diamond) is equally correlated with the two compound profiles. (B) A null condition is included in the analysis (condition ID 4). The best compound profile match for the gene's expression pattern (black line) is now the green line (0010). Addition of the null condition permits the distinction between the two compound profiles. See text for more details.
(PDF)

Figure S6 Inferring Product/Reactant Pairs through Compound Profile Consistency. We illustrate this idea with a simple color experiment. As an example, a purple precursor (110) can result in blue (100), red (010), or purple (110) products, but not a yellow (011) product.
(PDF)

Figure S7 Clustering Compound Co-occurrence. Compounds are clustered based on their co-occurrence as measured by their DLW distance across the whole *Ascocoryne* genus. Compounds are colored according to their production profiles (using the same color scheme in Figure S6): Green 001, Red 010, Blue 100, Cyan 101, Purple 110 and Black 111. Compounds in Brown were reported in the previous analysis of the *Ascocoryne* genus for VOC production, but not presently detected in any of the conditions linked with RNA-seq data.
(PDF)

Figure S8 Retrosynthesis of *A. sarcoides* products. Compounds are colored by their associated profile as defined in Figure S6. Brown indicates those compounds that were previously detected from the *Ascocoryne* genus, but were not detected during the present the RNA-seq coupled analysis. Gray represents compounds that have never been detected, but are proposed intermediates. (A) Hypothetical schema for producing alkanes from a ketone precursor. (B) Hypothetical schema for converting an aldehyde into the corresponding alkane, as well as possible off-pathway reactions that produce an ester.
(PDF)

Figure S9 Clustered Gene Co-expression 001. Each of the 3 following figures (Figures S9, S10, S11) was generated as follows. The genes whose expression correlated with a particular compound profile were partitioned using k-means clustering into sets of genes co-expressed across all 6 conditions. Each graph represents the gene expression of a single cluster where the x-axis is the Condition Id and the y-axis is the Quantile Normalized log_2_ RPKM. The 001 genes partitioned into two clusters, representing up and down regulation states. However, more complex partitioning occurred for the 101 and 111 profiles.
(PDF)

Figure S10 Clustered Gene Co-expression 101. As described for Figure S9, this illustrates the gene expression patterns for all genes correlated with the 101 compound profile, where each plot represents a single cluster of genes.
(PDF)

Figure S11 Clustered Gene Co-expression 111. As described for Figure S9, this illustrates the gene expression patterns for all genes correlated with the 111 compound profile, where each plot represents a single cluster of genes.
(PDF)

Figure S12 The fraction of genes detected with 1, 2, 5, and 10× read coverage, respectively, at different sub-samplings of the 454 long reads.
(PDF)

Figure S13 Metabolic mapping of KEGG orthologs for *A. sarcoides*, *S.sclerotiorum*, *G. zeae*, *S. cerevisiae*. Nodes are compounds and connecting lines are enzymes. Color codes are based on functional category. A node can appear in multiple places. The tree is just for illustrative purposes; the branch lengths are not drawn to scale. Generated via iPath.
(PDF)

Figure S14 (A) A representative image of the synteny between *S. sclerotiorum* and *A. sarcoides*. The *A. sarcoides* scaffolds are stacked on the right-hand side and *S. sclerotiorum* scaffolds are shown in the colored inset. Like-colored regions of *A. sarcoides* scaffolds and those of *S. sclerotiorum* represent syntenic blocks. (B) Table reports the total number of orthologs and the levels of synteny between *A. sarcoides* and each of the four fungi analyzed.
(PDF)

Table S1 Volatile compounds detected and identified via SPME-GC/MS from the headspace of NRRL 50072 samples. NRRL 50072 was cultured as described in the Materials & Methods and in Table S2 with the following conditions: Acetate (OAC), cellulose (CELL), cellobiose (CB), ammonium starvation (AMM), potato dextrose broth at 4 days (PD4), and 14 days (PD14). For each compound, 1 designates production/detection and 0 designates no detection. RT = retention time in minutes. Asterisk (*) designates compound retention time and EI spectra matched that of a pure standard.
(PDF)

Table S2 Culture growth conditions for GC/MS profiling and RNA preparation. NRRL50072 was cultured using the stated media, volumes, inoculation/growth conditions, and the RNA preparation and GC/MS analysis were performed on the days listed.
(PDF)

Table S3 Transcriptome Mapping Statistics. Report of Illumina and 454 reads mapped to gene models and to genome for each of the conditions and time points.
(PDF)

Table S4 The number of genes from each sequenced organism with homologs in the CAZY database per CAZY class. Glycosyl hydrolases (GH), glycosyl transferases (GT), Carbohydrate-binding module (CBM), carbohydrate esterase (CE), and polylyase (PL). Table structure adapted from Martinez *et al* [7].
(PDF)

Table S5 Genes identified in the *A. sarcoides* genome with homologs in plants. Genes are subdivided into three classes: P, genes with exclusively plant orthologs; M, genes with mostly plant orthologs; and N, genes that did not have a plant ortholog, but bordered a set of plant orthologs.
(PDF)

Table S6 Targeted search for β-ketosynthase (KS) and acyltransferase (AT) domains. Genes identified in the targeted search are listed with their domain annotations. For genes that were part of clusters, the additional genes found within the cluster are also included.
(PDF)

Table S7 Targeted search for enoyl reductase (ER), dehydratase (DH), and ketoreductase (KR) domains. Genes identified in the targeted search are listed with their domain annotations. For genes that were part of clusters, the additional genes found within the cluster are also included.
(PDF)

Table S8 Gene Subset Co-expressed with the 001 Compound Profile. For each of the following 6 tables (Tables S8, S9, 10, S11, S12, S13): Gene ID, gene ID within *A. sarcoides;* Status, reports if the gene is active (A) or repressed (R) in the production conditions; KO, KEGG ortholog ID; Description, description of the KEGG ortholog; EC, lists the Enzyme Commission number that corresponds to the KEGG ortholog, where relevant.
(PDF)

Table S9 Gene Subset Co-expressed with the 010 Compound Profile. Gene ID, gene ID within *A. sarcoides;* Status, reports if the gene is active (A) or repressed (R) in the production conditions; KO, KEGG ortholog ID; Description, description of the KEGG ortholog; EC, lists the Enzyme Commission number that corresponds to the KEGG ortholog, where relevant.
(PDF)

Table S10 Gene Subset Co-expressed with the 100 Compound Profile. Gene ID, gene ID within *A. sarcoides;* Status, reports if the gene is active (A) or repressed (R) in the production conditions; KO, KEGG ortholog ID; Description, description of the KEGG ortholog; EC, lists the Enzyme Commission number that corresponds to the KEGG ortholog, where relevant.
(PDF)

Table S11 Gene Subset Co-expressed with the 101 Compound Profile. Gene ID, gene ID within *A. sarcoides;* Status, reports if the gene is active (A) or repressed (R) in the production conditions; KO, KEGG ortholog ID; Desc, description of the KEGG ortholog; EC, lists the Enzyme Commission number that corresponds to the KEGG ortholog, where relevant.
(PDF)

Table S12 Gene Subset Co-expressed with 110 Compound Profile. Gene ID, gene ID within *A. sarcoides;* Status, reports if the gene is active (A) or repressed (R) in the production conditions; KO, KEGG ortholog ID; Desc, description of the KEGG ortholog; EC, lists the Enzyme Commission number that corresponds to the KEGG ortholog, where relevant.
(PDF)

Table S13 Gene Subset Co-expressed with the 111 Compound Profile. Gene ID, gene ID within *A. sarcoides;* Status, reports if the gene is active (A) or repressed (R) in the production conditions; KO, KEGG ortholog ID; Desc, description of the KEGG ortholog; EC, lists the Enzyme Commission number that corresponds to the KEGG ortholog, where relevant.
(PDF)

Table S14 Expression values (log_2_ RPKM Quantile Normalized) for the potential fabG genes (IP011284). For each Gene ID, expression levels are listed for each culture condition. Genes correlated with C8 production are highlighted in yellow.
(PDF)

Table S15 Summary Statistics of Differential Gene Expression. The number of genes expressed (quantile normalized log_2_RPKM) in each culture condition (Illumina RNA-seq) relative to expression in PD9 (454 long reads). Gene counts are given for six RPKM fold thresholds from 2 to −2.
(PDF)

Text S1 Supporting information including additional methods used for and results from the Expanded Association Analysis, Genome Annotation, Models and Clusters, Comparative Genomics, and Transcriptome.
(DOCX)

Text S2 An R package containing the Compound Context Analysis Code and documentation for RNA-seq processing and the association analysis described in the Results and in Text S1.
(TXT)

## References

[1] Sarpal AS, Kapur GS, Mukherjee S, Tiwari AK (2001). PONA analyses of cracked gasoline by 1H NMR spectroscopy. Part II.. Fuel.

[2] Murahashi S (1938). \Über die riechstoffe des matsutake (Armillaria Matsutake Ito et Imai Agaricaceae).. Sci Pap Inst Phys Chem Res(Tokyo).

[3] Combet E, Henderson J, Eastwood DC, Burton KS (2006). Eight-carbon volatiles in mushrooms and fungi: properties, analysis, and biosynthesis.. Mycoscience.

[4] Strobel GA, Knighton B, Kluck K, Ren Y, Livinghouse T (2008). The production of myco-diesel hydrocarbons and their derivatives by the endophytic fungus Gliocladium roseum (NRRL 50072).. Microbiology.

[5] Griffin MA, Spakowicz DJ, Gianoulis TA, Strobel SA (2010). Volatile organic compound production by organisms in the Ascocoryne genus and a reevaluation of myco-diesel production by NRRL 50072.. Microbiology.

[6] Strobel G, Tomsheck A, Geary B, Spakowicz D, Strobel S (2010). Endophyte Strain NRRL - 50072 producing volatile organics is a species of Ascocoryne.. Mycology: An International Journal on Fungal Biology.

[7] Strobel GA, Knighton B, Kluck K, Ren Y, Livinghouse T (2010). The production of myco-diesel hydrocarbons and their derivatives by the endophytic fungus Gliocladium roseum (NRRL 50072).. Microbiology.

[8] Fortman JL, Chhabra S, Mukhopadhyay A, Chou H, Lee TS (2008). Biofuel alternatives to ethanol: pumping the microbial well.. Trends Biotechnol.

[9] Beller HR, Goh E-B, Keasling JD (2010). Genes Involved in Long-Chain Alkene Biosynthesis in Micrococcus luteus.. Appl Environ Microbiol.

[10] Sukovich DJ, Seffernick JL, Richman JE, Hunt KA, Gralnick JA (2010). Structure, function, and insights into the biosynthesis of a head-to-head hydrocarbon in Shewanella oneidensis strain MR-1.. Appl Environ Microbiol.

[11] Schirmer A, Rude MA, Li X, Popova E, del Cardayre SB (2010). Microbial Biosynthesis of Alkanes.. Science.

[12] Rude MA, Baron TS, Brubaker S, Alibhai M, Del Cardayre SB (2011). Terminal Olefin (1-Alkene) Biosynthesis by a Novel P450 Fatty Acid Decarboxylase from Jeotgalicoccus Species.. Appl Environ Microbiol.

[13] Tressl R, Bahri D, Engel KH (1981). Lipid oxidation in fruits and vegetables..

[14] Tressl R, Bahri D, Engel KH (1982). Formation of eight-carbon and ten-carbon components in mushrooms (Agaricus campestris).. Journal of Agricultural and Food Chemistry.

[15] Wurzenberger M, Grosch W (1984). The formation of 1-octen-3-ol from the 10-hydroperoxide isomer of linoleic acid by a hydroperoxide lyase in mushrooms (Psalliota bispora).. Biochimica et Biophysica Acta (BBA)-Lipids and Lipid Metabolism.

[16] Wurzenberger M, Grosch W (1984). Stereochemistry of the cleavage of the 10-hydroperoxide isomer of linoleic acid to 1-octen-3-ol by a hydroperoxide lyase from mushrooms (psalliota bispora).. Biochimica et Biophysica Acta (BBA) - Lipids and Lipid Metabolism.

[17] Brodhun F, Schneider S, Göbel C, Hornung E, Feussner I (2010). PpoC from *Aspergillus nidulans* is a fusion protein with only one active haem.. Biochem J.

[18] Askenazi M, Driggers EM, Holtzman DA, Norman TC, Iverson S (2003). Integrating transcriptional and metabolite profiles to direct the engineering of lovastatin-producing fungal strains.. Nat Biotech.

[19] Bradley PH, Brauer MJ, Rabinowitz JD, Troyanskaya OG (2009). Coordinated concentration changes of transcripts and metabolites in Saccharomyces cerevisiae.. PLoS Comput Biol.

[20] Redestig H, Costa IG (2011). Detection and interpretation of metabolite–transcript coresponses using combined profiling data.. Bioinformatics.

[21] Hirai MY, Yano M, Goodenowe DB, Kanaya S, Kimura T (2004). Integration of transcriptomics and metabolomics for understanding of global responses to nutritional stresses in Arabidopsis thaliana.. Proceedings of the National Academy of Sciences of the United States of America.

[22] Hancock T, Takigawa I, Mamitsuka H (2010). Mining metabolic pathways through gene expression.. Bioinformatics.

[23] Saito N, Ohashi Y, Soga T, Tomita M (2010). Unveiling cellular biochemical reactions via metabolomics-driven approaches.. Curr Opin Microbiol.

[24] Margulies M, Egholm M, Altman WE, Attiya S, Bader JS (2005). Genome sequencing in microfabricated high-density picolitre reactors.. Nature.

[25] Fitzpatrick D, Logue M, Stajich J, Butler G (2006). A fungal phylogeny based on 42 complete genomes derived from supertree and combined gene analysis.. BMC Evolutionary Biology.

[26] Parra G, Bradnam K, Korf I (2007). CEGMA: a pipeline to accurately annotate core genes in eukaryotic genomes.. Bioinformatics.

[27] Benson DA, Karsch-Mizrachi I, Lipman DJ, Ostell J, Wheeler DL (2007). GenBank.. Nucleic Acids Research.

[28] Nagalakshmi U, Wang Z, Waern K, Shou C, Raha D (2008). The Transcriptional Landscape of the Yeast Genome Defined by RNA Sequencing.. Science.

[29] Mortazavi A, Williams BA, McCue K, Schaeffer L, Wold B (2008). Mapping and quantifying mammalian transcriptomes by RNA-Seq.. Nat Meth.

[30] Supelco Bulletin 869A (n.d.). Solid Phase Microextraction: Solventless Sample Preparation for Monitoring Flavor Compounds by Capillary Gas Chromatography.. http://www.sigmaaldrich.com/etc/medialib/docs/Supelco//4524.pdf.

[31] Ozsolak F, Kapranov P, Foissac S, Kim SW, Fishilevich E (2010). Comprehensive Polyadenylation Site Maps in Yeast and Human Reveal Pervasive Alternative Polyadenylation.. Cell.

[32] Bumgarner SL, Dowell RD, Grisafi P, Gifford DK, Fink GR (2009). Toggle involving cis-interfering noncoding RNAs controls variegated gene expression in yeast.. Proceedings of the National Academy of Sciences.

[33] Gerstein MB, Lu ZJ, Van Nostrand EL, Cheng C, Arshinoff BI (2010). Integrative Analysis of the Caenorhabditis elegans Genome by the modENCODE Project.. Science.

[34] Cantarel BL, Coutinho PM, Rancurel C, Bernard T, Lombard V (2009). The Carbohydrate-Active EnZymes database (CAZy): an expert resource for Glycogenomics.. Nucleic Acids Res.

[35] Saloheimo M, Paloheimo M, Hakola S, Pere J, Swanson B (2002). Swollenin, a Trichoderma reesei protein with sequence similarity to the plant expansins, exhibits disruption activity on cellulosic materials.. European Journal of Biochemistry.

[36] Chen X-ai, Ishida N, Todaka N, Nakamura R, Maruyama J-ichi (2010). Promotion of Efficient Saccharification of Crystalline Cellulose by Aspergillus fumigatus Swo1.. Appl Environ Microbiol.

[37] Fischer CR, Klein-Marcuschamer D, Stephanopoulos G (2008). Selection and optimization of microbial hosts for biofuels production.. Metabolic Engineering.

[38] James EG, Sarah EC, Christina Cuomo LJ, Jennifer RW, Serafim Batzoglou SI (2005). Sequencing of Aspergillus nidulans and comparative analysis with A. fumigatus and A. oryzae.. Nature.

[39] Martinez D, Berka RM, Henrissat B, Saloheimo M, Arvas M (2008). Genome sequencing and analysis of the biomass-degrading fungus Trichoderma reesei (syn. Hypocrea jecorina).. Nature biotechnology.

[40] Camera SL, Balagué C, Göbel C, Geoffroy P, Legrand M (2009). The *Arabidopsis* Patatin-Like Protein 2 (PLP2) Plays an Essential Role in Cell Death Execution and Differentially Affects Biosynthesis of Oxylipins and Resistance to Pathogens.. MPMI.

[41] Del Sorbo G, Schoonbeek H, De Waard MA (2000). Fungal transporters involved in efflux of natural toxic compounds and fungicides.. Fungal Genet Biol.

[42] Yadav G, Gokhale RS, Mohanty D (2009). Towards Prediction of Metabolic Products of Polyketide Synthases: An In Silico Analysis.. PLoS Comput Biol.

[43] Bouhired S, Weber M, Kempf-Sontag A, Keller NP, Hoffmeister D (2007). Accurate prediction of the Aspergillus nidulans terrequinone gene cluster boundaries using the transcriptional regulator LaeA.. Fungal Genetics and Biology.

[44] Yadav G, Gokhale RS, Mohanty D (2003). SEARCHPKS: a program for detection and analysis of polyketide synthase domains.. Nucleic Acids Research.

[45] Piel J (2010). Biosynthesis of polyketides by trans-AT polyketide synthases.. Nat Prod Rep.

[46] Koonin EV, Wolf YI (2008). Genomics of bacteria and archaea: the emerging dynamic view of the prokaryotic world.. Nucleic Acids Research.

[47] Pellegrini M, Marcotte EM, Thompson MJ, Eisenberg D, Yeates TO (1999). Assigning protein functions by comparative genome analysis: Protein phylogenetic profiles.. Proc Natl Acad Sci U S A.

[48] Korbel JO, Jensen LJ, von Mering C, Bork P (2004). Analysis of genomic context: prediction of functional associations from conserved bidirectionally transcribed gene pairs.. Nat Biotech.

[49] Marcotte EM, Pellegrini M, Ng H-L, Rice DW, Yeates TO (1999). Detecting Protein Function and Protein-Protein Interactions from Genome Sequences.. Science.

[50] Overbeek R, Fonstein M, D'Souza M, Pusch GD, Maltsev N (1999). The use of gene clusters to infer functional coupling.. Proceedings of the National Academy of Sciences.

[51] Kumar CG, Everts RE, Loor JJ, Lewin HA (2010). Functional annotation of novel lineage-specific genes using co-expression and promoter analysis.. BMC Genomics.

[52] Stephanopoulos G (2007). Challenges in Engineering Microbes for Biofuels Production.. Science.

[53] Lee SK, Chou H, Ham TS, Lee TS, Keasling JD (2008). Metabolic engineering of microorganisms for biofuels production: from bugs to synthetic biology to fuels.. Current Opinion in Biotechnology.

[54] Andreou A, Brodhun F, Feussner I (2009). Biosynthesis of oxylipins in non-mammals.. Progress in Lipid Research.

[55] Grechkin AN, Hamberg M (2004). The “heterolytic hydroperoxide lyase” is an isomerase producing a short-lived fatty acid hemiacetal.. Biochimica et Biophysica Acta (BBA) - Molecular and Cell Biology of Lipids.

[56] Berdy J (2005). Bioactive Microbial Metabolites.. J Antibiot.

[57] Dodds DR, Gross RA (2007). Chemicals from Biomass.. Science.

[58] Croes D, Couche F, Wodak SJ, van Helden J (2005). Metabolic PathFinding: inferring relevant pathways in biochemical networks.. Nucleic Acids Research.

[59] Gao J, Ellis LBM, Wackett LP (2010). The University of Minnesota Biocatalysis/Biodegradation Database: improving public access.. Nucleic Acids Res.

[60] Moriya Y, Shigemizu D, Hattori M, Tokimatsu T, Kotera M (2010). PathPred: an enzyme-catalyzed metabolic pathway prediction server.. Nucleic Acids Res.

[61] Rahman SA, Advani P, Schunk R, Schrader R, Schomburg D (2005). Metabolic pathway analysis web service (Pathway Hunter Tool at CUBIC).. Bioinformatics.

[62] Caspi R, Foerster H, Fulcher CA, Kaipa P, Krummenacker M (2008). The MetaCyc Database of metabolic pathways and enzymes and the BioCyc collection of Pathway/Genome Databases.. Nucleic Acids Res.

[63] Wink M (1988). Plant breeding: importance of plant secondary metabolites for protection against pathogens and herbivores.. Theoret Appl Genetics.

[64] Wieloch W (2006). Chromosome visualisation in filamentous fungi.. J Microbiol Methods.

[65] Griffin MA, Spakowicz DJ, Gianoulis TA, Strobel SA (2010). Volatile organic compound production by organisms in the genus Ascocoryne and a re-evaluation of myco-diesel production by NRRL 50072.. Microbiology.

[66] Altschul SF, Gish W, Miller W, Myers EW, Lipman DJ (1990). Basic local alignment search tool.. J Mol Biol.

[67] Cantarel BL, Coutinho PM, Rancurel C, Bernard T, Lombard V (2009). The Carbohydrate-Active EnZymes database (CAZy): an expert resource for Glycogenomics.. Nucleic Acids Res.

[68] Eddy SR (2009). A new generation of homology search tools based on probabilistic inference.. Genome Inform.

[69] Alam I, Hubbard S, Oliver S, Rattray M (2007). A kingdom-specific protein domain HMM library for improved annotation of fungal genomes.. BMC Genomics.

[70] Finn RD, Tate J, Mistry J, Coggill PC, Sammut SJ (2007). The Pfam protein families database.. Nucleic Acids Research.

[71] Kanehisa M, Araki M, Goto S, Hattori M, Hirakawa M (2008). KEGG for linking genomes to life and the environment.. Nucleic Acids Res.

[72] Moriya Y, Itoh M, Okuda S, Yoshizawa AC, Kanehisa M (2007). KAAS: an automatic genome annotation and pathway reconstruction server.. Nucleic Acids Res.

[73] Ashburner M, Ball CA, Blake JA, Botstein D, Butler H (2000). Gene ontology: tool for the unification of biology. The Gene Ontology Consortium.. Nat Genet.

[74] Arnaud MB, Chibucos MC, Costanzo MC, Crabtree J, Inglis DO (2010). The Aspergillus Genome Database, a curated comparative genomics resource for gene, protein and sequence information for the Aspergillus research community.. Nucleic Acids Res.

[75] Langmead B, Trapnell C, Pop M, Salzberg SL (2009). Ultrafast and memory-efficient alignment of short DNA sequences to the human genome.. Genome Biol.

[76] Habegger L, Sboner A, Gianoulis TA, Rozowsky J, Agarwal A (2010). RSEQtools: A modular framework to analyze RNA-Seq data using compact, anonymized data summaries.. http://bioinformatics.oxfordjournals.org/content/early/2010/12/05/bioinformatics.btq643.abstract.

[77] Kent WJ (2002). BLAT–the BLAST-like alignment tool.. Genome Res.

