# Supplementary figures and images for: Genomic Analysis of the Hydrocarbon-Producing, Cellulolytic, Endophytic Fungus Ascocoryne sarcoides
Source: PLoS Genet. 2012 Mar 1;8(3):e1002558. doi: 10.1371/journal.pgen.1002558 (PMC3291568; doi:10.1371/journal.pgen.1002558)

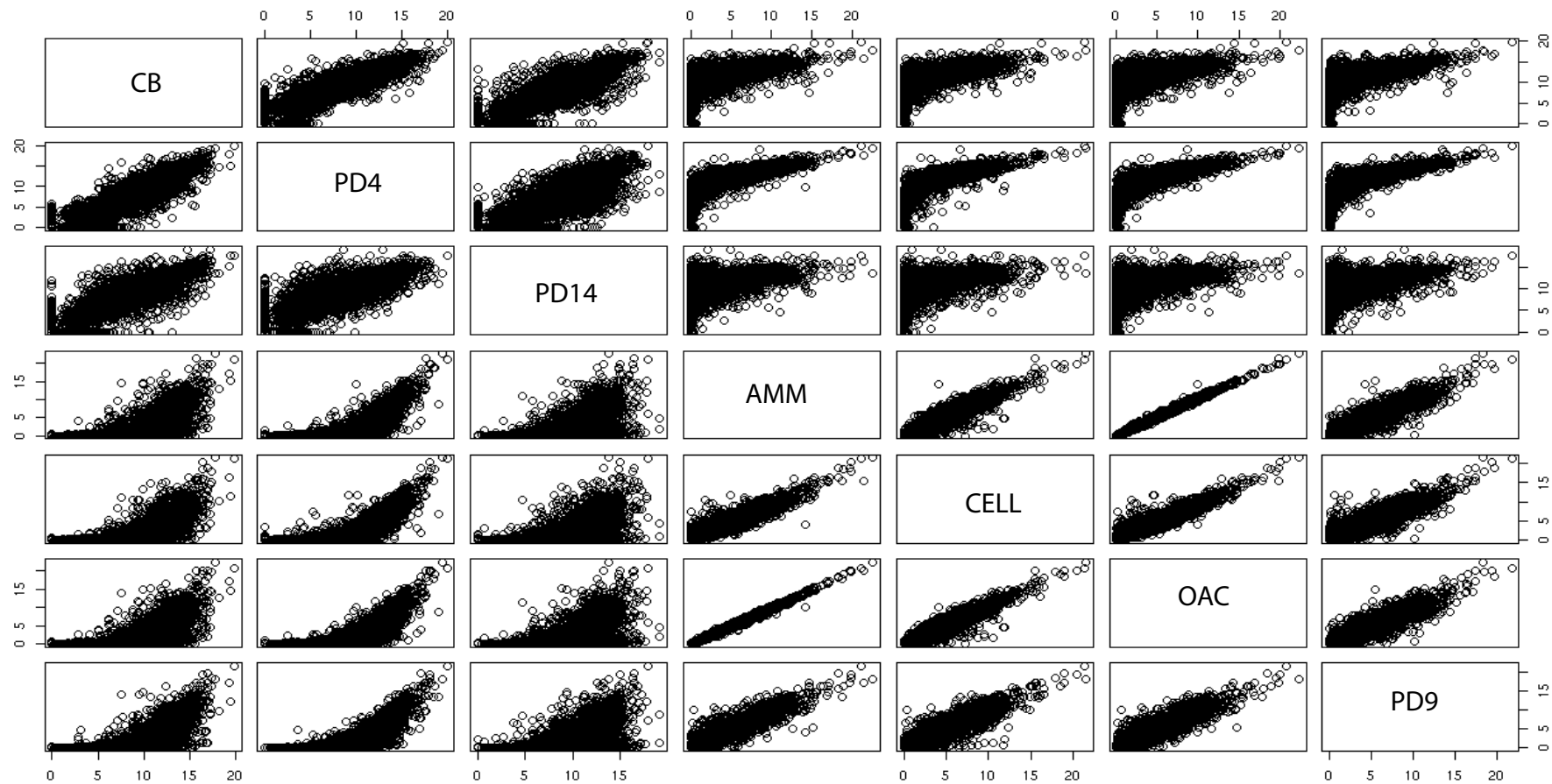

Supplement: Figure S1 — All versus All comparison of transcriptional profiles for Illumina runs in all seven different growth conditions. Axes are log2 RPKM values. (PDF) [file pgen.1002558.s001.pdf]

**Cellulose Lane1 vs Lane2**

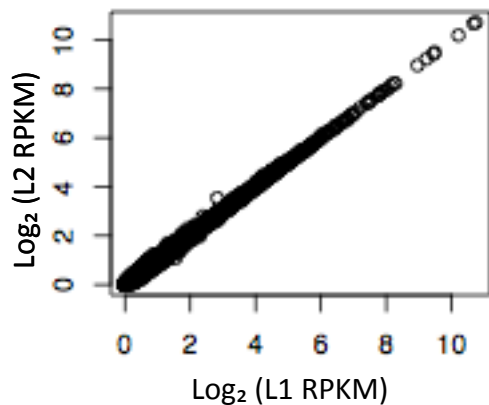

**OAC Lane1 vs Lane2**

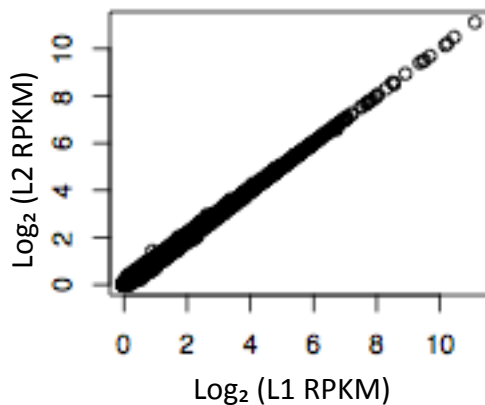

**PD Lane1 vs Lane2**

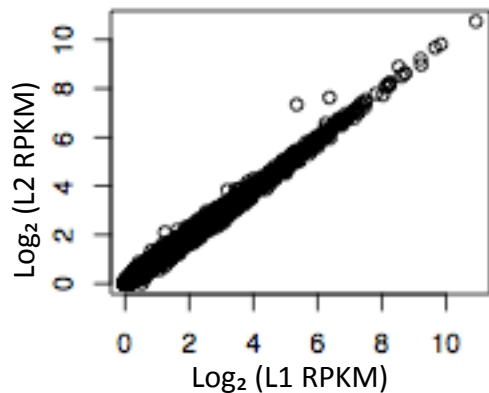

**AMM Lane1 vs Lane2**

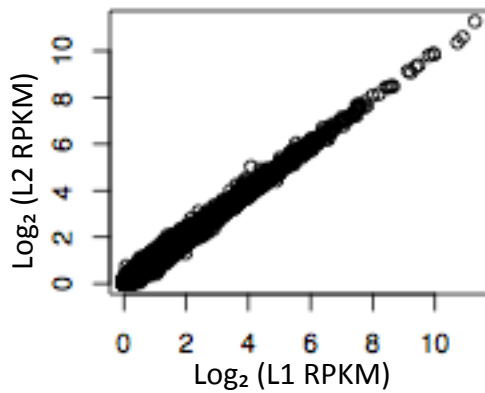

Supplement: Figure S2 — Technical Replicates of Illumina transcription data. There is greater than 99% similarity between the two replicates for each of four conditions. (PDF) [file pgen.1002558.s002.pdf]

# TAR Building: Sensitivity Analysis

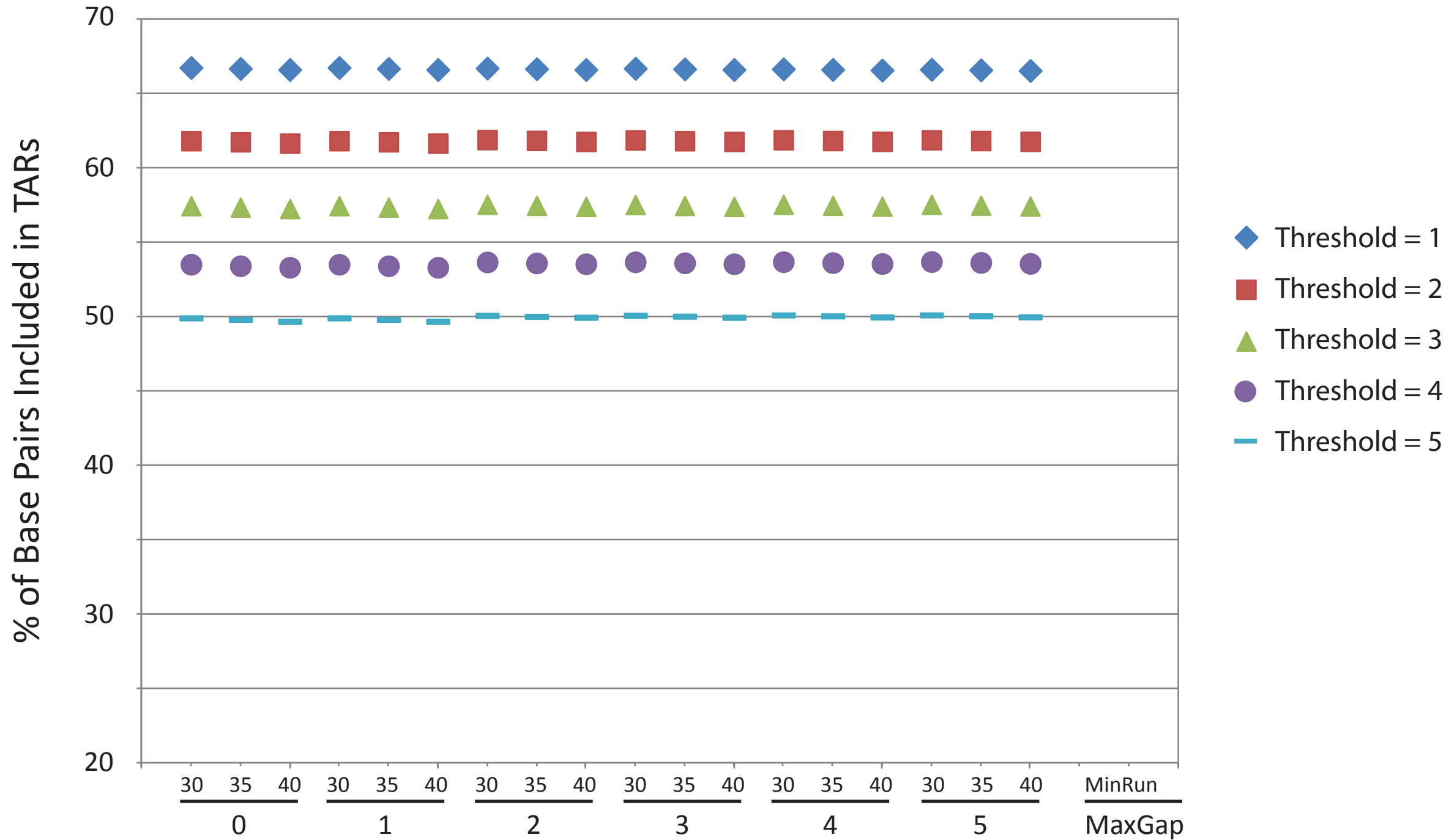

Supplement: Figure S3 — Determining the appropriate parameter values for identifying Transcriptionally Active Regions (TARs). Each line represents a particular threshold in the TAR sensitivity analysis (see legend for Threshold levels). The x-axis is sorted first by the MaxGap value (0,1,2,3,4,and 5) and secondly, for each by the MinRun length value (30, 35, 40). (PDF) [file pgen.1002558.s003.pdf]

# Compound Gene Co-Expression

A

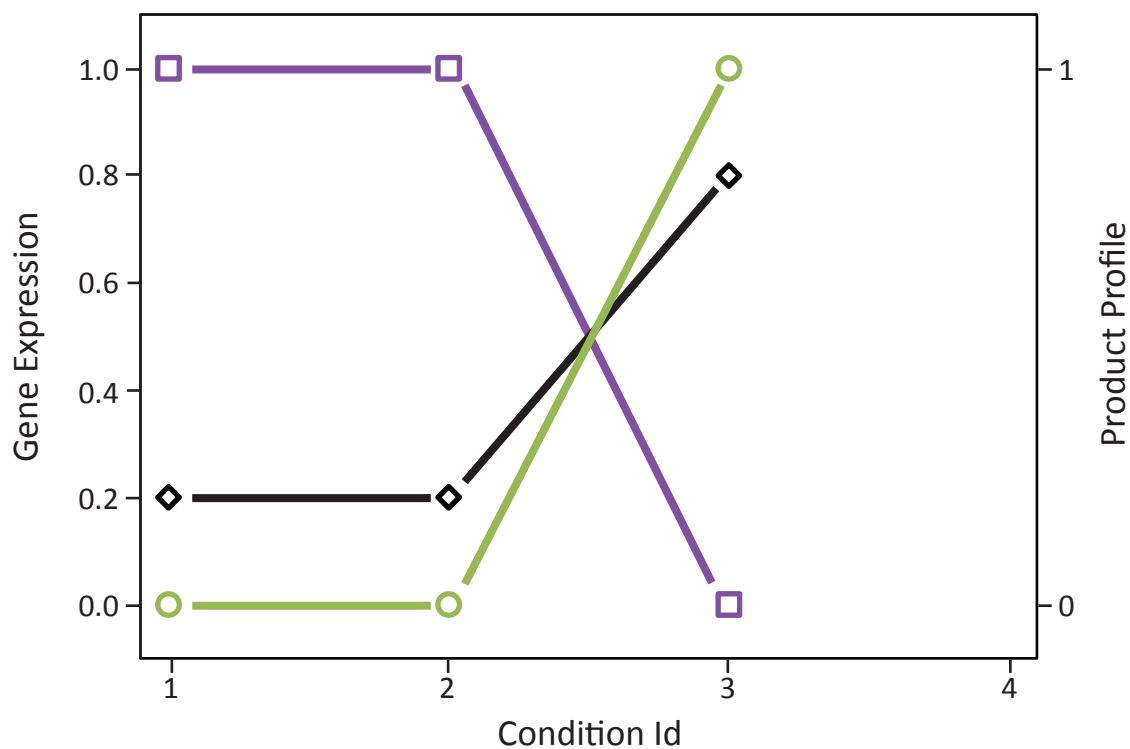

B

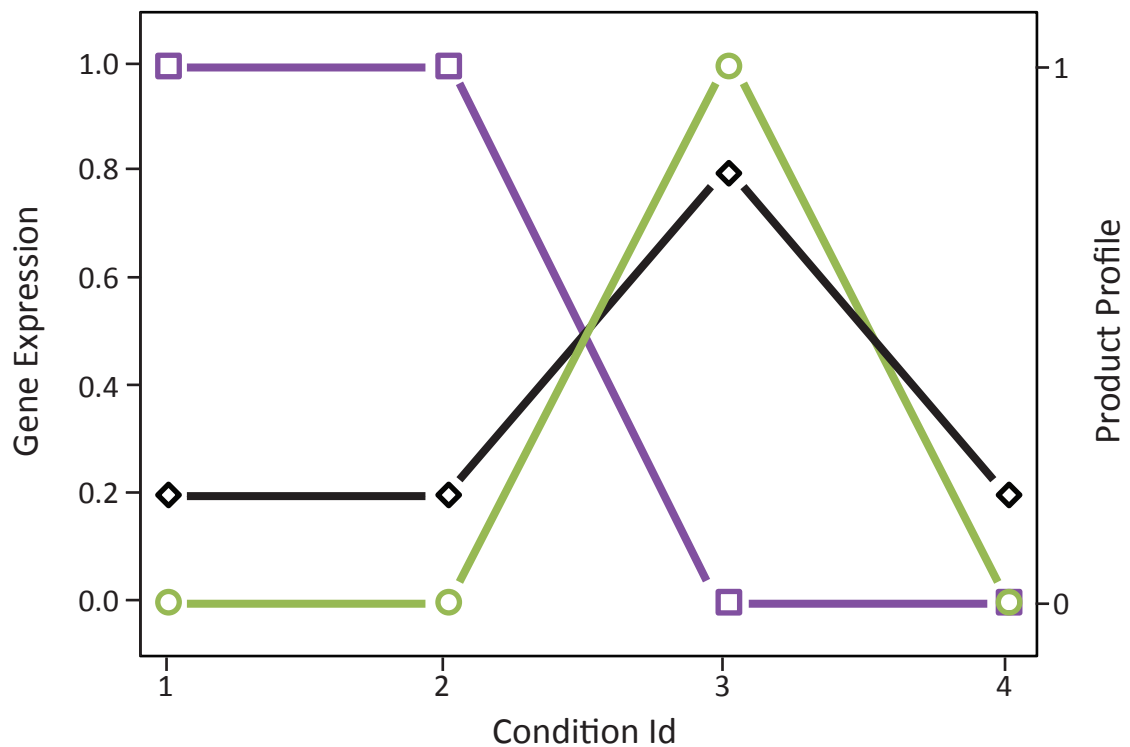

Supplement: Figure S5 — Hypothetical compound gene co-expression profiles demonstrating the need to include null data sets. (A), the purple line and the green line (square and circle, respectively) represent two possible compound profiles (110 and 001). The gene expression of the black line (diamond) is equally correlated with the two compound profiles. (B) A null condition is included in the analysis (condition ID 4). The best compound profile match for the gene's expression pattern (black line) is now the green line (0010). Addition of the null condition permits the distinction between the two compound profiles. See text for more details. (PDF) [file pgen.1002558.s005.pdf]

## Precursor

|     |
|-----|
| 110 |
| 101 |
| 011 |
| 111 |

## Products

|     |     |     |
|-----|-----|-----|
| 100 | 010 | 110 |
| 001 | 100 | 101 |
| 010 | 001 | 011 |
| ANY |     |     |

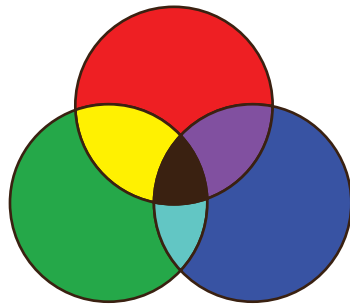

Supplement: Figure S6 — Inferring Product/Reactant Pairs through Compound Profile Consistency. We illustrate this idea with a simple color experiment. As an example, a purple precursor (110) can result in blue (100), red (010), or purple (110) products, but not a yellow (011) product. (PDF) [file pgen.1002558.s006.pdf]

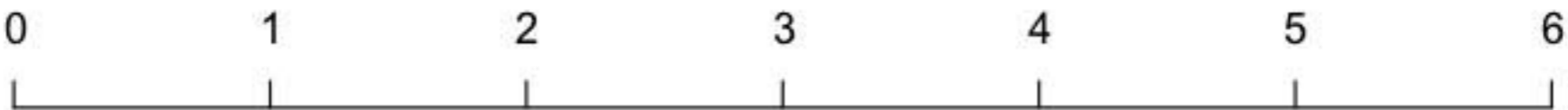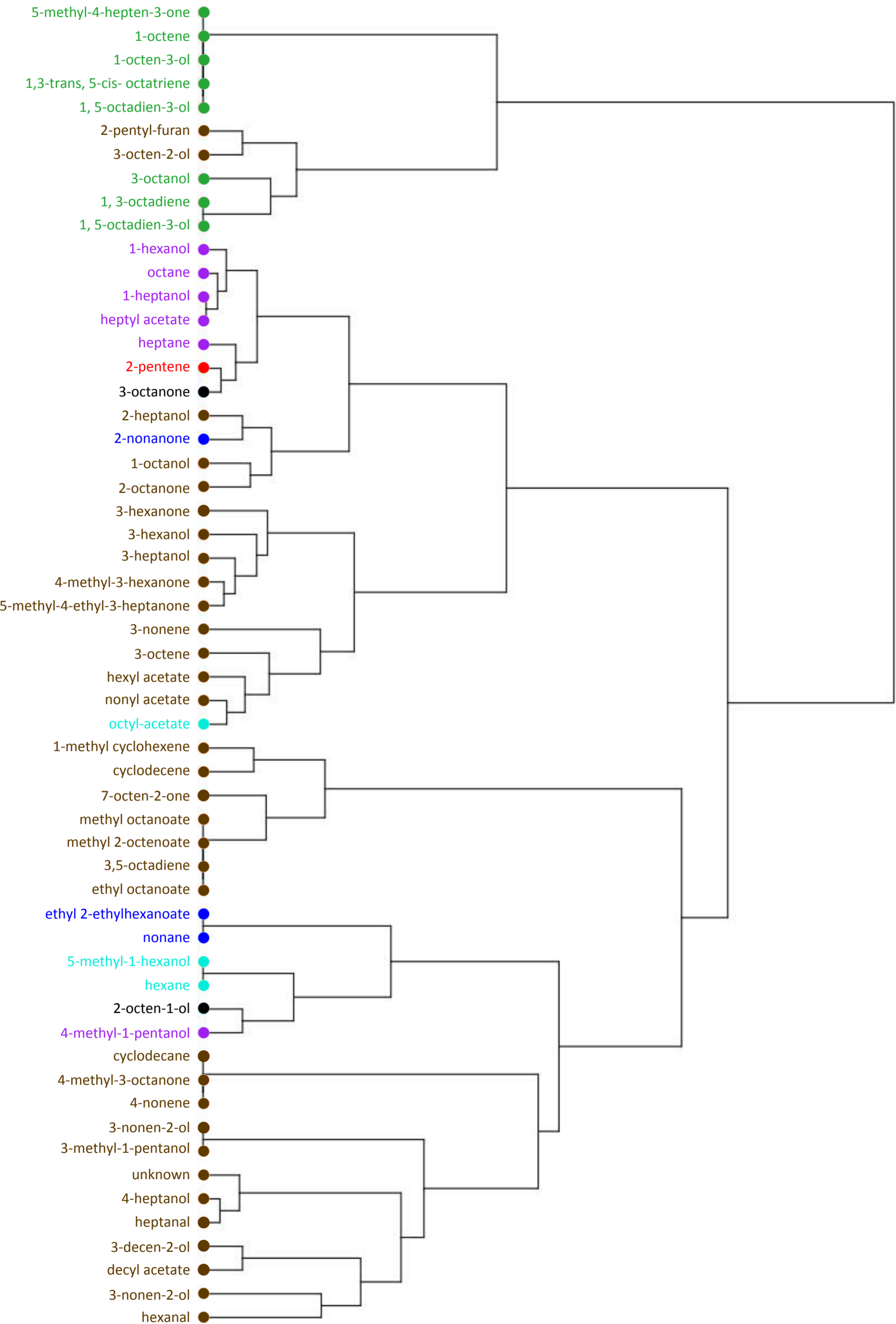

Supplement: Figure S7 — Clustering Compound Co-occurrence. Compounds are clustered based on their co-occurrence as measured by their DLW distance across the whole Ascocoryne genus. Compounds are colored according to their production profiles (using the same color scheme in Figure S6): Green 001, Red 010, Blue 100, Cyan 101, Purple 110 and Black 111. Compounds in Brown were reported in the previous analysis of the Ascocoryne genus for VOC production, but not presently detected in any of the conditions linked with RNA-seq data. (PDF) [file pgen.1002558.s007.pdf]

A.

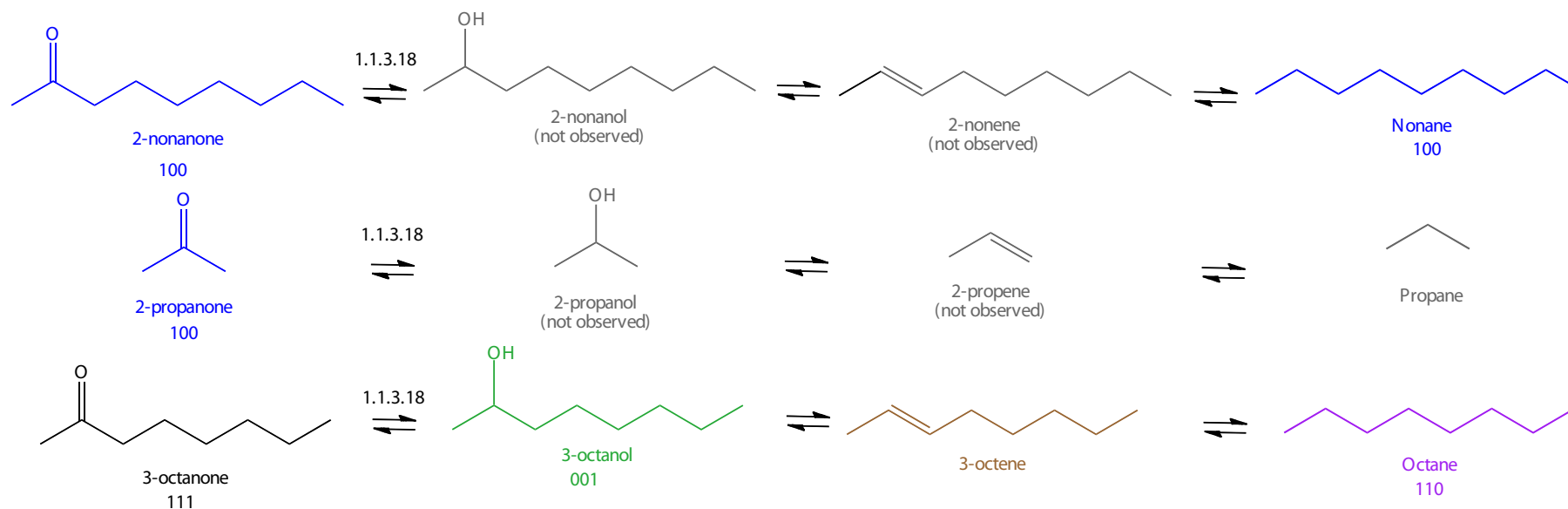

B.

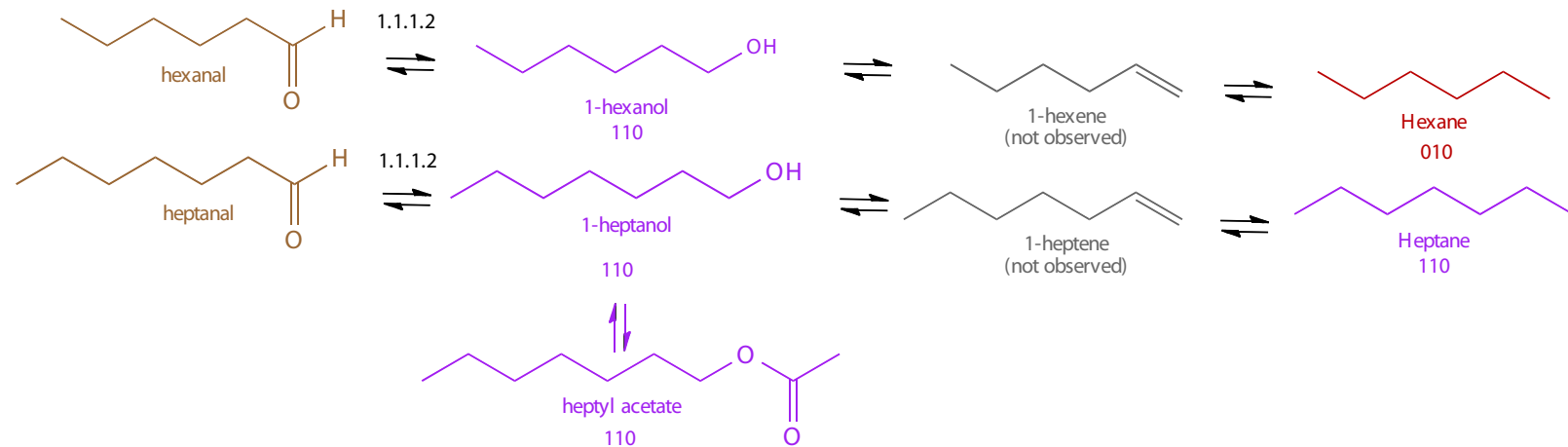

Supplement: Figure S8 — Retrosynthesis of A. sarcoides products. Compounds are colored by their associated profile as defined in Figure S6. Brown indicates those compounds that were previously detected from the Ascocoryne genus, but were not detected during the present the RNA-seq coupled analysis. Gray represents compounds that have never been detected, but are proposed intermediates. (A) Hypothetical schema for producing alkanes from a ketone precursor. (B) Hypothetical schema for converting an aldehyde into the corresponding alkane, as well as possible off-pathway reactions that produce an ester. (PDF) [file pgen.1002558.s008.pdf]

**Cluster 1**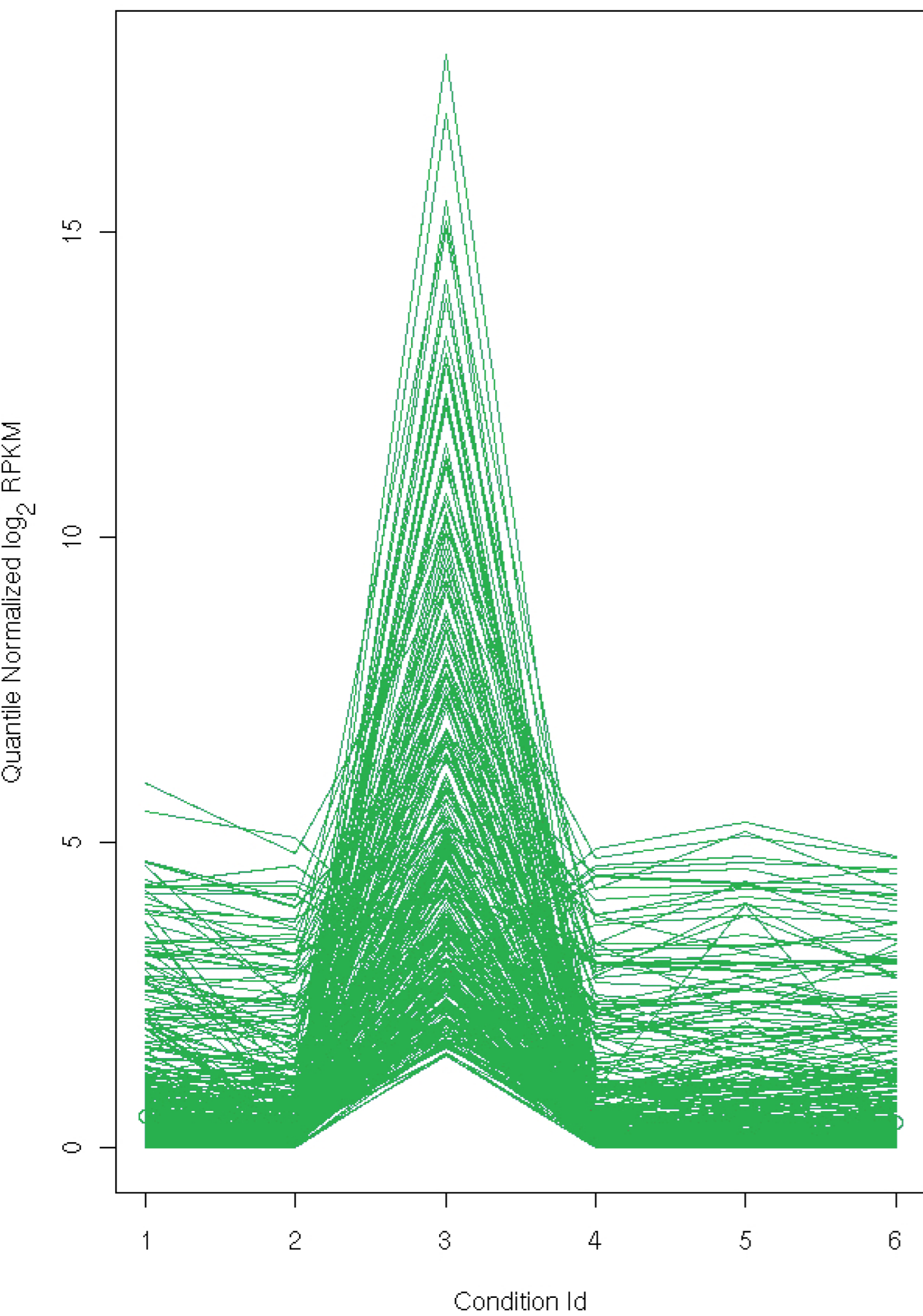**Cluster 2**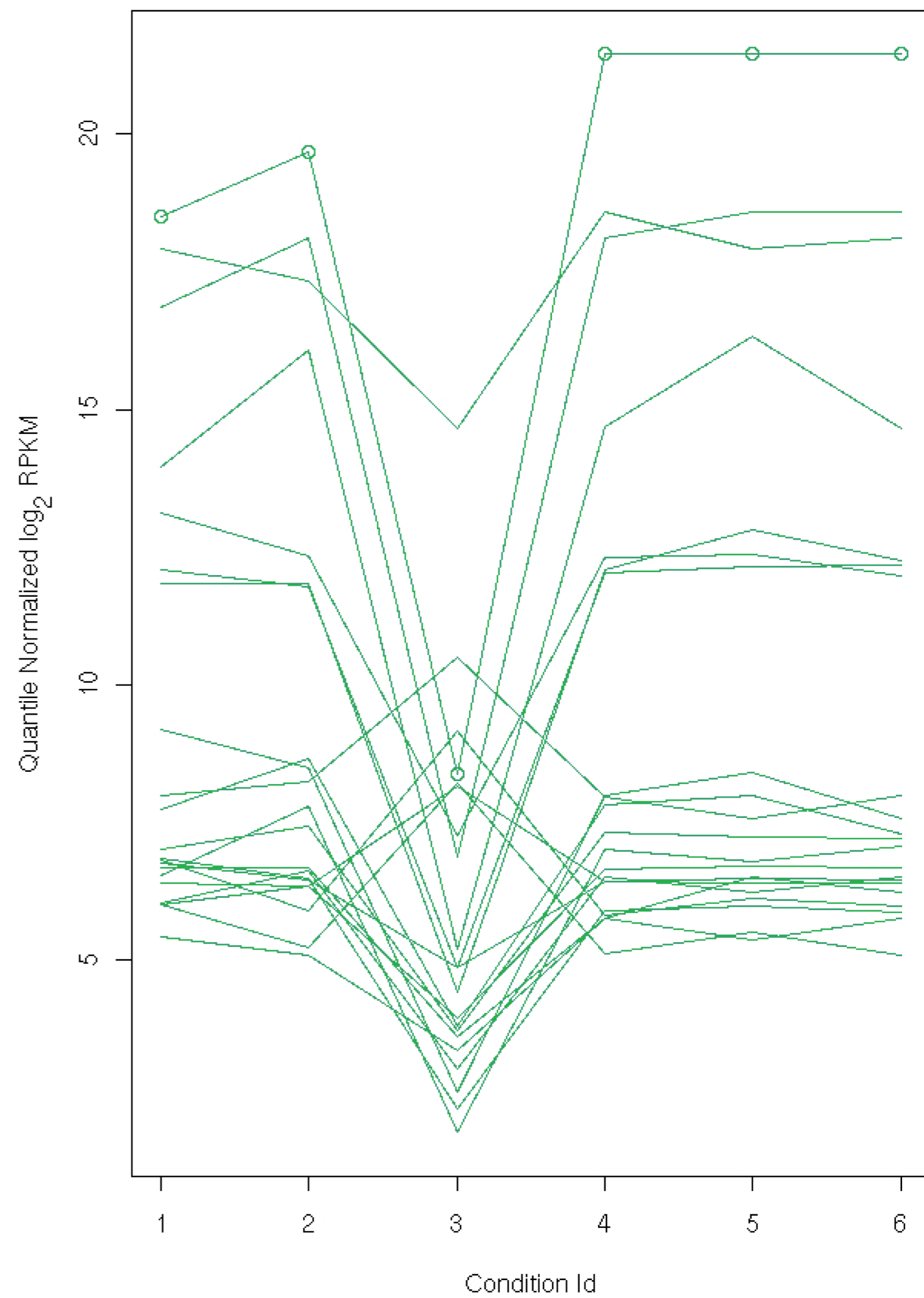

Supplement: Figure S9 — Clustered Gene Co-expression 001. Each of the 3 following figures (Figures S9, S10, S11) was generated as follows. The genes whose expression correlated with a particular compound profile were partitioned using k-means clustering into sets of genes co-expressed across all 6 conditions. Each graph represents the gene expression of a single cluster where the x-axis is the Condition Id and the y-axis is the Quantile Normalized log2 RPKM. The 001 genes partitioned into two clusters, representing up and down regulation states. However, more complex partitioning occurred for the 101 and 111 profiles. (PDF) [file pgen.1002558.s009.pdf]

**Cluster 1**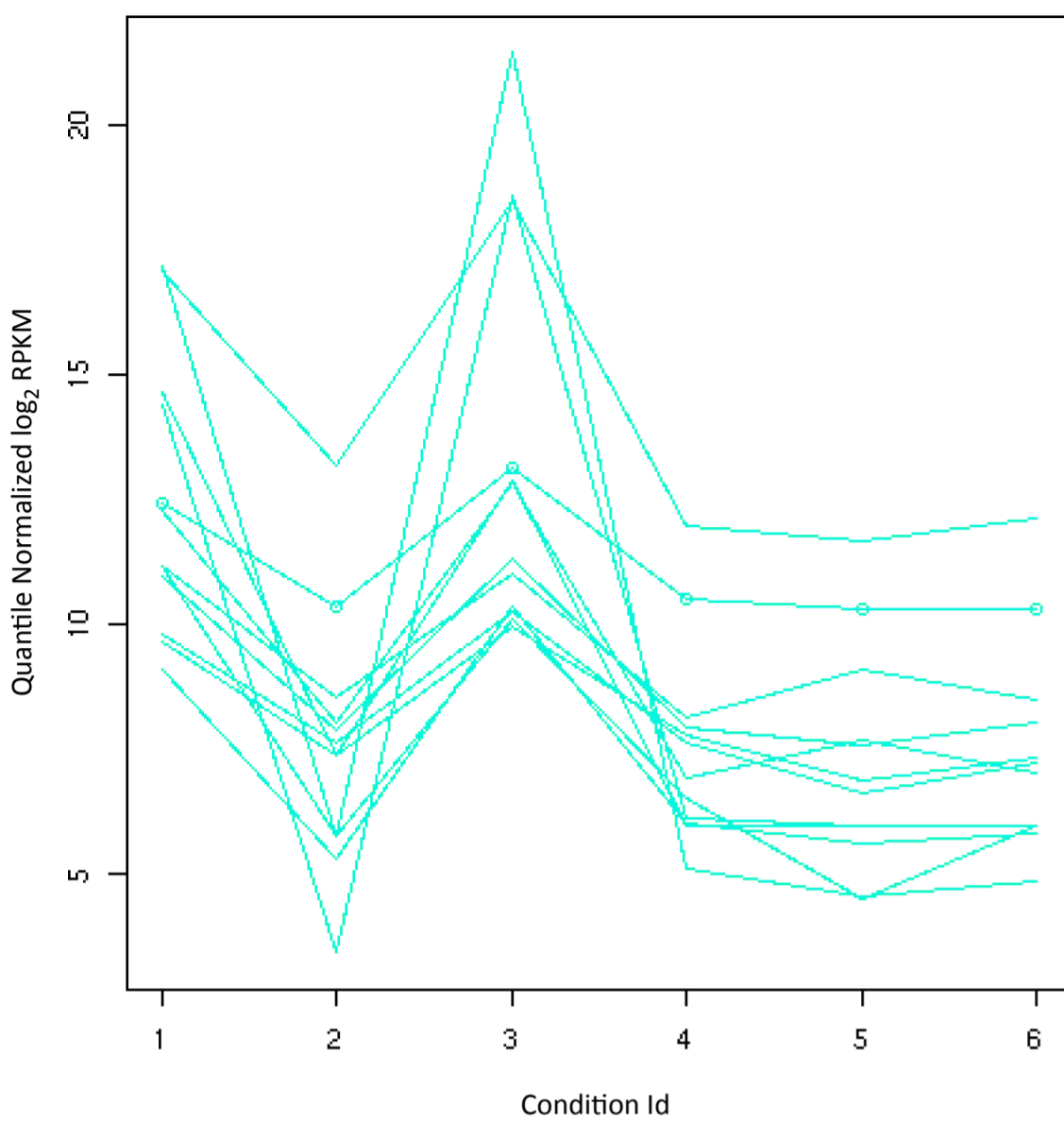**Cluster 2**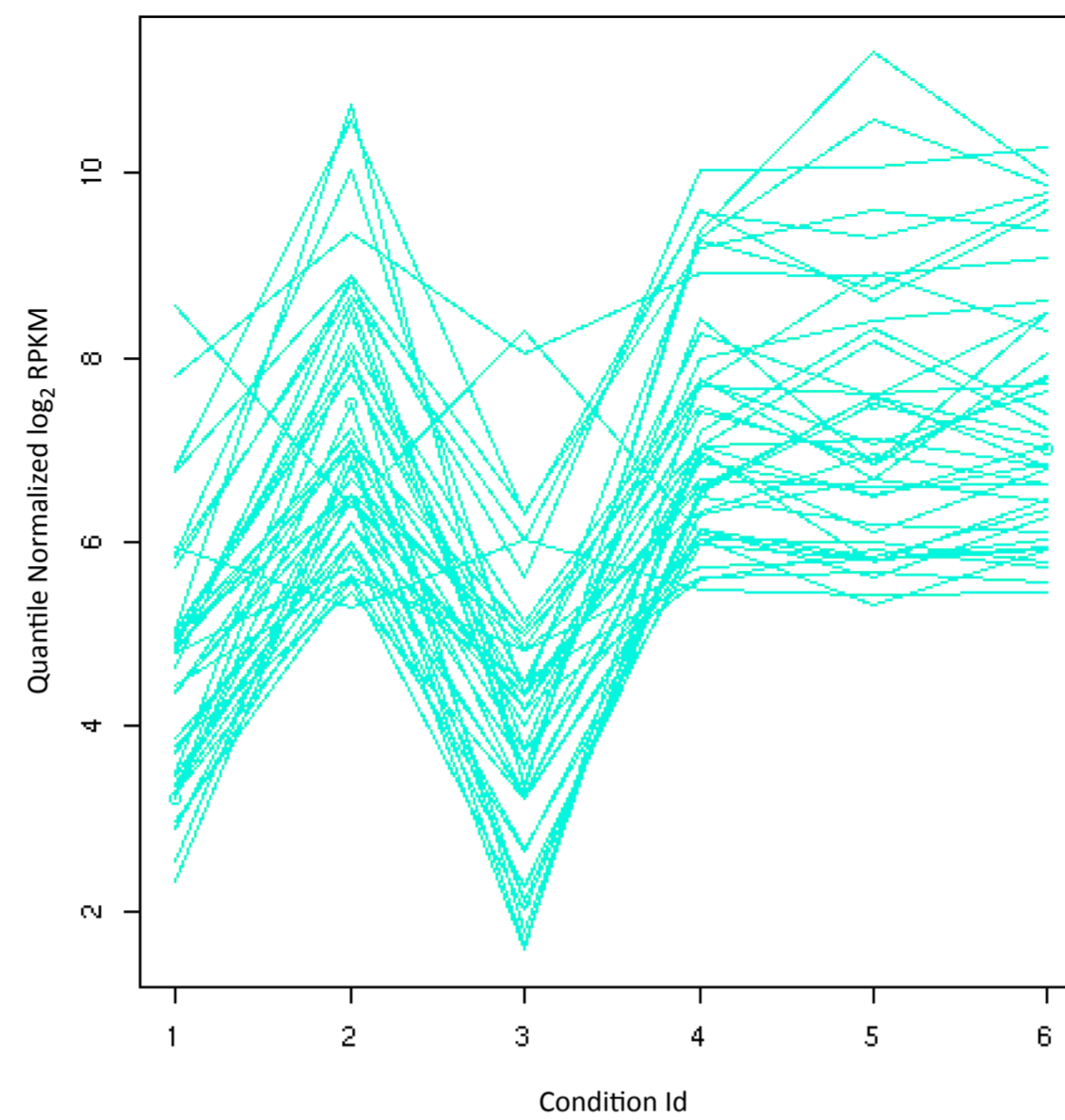**Cluster 3**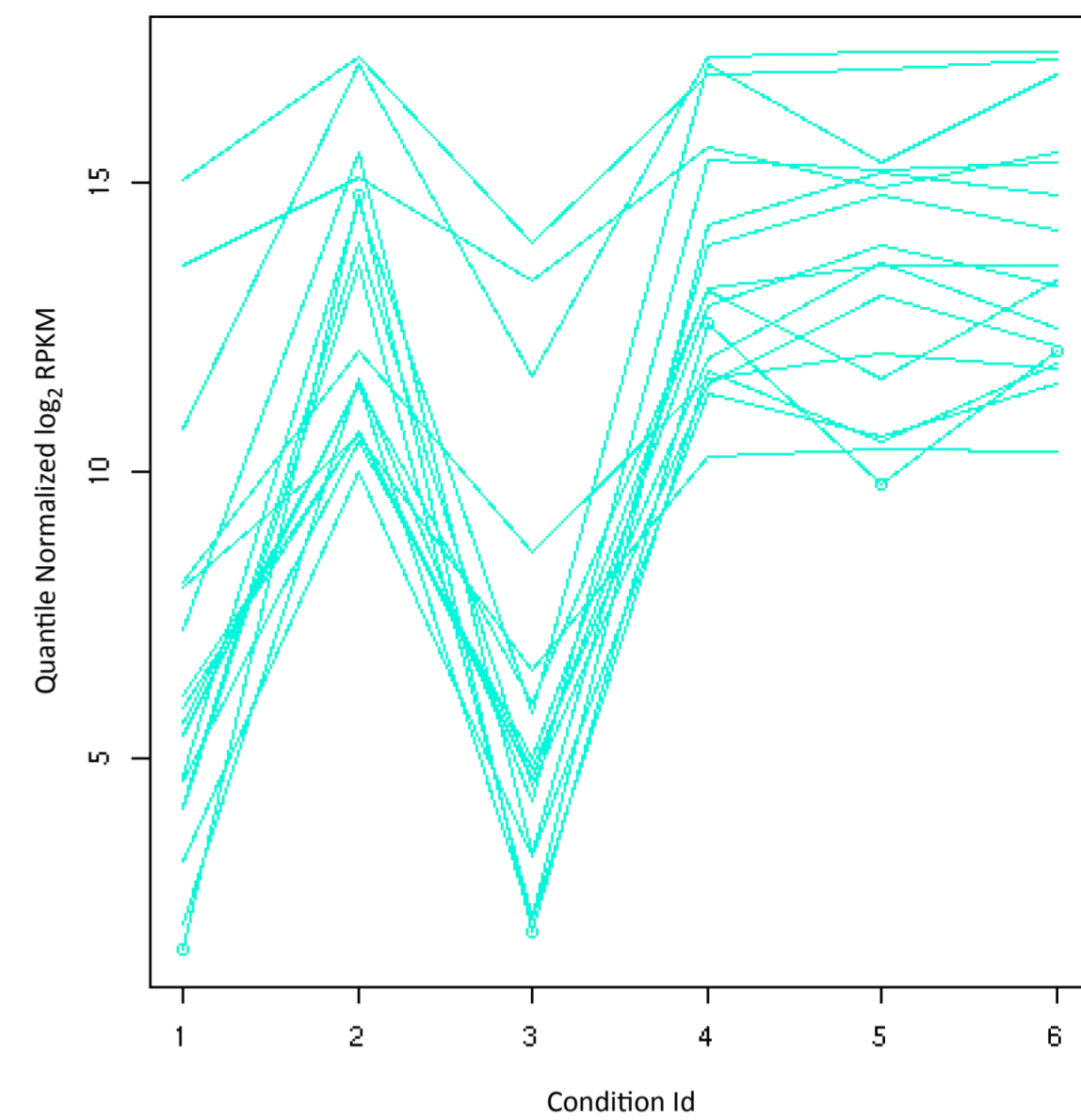**Cluster 4**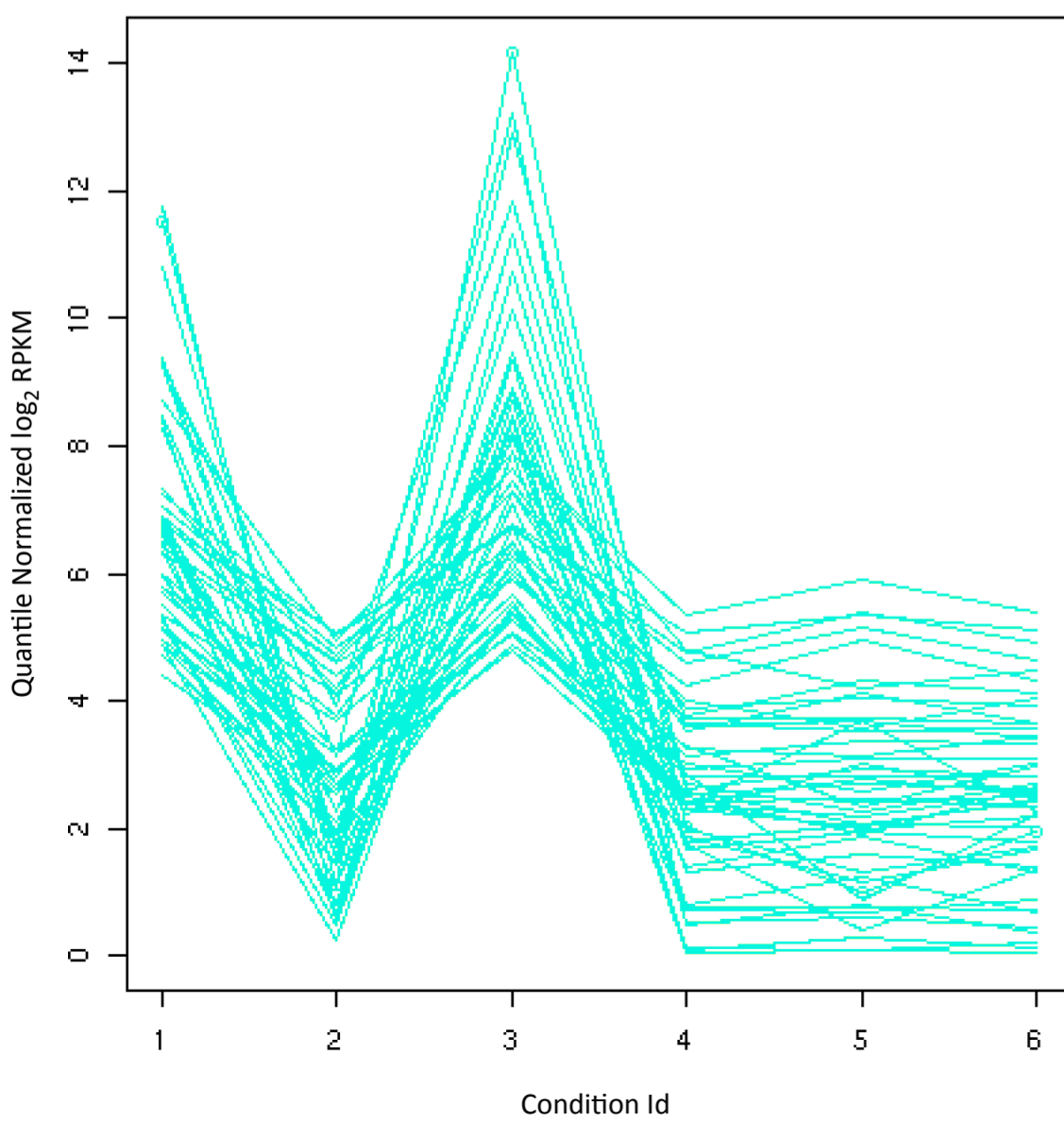**Cluster 5**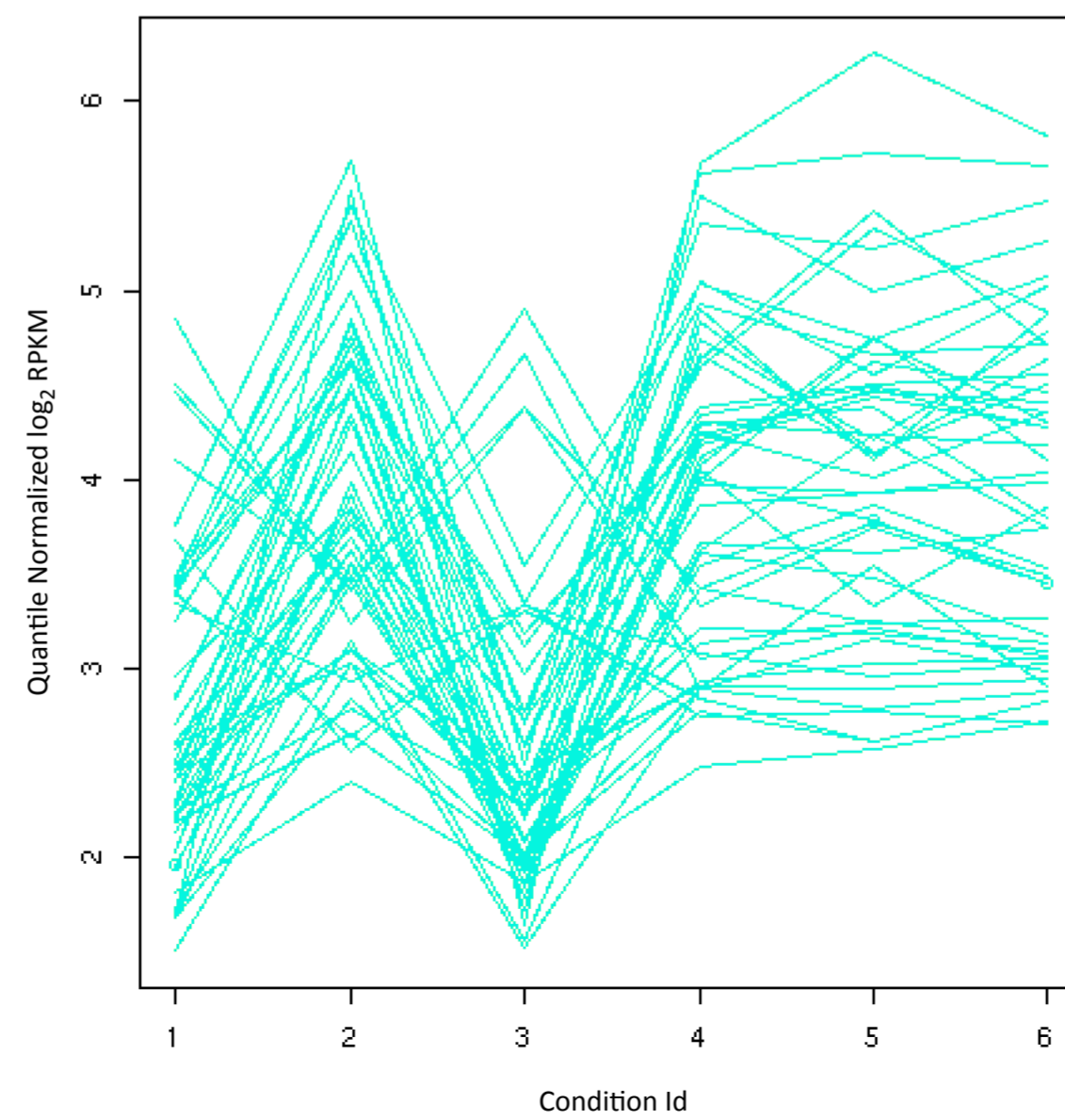**Cluster 6**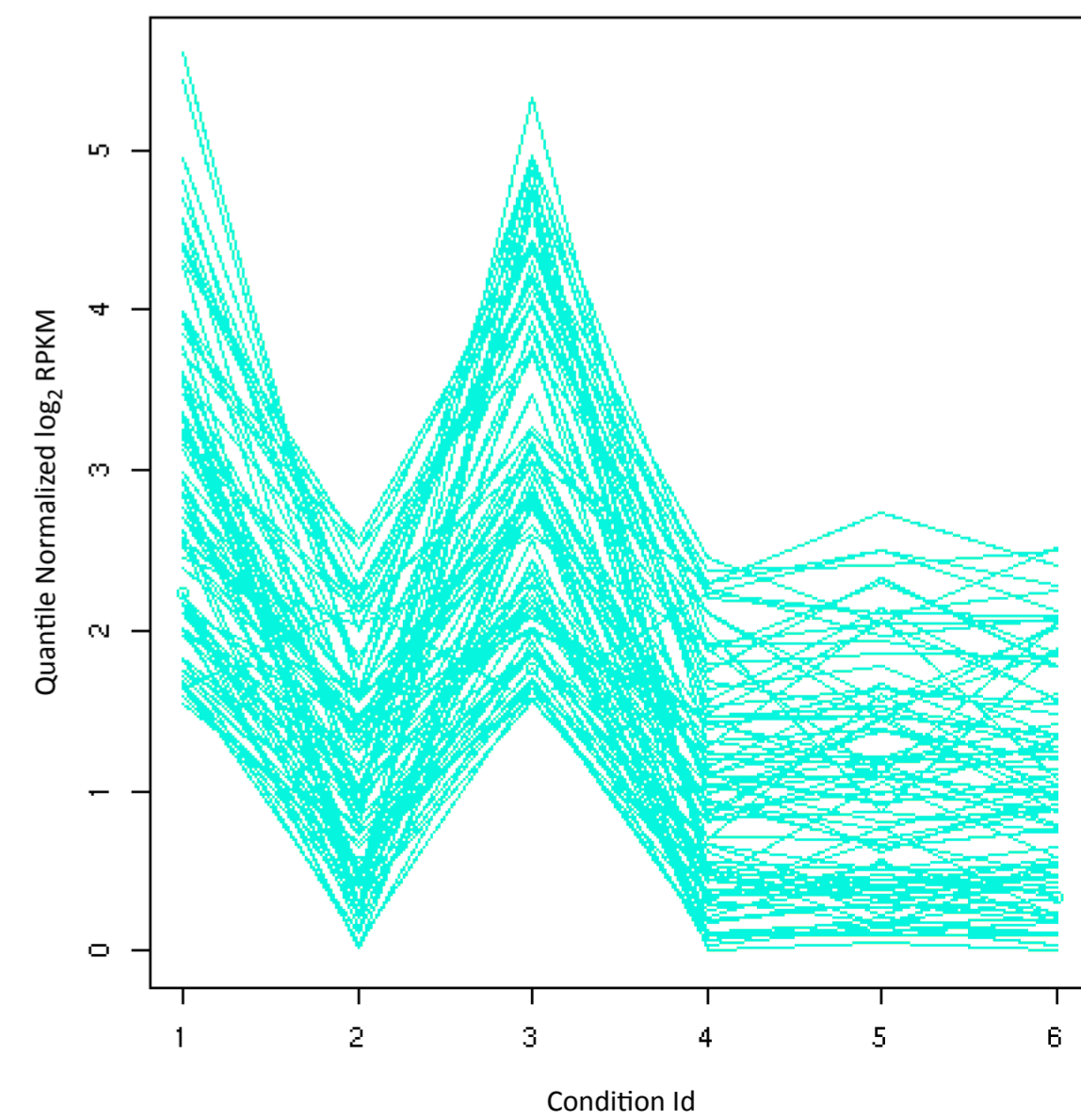

Supplement: Figure S10 — Clustered Gene Co-expression 101. As described for Figure S9, this illustrates the gene expression patterns for all genes correlated with the 101 compound profile, where each plot represents a single cluster of genes. (PDF) [file pgen.1002558.s010.pdf]

Cluster 1

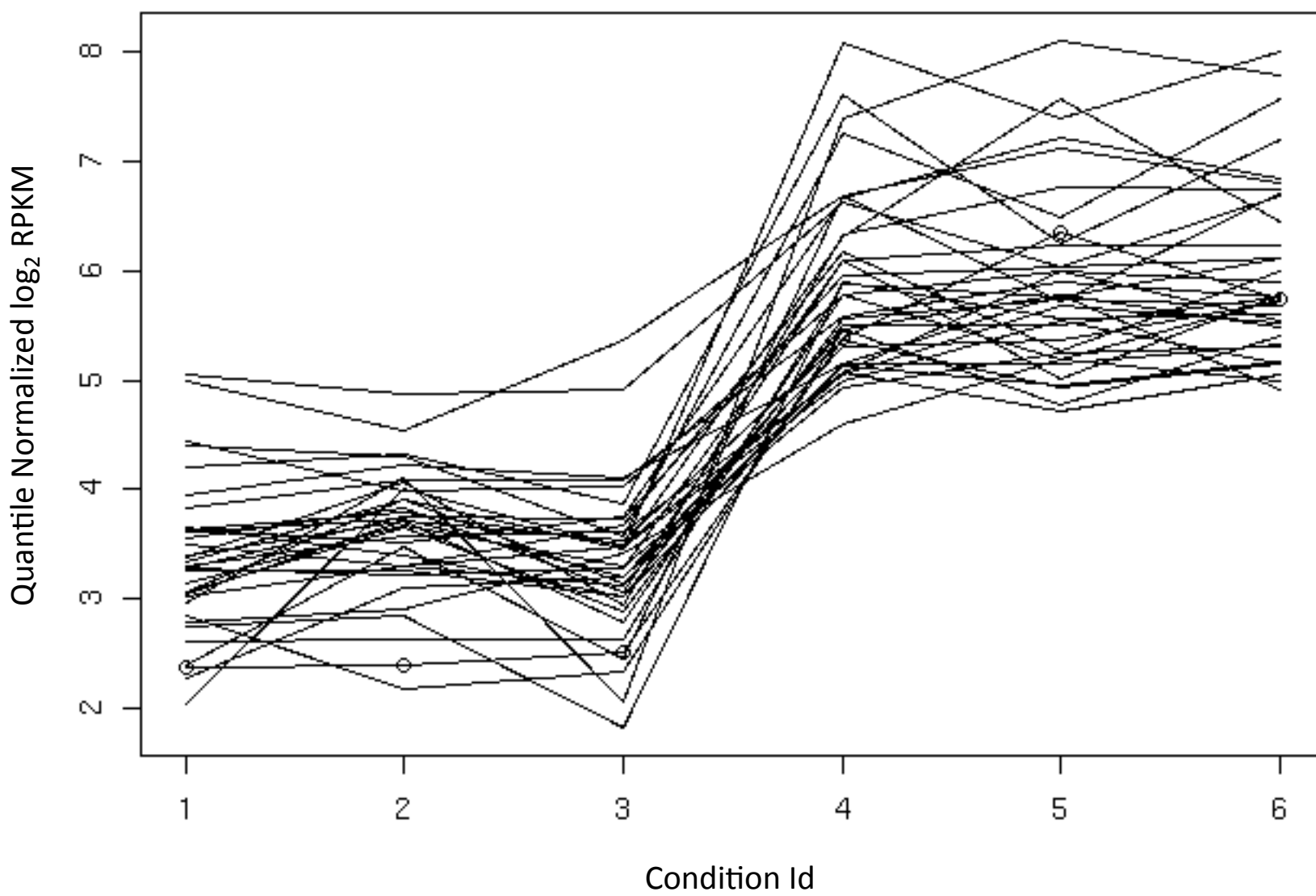

Cluster 2

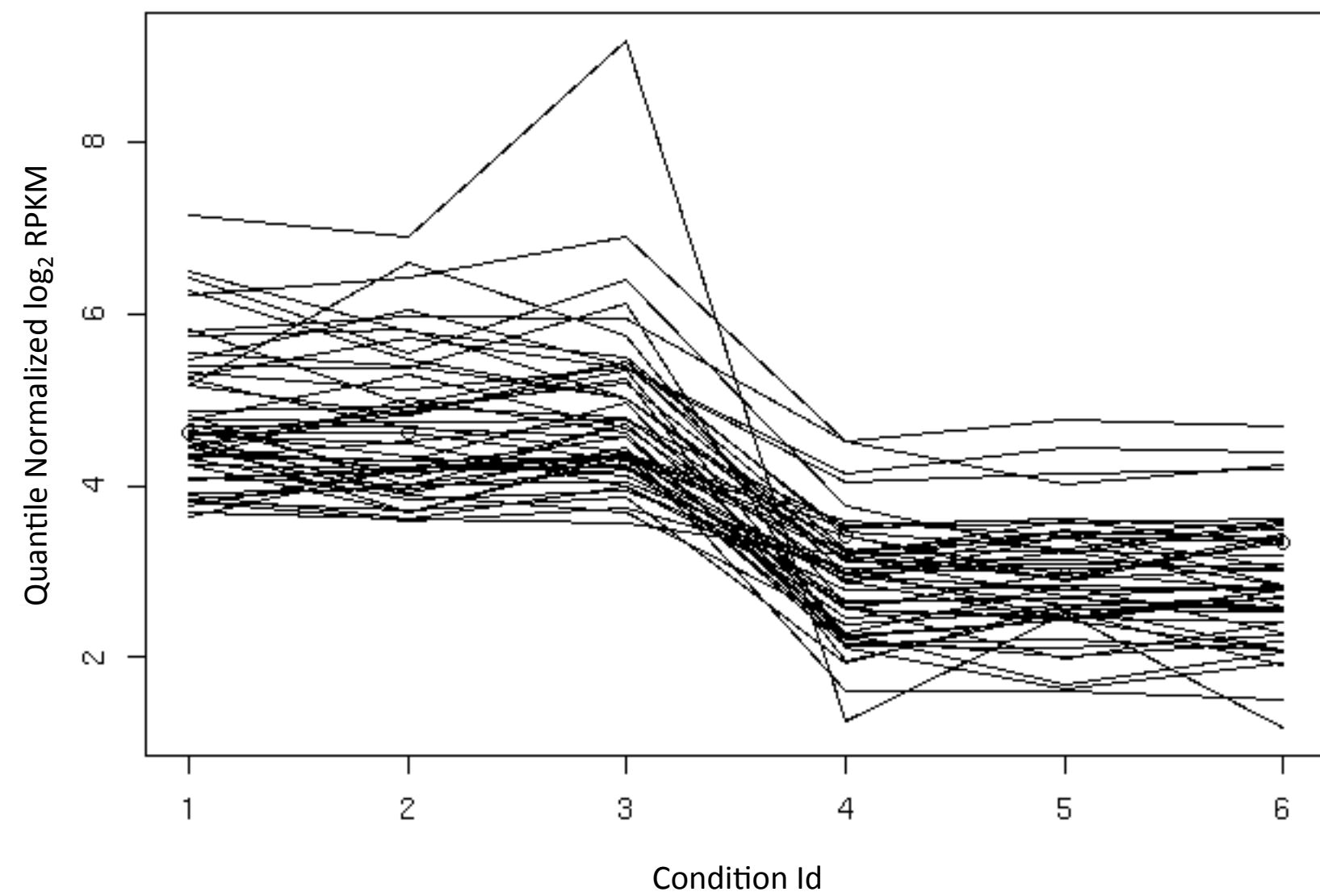

Cluster 3

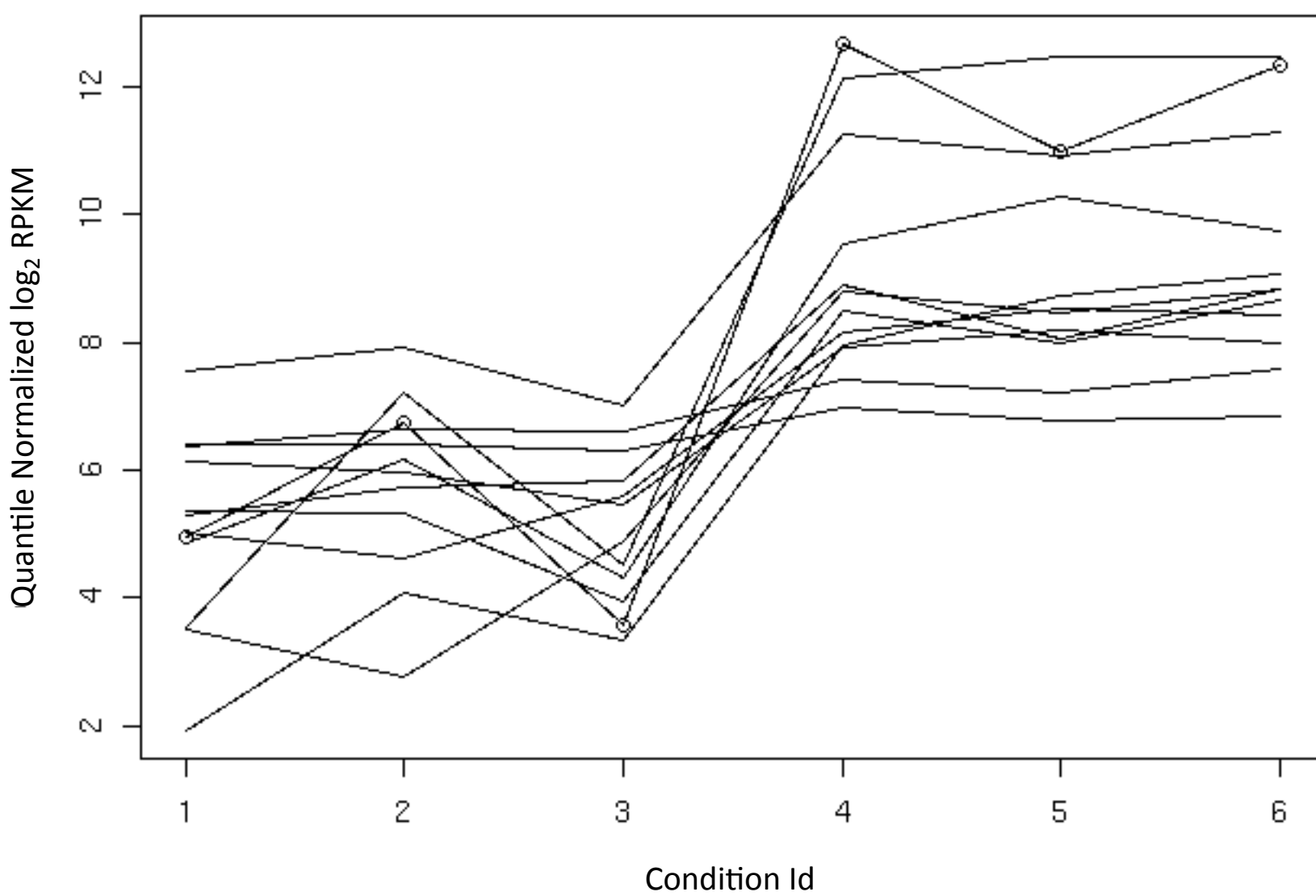

Cluster 4

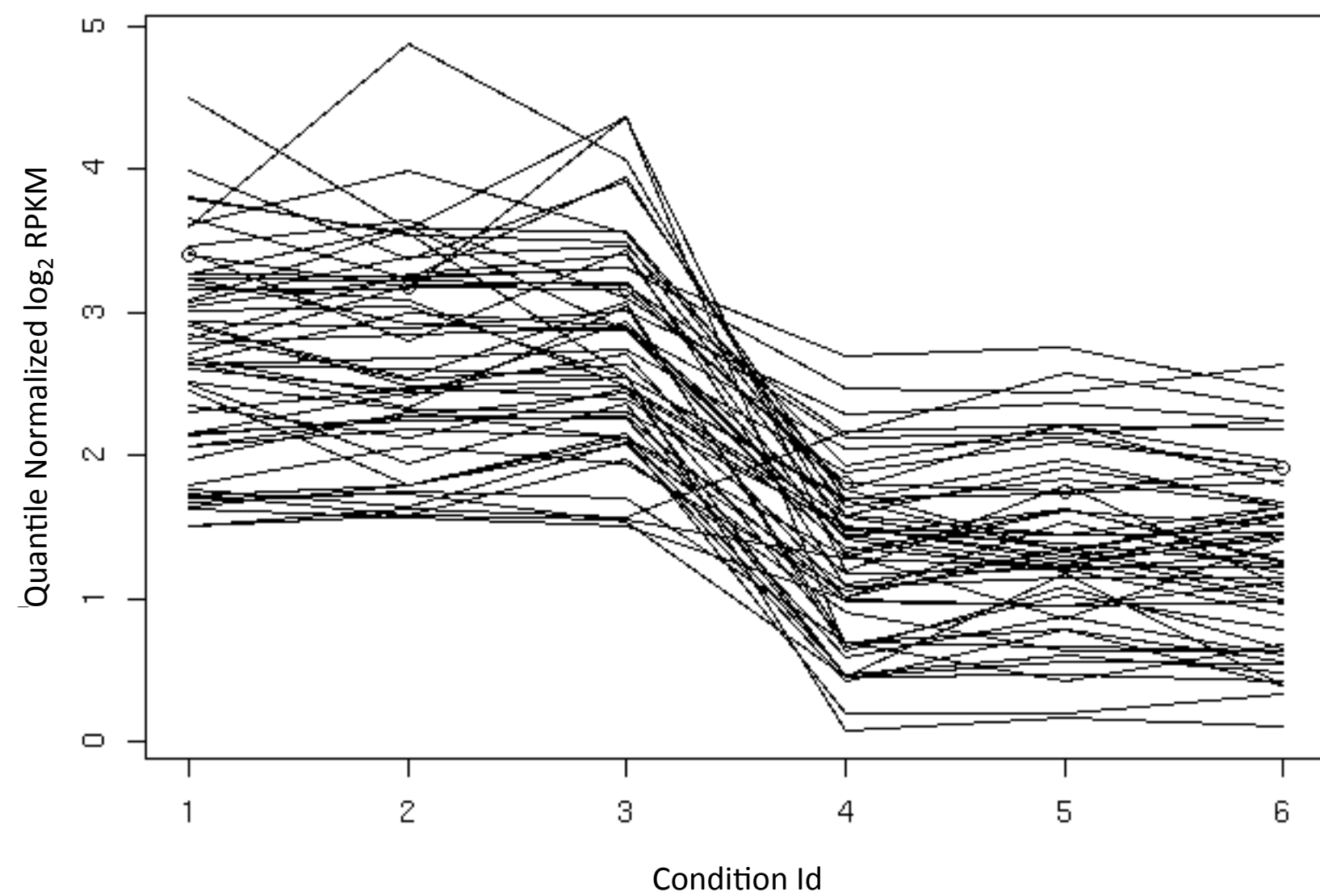

Supplement: Figure S11 — Clustered Gene Co-expression 111. As described for Figure S9, this illustrates the gene expression patterns for all genes correlated with the 111 compound profile, where each plot represents a single cluster of genes. (PDF) [file pgen.1002558.s011.pdf]

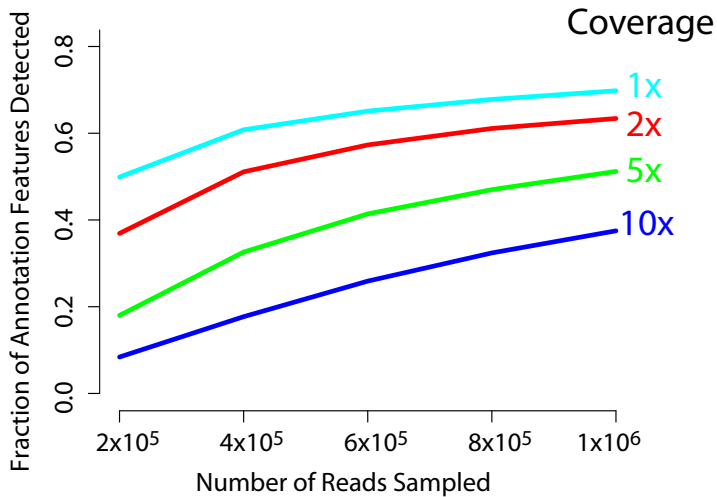

Supplement: Figure S12 — The fraction of genes detected with 1, 2, 5, and 10× read coverage, respectively, at different sub-samplings of the 454 long reads. (PDF) [file pgen.1002558.s012.pdf]

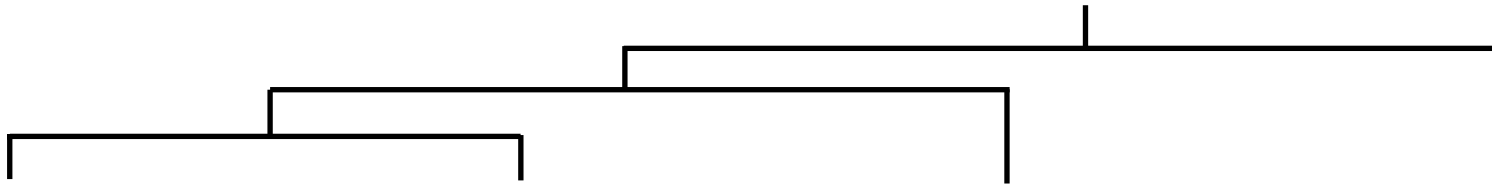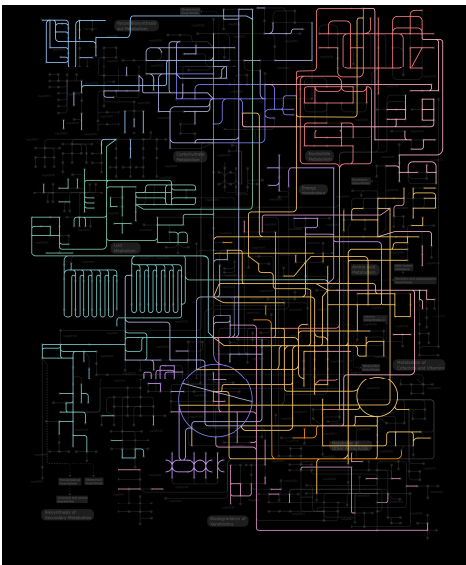

*A. sarcoides*

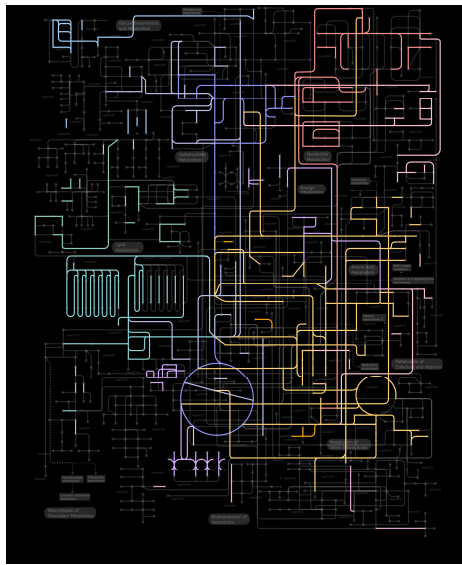

*S. sclerotiorum*

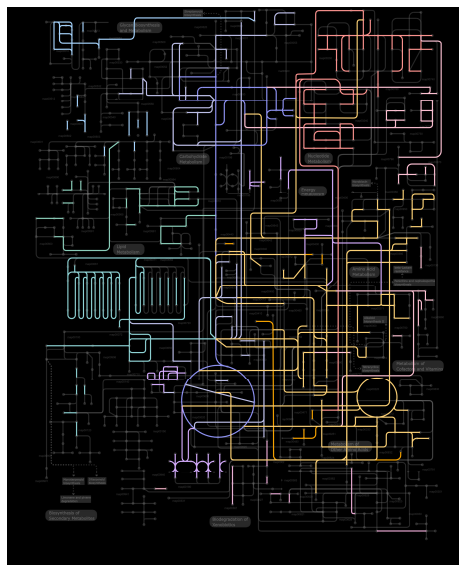

*G. zeae*

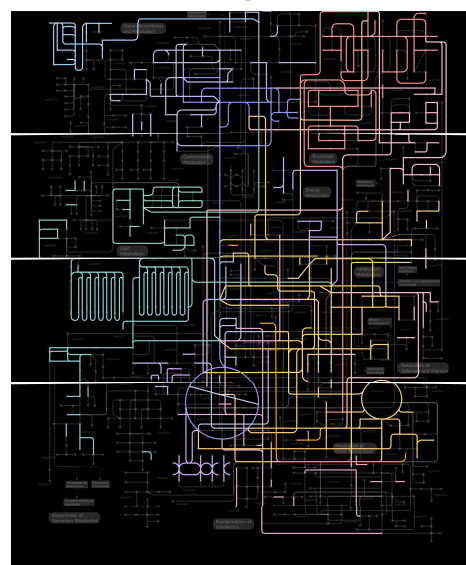

*S. cerevisiae*

Supplement: Figure S13 — Metabolic mapping of KEGG orthologs for A. sarcoides, S.sclerotiorum, G. zeae, S. cerevisiae. Nodes are compounds and connecting lines are enzymes. Color codes are based on functional category. A node can appear in multiple places. The tree is just for illustrative purposes; the branch lengths are not drawn to scale. Generated via iPath. (PDF) [file pgen.1002558.s013.pdf]
