# Supplementary material for: Genomic Analysis of the Hydrocarbon-Producing, Cellulolytic, Endophytic Fungus Ascocoryne sarcoides
Source: PLoS Genet. 2012 Mar 1;8(3):e1002558. doi: 10.1371/journal.pgen.1002558 (PMC3291568; doi:10.1371/journal.pgen.1002558)

Novel TAR Example Read Stacks

Novel TAR as possible missed gene call

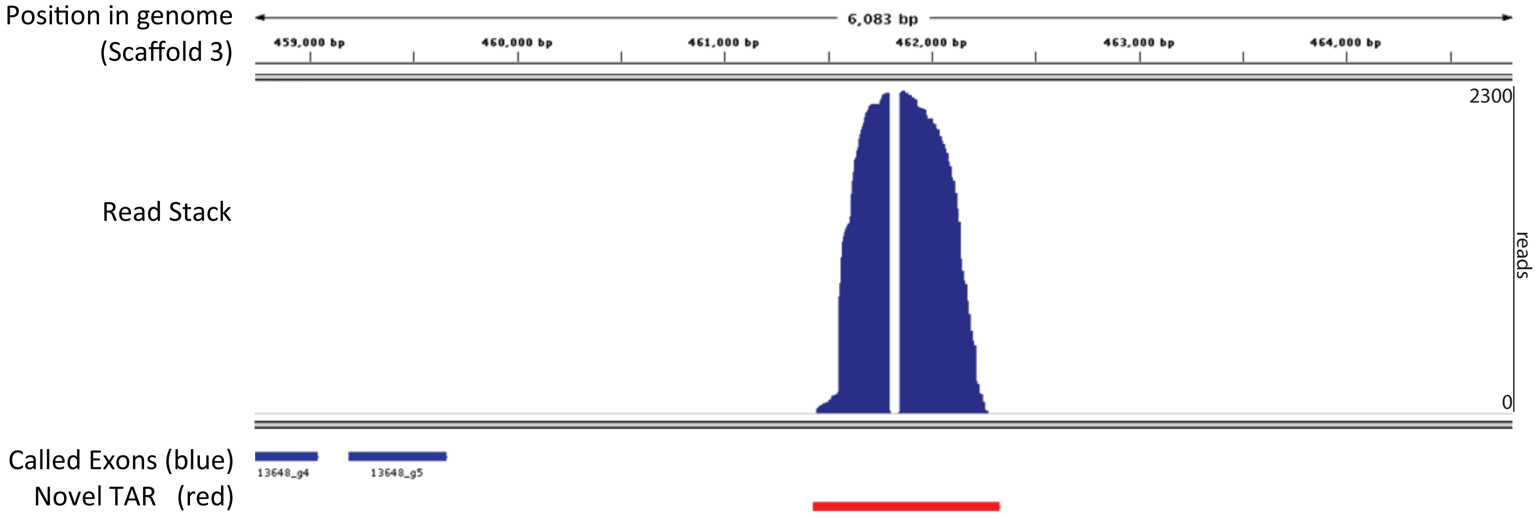

Novel TAR as possible untranslated region

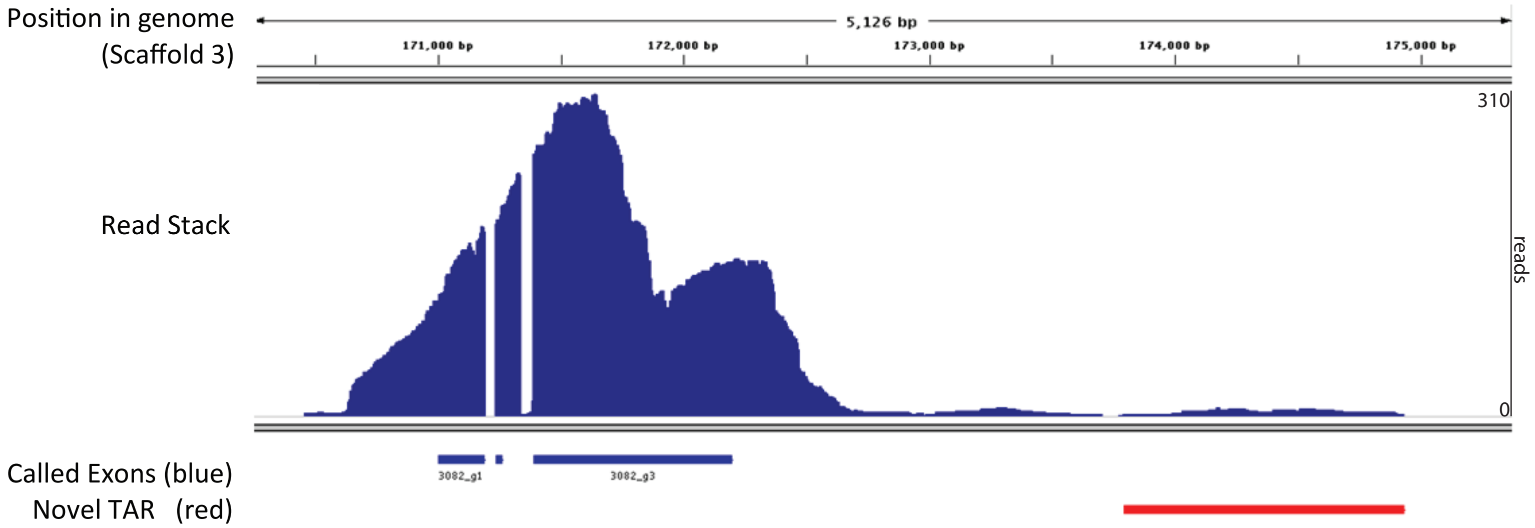

Supplement: Figure S4 — Examples of novel TAR read stacks. The top panel illustrates a well-defined novel TAR that is most likely a missed gene call. The bottom panel demonstrates another novel TAR that is at least 1 kb away from any annotated genes and yet remains part of a long expression tail adjacent to an actively translated exon. (PDF) [file pgen.1002558.s004.pdf]
