# Supplementary material for: Genomic Analysis of the Hydrocarbon-Producing, Cellulolytic, Endophytic Fungus Ascocoryne sarcoides
Source: PLoS Genet. 2012 Mar 1;8(3):e1002558. doi: 10.1371/journal.pgen.1002558 (PMC3291568; doi:10.1371/journal.pgen.1002558)
Supplement: Figure S14 — (A) A representative image of the synteny between S. sclerotiorum and A. sarcoides. The A. sarcoides scaffolds are stacked on the right-hand side and S. sclerotiorum scaffolds are shown in the colored inset. Like-colored regions of A. sarcoides scaffolds and those of S. sclerotiorum represent syntenic blocks. (B) Table reports the total number of orthologs and the levels of synteny between A. sarcoides and each of the four fungi analyzed. (PDF) [file pgen.1002558.s014.pdf]

A.

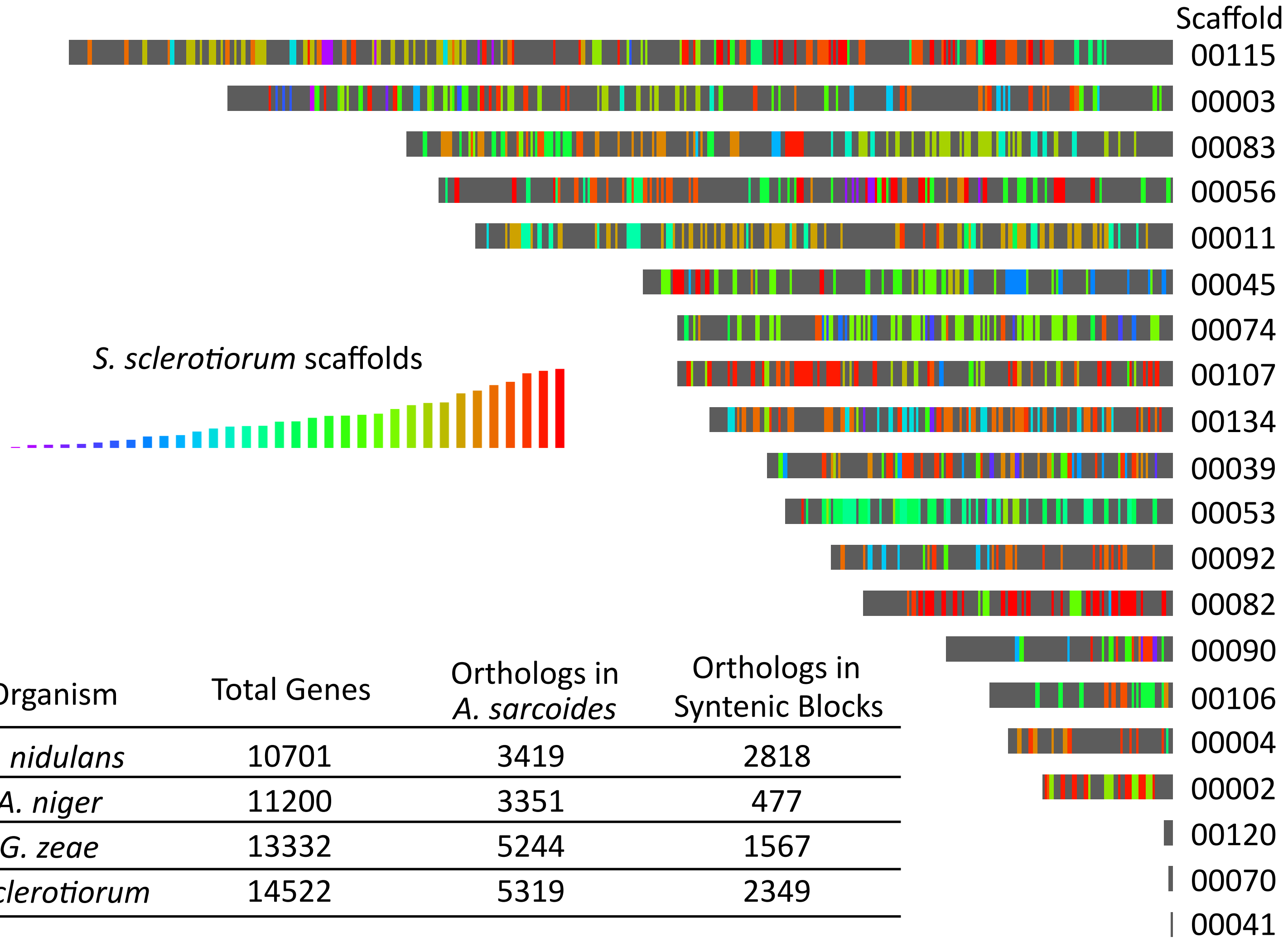

B.

Organism	Total Genes	Orthologs in *A. sarcoides*	Orthologs in Syntenic Blocks
A. nidulans	10701	3419	2818
A. niger	11200	3351	477
G. zeae	13332	5244	1567
S. sclerotiorum	14522	5319	2349
