# Supplementary material for: Genomic Analysis of the Hydrocarbon-Producing, Cellulolytic, Endophytic Fungus Ascocoryne sarcoides
Source: PLoS Genet. 2012 Mar 1;8(3):e1002558. doi: 10.1371/journal.pgen.1002558 (PMC3291568; doi:10.1371/journal.pgen.1002558)
Supplement: Table S1 — Volatile compounds detected and identified via SPME-GC/MS from the headspace of NRRL 50072 samples. NRRL 50072 was cultured as described in the Materials & Methods and in Table S2 with the following conditions: Acetate (OAC), cellulose (CELL), cellobiose (CB), ammonium starvation (AMM), potato dextrose broth at 4 days (PD4), and 14 days (PD14). For each compound, 1 designates production/detection and 0 designates no detection. RT = retention time in minutes. Asterisk (*) designates compound retention time and EI spectra matched that of a pure standard. (PDF) [file pgen.1002558.s015.pdf]

	RT	CB	PD4	PD14	CELL	OAC	AMM
Alkanes / Alkenes							
2-pentene *	1.70	1	0	1	0	1	1
heptane *	2.10	1	1	0	0	0	0
octane *	2.90	1	1	0	0	0	0
1-octene *	3.42	0	0	1	0	0	0
1,3-octadiene	5.46	0	0	1	0	0	0
1,3-trans-5-*cis*-octatriene	8.54	0	0	1	0	0	0
nonane *	4.39	1	0	0	0	0	0
Alcohols							
1-propanol *	7.53	1	1	0	0	0	0
2-methyl-1-propanol *	8.82	1	1	1	1	0	1
1-butanol	9.89	1	1	0	0	0	0
3-methyl-1-butanol *	11.23	1	1	1	1	1	1
3-methyl-3-buten-1-ol	12.18	1	1	1	0	0	0
3-methyl-2-buten-1-ol	13.64	1	1	0	0	0	0
2-methyl-1-pentanol	13.16	1	1	0	0	0	0
3-methyl-1-pentanol	13.70	0	1	0	0	0	0
4-methyl-1-pentanol	13.44	1	1	0	0	0	0
1-hexanol *	14.23	1	1	0	0	1	1
5-methyl-1-hexanol	15.36	0	1	0	0	0	0
1-heptanol *	16.19	1	1	0	0	0	0
3-octanol *	14.98	0	0	1	0	0	0
1-octen-3-ol *	15.83	0	0	1	0	0	0
2-octen-1-ol *	16.09	1	1	1	0	0	0
1,5-octadien-3-ol	16.53	0	0	1	0	0	0
phenyl methanol	23.59	1	1	0	0	0	0
2-phenyl ethanol *	24.14	1	1	0	0	0	0
Ketones / Aldehdyes							
hexanal	8.35	0	0	0	0	1	1
5-methylene-3-heptanone	13.46	0	0	1	0	0	0
3-octanone *	12.24	1	1	1	0	0	0
1-octene-3-one *	12.94	0	0	1	0	0	0
2-nonanone *	14.99	1	0	0	0	0	0
Esters							
ethyl acetate *	4.24	0	1	0	0	0	0
propyl acetate	6.15	0	1	0	0	0	0
2-methyl-1-propyl acetate *	6.98	1	1	0	0	0	0
2-methyl-1-butyl acetate	8.24	0	1	0	0	0	0
3-methyl-1-butyl acetate *	9.33	1	1	0	0	0	0
heptyl acetate *	14.65	1	1	0	0	0	0
octyl acetate *	16.59	0	1	0	0	0	0
2-phenylethyl acetate *	22.62	0	1	0	0	0	0
ethyl propionate	5.80	0	1	0	0	0	0
3-methyl-1-butyl propionate	8.40	0	1	0	0	0	0
pentyl propionate	11.90	1	1	0	0	0	0
methyl 3-methyl-1-butanoate	7.08	1	1	1	0	0	0
ethyl 3-methyl-1-butanoate	8.13	0	1	0	0	0	0
methyl 2-ethyl hexanoate	12.31	1	0	0	0	0	0
methyl ester leucine	13.11	1	1	1	0	0	0
Acids							
acetic acid *	16.65	0	1	0	0	0	1
propanoic acid	18.41	0	1	0	0	0	0
2-methyl-1-propanoic acid	18.85	0	1	0	0	0	0
