# Supplementary material for: Genomic Analysis of the Hydrocarbon-Producing, Cellulolytic, Endophytic Fungus Ascocoryne sarcoides
Source: PLoS Genet. 2012 Mar 1;8(3):e1002558. doi: 10.1371/journal.pgen.1002558 (PMC3291568; doi:10.1371/journal.pgen.1002558)
Supplement: Table S2 — Culture growth conditions for GC/MS profiling and RNA preparation. NRRL50072 was cultured using the stated media, volumes, inoculation/growth conditions, and the RNA preparation and GC/MS analysis were performed on the days listed. (PDF) [file pgen.1002558.s016.pdf]

	CB	PD4	PD14	AMM	CELL	OAC	PD9
**Inoculation Source**	200 mg from filtered liquid culture	200 mg from filtered liquid culture	5 mm culture plug	50 mg from filtered liquid culture	50 mg from filtered liquid culture	50 mg from filtered liquid culture	200 mg from filtered liquid culture
**Media Description**	Minimal media + cellobiose (20 g/L), pH 6.0	Potato Dextrose Broth (24 g/L, EMD Chemicals), dH2O	Potato Dextrose Broth (24 g/L, EMD Chemicals), well water	Minimal Media lacking ammonium chloride + glucose (83.3 mM), pH 6.0	Minimal media + cellulose (15 g/L), pH 6.0	Minimal media + sodium acetate (50 mM), pH 6.0	Potato Dextrose Broth (24 g/L, BD Difco), dH_2O
**Media Volume**	100 mL	100 mL	100 mL	5 mL	5 mL	5 mL	1 L
**Growth conditions**	23°C, 150 rpm	23°C, 150 rpm	23°C, 150 rpm	23°C, 0 rpm	23°C, 0 rpm	23°C, 0 rpm	23°C, 150 rpm
**Culture Age for GC/MS**	4 days	4 days	14 days	2 days	2 days	2 days	N/A
**Culture Age for RNA Harvest**	4 days	4 days	14 days	2 days	2 days	2 days	9 days
