# Supplementary material for: Genomic Analysis of the Hydrocarbon-Producing, Cellulolytic, Endophytic Fungus Ascocoryne sarcoides
Source: PLoS Genet. 2012 Mar 1;8(3):e1002558. doi: 10.1371/journal.pgen.1002558 (PMC3291568; doi:10.1371/journal.pgen.1002558)
Supplement: Table S3 — Transcriptome Mapping Statistics. Report of Illumina and 454 reads mapped to gene models and to genome for each of the conditions and time points. (PDF) [file pgen.1002558.s017.pdf]

Condition Id	RNAseq Run	Sample Condition	Replicate	Total Reads	Reads Mapped to Genes	%	Reads Mapped to Scaffold	%
1	Illumina run 3	CB	A	28,750,774	15,307,498	53	21,406,859	74
2	Illumina run 3	PD4	A	26,968,887	13,575,252	50	18,711,554	69
3	Illumina run 3	PD14	A	25,606,597	14,516,507	57	20,068,118	78
4	Illumina run 1	AMM	A	16,317,806	2,803,234	17	3,855,960	24
	Illumina run 1	AMM	B	8,425,916	2,440,099	29	3,510,518	42
5	Illumina run 1	CELL	A	15,955,509	8,338,030	52	10,846,810	68
	Illumina run 1	CELL	B	15,670,689	4,088,942	26	11,367,443	73
6	Illumina run 1	OAC	A	15,963,762	9,021,384	57	12,216,397	77
	Illumina run 1	OAC	B	15,892,197	8,643,532	54	12,285,295	77
NA	Illumina run 1	PD9	A	16,115,346	8,447,771	52	11,059,134	69
	Illumina run 2	PD9	B	14,731,788	5,348,241	36	7,492,491	51
NA	454 run 1	PD9	A	1,200,000	1,044,000	87		
