# Supplementary material for: Genomic Analysis of the Hydrocarbon-Producing, Cellulolytic, Endophytic Fungus Ascocoryne sarcoides
Source: PLoS Genet. 2012 Mar 1;8(3):e1002558. doi: 10.1371/journal.pgen.1002558 (PMC3291568; doi:10.1371/journal.pgen.1002558)
Supplement: Table S4 — The number of genes from each sequenced organism with homologs in the CAZY database per CAZY class. Glycosyl hydrolases (GH), glycosyl transferases (GT), Carbohydrate-binding module (CBM), carbohydrate esterase (CE), and polylyase (PL). Table structure adapted from Martinez et al [7]. (PDF) [file pgen.1002558.s018.pdf]

Organism	GH	GT	CBM	CE	PL
A. sarcoides	169	56	52	22	1
T. reesei	200	103	36	16	3
A. nidulans	247	91	36	29	19
G. zeae	243	110	61	42	20
Avg Eurotiomycetes	265	103	40	28	18
Avg Sordariomycetes	211	96	49	32	8
Avg Saccharomyces	47	70	9	3	0
Avg Archiascomycetes	46	61	5	5	0
Avg Basidiomycetes	75	68	10	9	3
