# Supplementary material for: Genomic Analysis of the Hydrocarbon-Producing, Cellulolytic, Endophytic Fungus Ascocoryne sarcoides
Source: PLoS Genet. 2012 Mar 1;8(3):e1002558. doi: 10.1371/journal.pgen.1002558 (PMC3291568; doi:10.1371/journal.pgen.1002558)
Supplement: Table S5 — Genes identified in the A. sarcoides genome with homologs in plants. Genes are subdivided into three classes: P, genes with exclusively plant orthologs; M, genes with mostly plant orthologs; and N, genes that did not have a plant ortholog, but bordered a set of plant orthologs. (PDF) [file pgen.1002558.s019.pdf]

Gene ID	Class	Description
AS3618	P	thaumatin pathogenicity
AS3619	P	thaumatin
AS4407	P	plant protein of unknown function, DUF946
AS4441	P	Magnesium and cobalt transporter
AS5280	P	predicted protein
AS6904	P	Short chain alpha hydroxy acid oxidase
AS6914	P	putative polyvinyl alcohol dehydrogenase; quinonprotein alcohol dehydrogenase
AS9718	P	beta-glucan elicitor
AS3617	M	2-oxoglutarate-dependent dioxygenase
AS4457	M	Mannan endo-1,4-beta-mannosidase (plant + Paenibacillus, bacteria)
AS21522	M	Lysine methyltransferase enzymes (plants + B. fuckeliana)
AS6781	N	Flavin-binding
AS6783	N	Lysine methyltransferase enzyme
