# Supplementary material for: Genomic Analysis of the Hydrocarbon-Producing, Cellulolytic, Endophytic Fungus Ascocoryne sarcoides
Source: PLoS Genet. 2012 Mar 1;8(3):e1002558. doi: 10.1371/journal.pgen.1002558 (PMC3291568; doi:10.1371/journal.pgen.1002558)
Supplement: Table S6 — Targeted search for β-ketosynthase (KS) and acyltransferase (AT) domains. Genes identified in the targeted search are listed with their domain annotations. For genes that were part of clusters, the additional genes found within the cluster are also included. (PDF) [file pgen.1002558.s020.pdf]

GeneID	Domain
**Unclustered**	
AS1954	malonyl CoA acyl carrier protein transacylase (AT)
AS4844	Acetyl CoA Acyltransferase (KS)
AS4891	NRPS: Adenylation, PCP, Acyl CoA reductase
AS5198	isopenicillin-N-acyltransferase (AT)
AS7847	chitin synthase (AT)
AS8458	KS, AT, MT, alcohol dehydrogenase, KR
AS8701	Hybrid NRPS: KS, AT, MT, KR, Condensation, AMP binding, ACP, Acyl CoA reductase
AS9587	match to PKS no other annotation
AS10151	match to PKS no other annotation
AS10597	KS, AT, MT, alcohol dehydrogenase, KR, ACP
**Cluster 1**	
AS8052	amino acid permease
AS8053	Major facilitator superfamily
AS8054	AT, alcohol dehydrogenase, KR
AS8055	confers resistance to aminoglycosides
**Cluster 2**	
AS1080	Multidrug resistance family
AS1081	NO homolog
AS1082	beta-ketoacyl synthase (KS) (elongation step in fatty acid biosynthesis)
**Cluster 3**	
AS1985	Enoyl CoA hydratase (KS)
AS8313	Enoyl CoA hydratase (KS)
**Cluster 4**	
AS8009	FAS beta subunit dehydrogenase (AT)
AS8010	FAS alpha subunit (AT, KS, PT)
**Cluster 5**	
AS8786	aryl alcohol dehydrogenase
AS8787	Patatin-like phospholipase (plant storage enzyme) (KS)
AS8788	KR-like, DH (epimerase)-like
AS8789	AA permease
AS8790	NONclassical export/Major facilitator superfamily
AS13172	xenobiotic monooxygenase
**Cluster 6**	
AS3003	NO homolog
AS3004	Chalcone synthase
AS3005	Major faciliitator superfamily
**Cluster 7**	
AS2529	Adenylation, PCP, Acyl CoA reductase/KR, DH (epimerase)
AS2530	Mandelate racemase/muconate lactonizing enzymes involved in aromatic amino acid catabolism
