# Supplementary material for: Genomic Analysis of the Hydrocarbon-Producing, Cellulolytic, Endophytic Fungus Ascocoryne sarcoides
Source: PLoS Genet. 2012 Mar 1;8(3):e1002558. doi: 10.1371/journal.pgen.1002558 (PMC3291568; doi:10.1371/journal.pgen.1002558)
Supplement: Table S7 — Targeted search for enoyl reductase (ER), dehydratase (DH), and ketoreductase (KR) domains. Genes identified in the targeted search are listed with their domain annotations. For genes that were part of clusters, the additional genes found within the cluster are also included. (PDF) [file pgen.1002558.s021.pdf]

GeneID	PFAM ID	Short Description
**Unclustered**		
AS9422	106/1370	3-oxoacyl carrier protein reductase
AS10819	106/3435/13 70/8659	short chain dehydrogenae, sacchoripine dehydrogenase, epimerase, KR
**Cluster 1**		
AS5894	7690	MFS efflux pump
AS5895	106/1370	short chain dehydrogenase reductase
AS5896	107	2-alkenol reductase
AS5898	83/7690	MFS maltose permease
AS5899	-	NO Homology
**Cluster 2**		
AS8804	1370	Cinnamoyl-CoA reductase
AS8805	1738	Dienolcatone hydrolase
**Cluster 3**		
AS7856	8240/107	alcohol dehydrogenase, KR, sorbitol dehydrogenase
AS7857	106/8659/1370	Short chain dehydrogenase, epimerase
AS7858	5153	inositol oxygenase
**Cluster 4**		
AS47	1370	epimerase
AS48	1370/7993	epimerase/KR
AS49	4909	amidohydrolase
**Cluster 5**		
AS2093	1522	polysaccharide deacetylase
AS2094	107/106	zinc binding dehydrogenase/short chain
AS2095	1370/5368	epimerase/NmrA/hscarg dehydrogenase
**Cluster 6**		
AS7950	171	succinate-semialdehyde dehydrogenase
AS7951	106/1370	short chain dehydrogenase/epimerase
**Cluster 7**		
AS2076	-	zinc finger
AS2077	-	ketoreductase
AS2078	1370/7993/4082	fungal TF
**Cluster 8**		
AS10496	8659/106/3435	carbonyl reductase
AS10497	-	KR/saccharopine dehydrogenase
AS10498	1370/7993	hypothetical
