# Supplementary material for: Genomic Analysis of the Hydrocarbon-Producing, Cellulolytic, Endophytic Fungus Ascocoryne sarcoides
Source: PLoS Genet. 2012 Mar 1;8(3):e1002558. doi: 10.1371/journal.pgen.1002558 (PMC3291568; doi:10.1371/journal.pgen.1002558)
Supplement: Table S8 — Gene Subset Co-expressed with the 001 Compound Profile. For each of the following 6 tables (Tables S8, S9, 10, S11, S12, S13): Gene ID, gene ID within A. sarcoides; Status, reports if the gene is active (A) or repressed (R) in the production conditions; KO, KEGG ortholog ID; Description, description of the KEGG ortholog; EC, lists the Enzyme Commission number that corresponds to the KEGG ortholog, where relevant. (PDF) [file pgen.1002558.s022.pdf]

Gene ID	Type	KO	Description	EC
AS10218	A	K07188	hormone-sensitive lipase	3.1.1.79
AS3724	R	K13993	HSP20 family protein	NONE
AS8173	R	K13535	cardiolipin-specific phospholipase	3.1.1.-
AS6923	R	K04716	sphingosine-1-phosphate phosphatase 1	3.1.3.-
AS2883	A	K11816	YUCCA family monooxygenase	1.14.13.-
AS498	A	K12412	minichromosome maintenance protein 1	NONE
AS2143	R	K00999	CDP-diacylglycerol--inositol 3-phosphatidyltransferase	2.7.8.11
AS919	R	K01874	methionyl-tRNA synthetase	6.1.1.10
AS357	A	K08139	MFS transporter, SP family, sugar:H+ symporter	NONE
AS4820	A	K00059	3-oxoacyl-[acyl-carrier protein] reductase	1.1.1.100
AS7095	A	K00058	D-3-phosphoglycerate dehydrogenase	1.1.1.95
AS6242	A	K01897	long-chain acyl-CoA synthetase	6.2.1.3
AS1078	R	K00326	cytochrome-b5 reductase	1.6.2.2
AS6344	R	K01809	mannose-6-phosphate isomerase	5.3.1.8
AS2190	R	K10027	phytoene dehydrogenase	1.14.99.-
AS4269	R	K12462	Rho GDP-dissociation inhibitor	NONE
AS6828	A	K10413	dynein heavy chain 1, cytosolic	NONE
AS5823	A	K01452	chitin deacetylase	3.5.1.41
AS2462	A	K03333	cholesterol oxidase	1.1.3.6
AS4142	A	K01836	phosphoacetylglucosamine mutase	5.4.2.3
AS5126	R	K00390	phosphoadenosine phosphosulfate reductase	1.8.4.8
AS2804	A	K11987	prostaglandin-endoperoxide synthase 2	1.14.99.1
AS137	R	K07127	5-hydroxyisourate hydrolase	3.5.2.17
AS5990	R	K00134	glyceraldehyde 3-phosphate dehydrogenase	1.2.1.12
AS9097	R	K01755	argininosuccinate lyase	4.3.2.1
AS3405	A	K11987	prostaglandin-endoperoxide synthase 2	1.14.99.1
AS9154	R	K10245	fatty acid elongase 2	2.3.1.-
AS7429	R	K01490	AMP deaminase	3.5.4.6
AS5565	A	K00059	3-oxoacyl-[acyl-carrier protein] reductase	1.1.1.100
AS1593	A	K00059	3-oxoacyl-[acyl-carrier protein] reductase	1.1.1.100
AS3963	R	K01443	N-acetylglucosamine-6-phosphate deacetylase	3.5.1.25
