# Supplementary material for: Genomic Analysis of the Hydrocarbon-Producing, Cellulolytic, Endophytic Fungus Ascocoryne sarcoides
Source: PLoS Genet. 2012 Mar 1;8(3):e1002558. doi: 10.1371/journal.pgen.1002558 (PMC3291568; doi:10.1371/journal.pgen.1002558)
Supplement: Table S9 — Gene Subset Co-expressed with the 010 Compound Profile. Gene ID, gene ID within A. sarcoides; Status, reports if the gene is active (A) or repressed (R) in the production conditions; KO, KEGG ortholog ID; Description, description of the KEGG ortholog; EC, lists the Enzyme Commission number that corresponds to the KEGG ortholog, where relevant. (PDF) [file pgen.1002558.s023.pdf]

Gene ID	Type	KO	Description	EC
AS6394	A	K00002	alcohol dehydrogenase (NADP+)	1.1.1.2
AS3884	A	K01904	4-coumarate--CoA ligase	6.2.1.12
AS8113	A	K00463	indoleamine 2,3-dioxygenase	1.13.11.52
AS4736	A	K11992	acyl-CoA thioesterase 8	3.1.2.27
AS7059	A	K03787	5'-nucleotidase	3.1.3.5
AS2279	A	K01720	2-methylcitrate dehydratase	4.2.1.79
AS6359	A	K03004	DNA-directed RNA polymerase I subunit A43	2.7.7.6
AS9509	A	K03016	DNA-directed RNA polymerase II subunit H	NONE
AS6643	A	K02896	large subunit ribosomal protein L24e	NONE
AS3173	A	K00762	orotate phosphoribosyltransferase	2.4.2.10
AS6530	A	K14002	DnaJ-related protein SCJ1	NONE
AS14524	A	K09838	zeaxanthin epoxidase	1.14.13.90
AS15130	A	K00599	NA	2.1.1.-
AS8909	A	K00294	1-pyrroline-5-carboxylate dehydrogenase	1.5.1.12
AS1476	R	K00900	6-phosphofructo-2-kinase	2.7.1.105
