# Supplementary material for: Genomic Analysis of the Hydrocarbon-Producing, Cellulolytic, Endophytic Fungus Ascocoryne sarcoides
Source: PLoS Genet. 2012 Mar 1;8(3):e1002558. doi: 10.1371/journal.pgen.1002558 (PMC3291568; doi:10.1371/journal.pgen.1002558)
Supplement: Table S10 — Gene Subset Co-expressed with the 100 Compound Profile. Gene ID, gene ID within A. sarcoides; Status, reports if the gene is active (A) or repressed (R) in the production conditions; KO, KEGG ortholog ID; Description, description of the KEGG ortholog; EC, lists the Enzyme Commission number that corresponds to the KEGG ortholog, where relevant. (PDF) [file pgen.1002558.s024.pdf]

Gene ID	Type	KO	Description	EC
AS1423	A	K03064	26S proteasome regulatory subunit T4	NONE
AS9197	A	K05658	ATP-binding cassette, subfamily B (MDR/TAP), member 1	NONE
AS9946	A	K03781	c2a0tSalparsoeteasome subunit	1.11.1.6
AS8510	A	K02725	alpha 6	3.4.25.1
AS5758	A	K01184	polygalacturonase	3.2.1.15
AS3974	A	K01279	tripeptidyl-peptidase I	3.4.14.9
AS7547	A	K00801	farnesyl-diphosphate farnesyltransferase	2.5.1.21
AS2790	A	K03062	26S proteasome regulatory subunit T2	NONE
AS7040	A	K05279	flavonol 3-O- methyltransferase	2.1.1.76
AS10319	A	K08739	DNA mismatch repair protein MLH3	NONE
AS5376	A	K00599	-	2.1.1.-
AS5045	A	K13289	cathepsin A	3.4.16.5
AS3892	A	K03065	26S proteasome regulatory subunit T5	NONE
AS17056	A	K01580	glutamate decarboxylase	4.1.1.15
