# Supplementary material for: Genomic Analysis of the Hydrocarbon-Producing, Cellulolytic, Endophytic Fungus Ascocoryne sarcoides
Source: PLoS Genet. 2012 Mar 1;8(3):e1002558. doi: 10.1371/journal.pgen.1002558 (PMC3291568; doi:10.1371/journal.pgen.1002558)
Supplement: Table S11 — Gene Subset Co-expressed with the 101 Compound Profile. Gene ID, gene ID within A. sarcoides; Status, reports if the gene is active (A) or repressed (R) in the production conditions; KO, KEGG ortholog ID; Desc, description of the KEGG ortholog; EC, lists the Enzyme Commission number that corresponds to the KEGG ortholog, where relevant. (PDF) [file pgen.1002558.s025.pdf]

Gene ID	Type	KO	Description	EC
AS6211	R	K00826	branched-chain amino acid aminotransferase	2.6.1.42
AS10184	R	K01497	GTP cyclohydrolase II	3.5.4.25
AS20587	R	K00135	succinate-semialdehyde dehydrogenase (NADP+)	1.2.1.16
AS5446	R	K00826	branched-chain amino acid aminotransferase	2.6.1.42
AS5803	R	K01626	3-deoxy-7-phosphoheptulonate synthase	2.5.1.54
AS8246	R	K09485	heat shock protein 110kDa	NONE
AS8244	R	K12275	translocation protein SEC62	NONE
AS8240	R	K04043	molecular chaperone DnaK	NONE
AS2688	R	K04077	chaperonin GroEL	NONE
AS3224	A	K00009	mannitol-1-phosphate 5-dehydrogenase	1.1.1.17
AS2656	A	K08513	vesicle-associated membrane protein 4	NONE
AS2133	R	K00948	ribose-phosphate pyrophosphokinase	2.7.6.1
AS6254	R	K00290	saccharopine dehydrogenase (NAD+, L-lysine forming)	1.5.1.7
AS6251	R	K01872	alanyl-tRNA synthetase	6.1.1.7
AS9842	R	K00088	IMP dehydrogenase	1.1.1.205
AS4818	R	K01738	cysteine synthase A	2.5.1.47
AS2320	R	K12486	stromal membrane-associated protein	NONE
AS9364	A	K01580	glutamate decarboxylase	4.1.1.15
AS9373	R	K10643	CCR4-NOT transcription complex subunit 4	6.3.2.19
AS3046	A	K11253	histone H3	NONE
AS8251	R	K01259	proline iminopeptidase	3.4.11.5
AS8254	A	K04393	cell division control protein 42	NONE
AS421	A	K07408	cytochrome P450, family 1, subfamily A, polypeptide 1	1.14.14.1
AS12820	A	K00323	NAD(P) transhydrogenase	1.6.1.2
AS10407	A	K00679	phospholipid:diacylglycerol acyltransferase	2.3.1.158
AS7252	R	K08738	cytochrome c	NONE
AS3145	R	K00813	aspartate aminotransferase	2.6.1.1
AS2191	R	K02291	phytoene synthase	2.5.1.32
AS10086	A	K02540	minichromosome maintenance protein 2	NONE
AS3335	R	K00162	pyruvate dehydrogenase E1 component subunit beta	1.2.4.1
AS7560	R	K00026	malate dehydrogenase	1.1.1.37
AS6836	R	K02867	large subunit ribosomal protein L11	NONE
AS9749	R	K01951	GMP synthase (glutamine-hydrolysing)	6.3.5.2
AS9749	R	K01951	GMP synthase (glutamine-hydrolysing)	6.3.5.2
AS8328	R	K08139	MFS transporter, SP family, sugar:H+ symporter	NONE
AS8438	R	K01696	tryptophan synthase beta chain	4.2.1.20
AS2937	R	K00995	CDP-diacylglycerol--glycerol-3-phosphate 3-phosphatidyltransfera	2.7.8.5
AS5127	R	K00764	amidophosphoribosyltransferase	2.4.2.14
AS15092	R	K00052	3-isopropylmalate dehydrogenase	1.1.1.85
AS7645	R	K02365	separase	3.4.22.49
AS6619	R	K10601	E3 ubiquitin-protein ligase synoviolin	6.3.2.19
AS2063	A	K00429	catalase	1.11.1.6
AS2420	R	K00033	6-phosphogluconate dehydrogenase	1.1.1.44
AS9650	A	K08486	syntaxin 1B/2/3	NONE
AS9041	A	K04464	mitogen-activated protein kinase 7	2.7.11.24
AS9070	R	K01953	asparagine synthase (glutamine-hydrolysing)	6.3.5.4
AS9102	R	K10597	ubiquitin conjugation factor E4 B	6.3.2.19
AS5875	A	K08266	G protein beta subunit-like	NONE
AS6001	A	K07904	Ras-related protein Rab-11A	NONE
AS7296	A	K10585	ubiquitin-conjugating enzyme E2 Z	6.3.2.19
AS5561	R	K01673	carbonic anhydrase	4.2.1.1
AS4049	R	K08770	ubiquitin C	NONE
AS1533	R	K11294	nucleolin	NONE
AS7896	A	K03512	DNA polymerase lambda subunit	2.7.7.7, 4.2.99.-
AS7630	R	K02886	large subunit ribosomal protein L2	NONE
AS283	A	K00360	nitrate reductase (NADH)	1.7.1.1
