# Supplementary material for: Genomic Analysis of the Hydrocarbon-Producing, Cellulolytic, Endophytic Fungus Ascocoryne sarcoides
Source: PLoS Genet. 2012 Mar 1;8(3):e1002558. doi: 10.1371/journal.pgen.1002558 (PMC3291568; doi:10.1371/journal.pgen.1002558)
Supplement: Table S12 — Gene Subset Co-expressed with 110 Compound Profile. Gene ID, gene ID within A. sarcoides; Status, reports if the gene is active (A) or repressed (R) in the production conditions; KO, KEGG ortholog ID; Desc, description of the KEGG ortholog; EC, lists the Enzyme Commission number that corresponds to the KEGG ortholog, where relevant. (PDF) [file pgen.1002558.s026.pdf]

Gene ID	Type	KO	Description	EC
AS5832	A	K05754	actin related protein 2/3 complex, subunit 5	NONE
AS2485	A	K02875	large subunit ribosomal protein L14e	NONE
AS5220	A	K01183	chitinase	3.2.1.14
AS3288	A	K01433	formyltetrahydrofolate deformylase	3.5.1.10
AS3295	A	K12670	oligosaccharyltransferase complex subunit beta	NONE
AS5830	A	K02865	large subunit ribosomal protein L10Ae	NONE
AS3509	A	K02951	small subunit ribosomal protein S12e	NONE
AS8620	A	K04708	3-dehydrosphinganine reductase	1.1.1.102
AS3900	A	K12666	oligosaccharyltransferase complex subunit alpha	NONE
AS9329	A	K10215	monooxygenase	1.14.13.-
AS4729	A	K00020	3-hydroxyisobutyrate dehydrogenase	1.1.1.31
AS3406	A	K05396	D-cysteine desulfhydrase	4.4.1.15
AS567	A	K05928	tocopherol O-methyltransferase	2.1.1.95
AS7887	A	K14004	protein transport protein SEC13	NONE
