# Supplementary material for: Genomic Analysis of the Hydrocarbon-Producing, Cellulolytic, Endophytic Fungus Ascocoryne sarcoides
Source: PLoS Genet. 2012 Mar 1;8(3):e1002558. doi: 10.1371/journal.pgen.1002558 (PMC3291568; doi:10.1371/journal.pgen.1002558)
Supplement: Table S13 — Gene Subset Co-expressed with the 111 Compound Profile. Gene ID, gene ID within A. sarcoides; Status, reports if the gene is active (A) or repressed (R) in the production conditions; KO, KEGG ortholog ID; Desc, description of the KEGG ortholog; EC, lists the Enzyme Commission number that corresponds to the KEGG ortholog, where relevant. (PDF) [file pgen.1002558.s027.pdf]

Gene Id	Type	KO	Description	EC
AS5457	A	K10258	enoyl reductase	1.3.1.-
AS10380	A	K08288	protein kinase C substrate 80K-H	NONE
AS3744	R	K11087	small nuclear ribonucleoprotein D1	NONE
AS7833	A	K00140	methylmalonate-semialdehyde dehydrogenase	1.2.1.27
AS9213	A	K01726		4.2.1.-
AS3208	A	K10251	beta-keto reductase	1.1.1.-
AS496	A	K02150	V-type H+-transporting ATPase subunit E	3.6.3.14
AS22	R	K06654	phosphate system cyclin PHO80	NONE
AS10421	R	K02219	cyclin-dependent kinase regulatory subunit CKS1	NONE
AS4842	A	K11099	small nuclear ribonucleoprotein G	NONE
AS2482	R	K13431	signal recognition particle receptor subunit alpha	NONE
AS9367	A	K00166	2-oxoisovalerate dehydrogenase E1 component, alpha subunit	1.2.4.4
AS9438	A	K00167	2-oxoisovalerate dehydrogenase E1 component, beta subunit	1.2.4.4
AS7084	A	K02896	large subunit ribosomal protein L24e	NONE
AS8262	R	K13509	lysophosphatidate acyltransferase	2.3.1.51
AS8262	R	K13509	lysophosphatidate acyltransferase	2.3.1.51
AS1146	R	K01173	endonuclease	3.1.30.-
AS5276	R	K01772	ferrochelatase	4.99.1.1
AS1064	A	K00729	dolichyl-phosphate beta-glucosyltransferase	2.4.1.117
AS7995	A	K02889	large subunit ribosomal protein L21e	NONE
AS7993	A	K12825	splicing factor 3A subunit 1	NONE
AS4493	R	K01476	arginase	3.5.3.1
AS5772	A	K12864	beta-catenin-like protein 1	NONE
AS7170	A	K04499	RuvB-like protein 1 (pontin 52)	NONE
AS10110	A	K01640	hydroxymethylglutaryl-CoA lyase	4.1.3.4
AS9457	R	K02434	aspartyl-tRNA(Asn)/glutamyl-tRNA (Gln) amidotransferase subunit B	6.3.5.6, 6.3.5.7
AS3365	A	K13289	cathepsin A	3.4.16.5
AS3365	A	K13289	cathepsin A	3.4.16.5
AS4272	R	K12397	AP-3 complex subunit beta	NONE
AS9805	A	K00649	glyceronephosphate O-acyltransferase	2.3.1.42
AS9493	A	K07827	GTPase KRas	NONE
AS8346	R	K01807	ribose 5-phosphate isomerase A	5.3.1.6
AS8362	A	K03504	DNA polymerase delta subunit 3	NONE
AS3493	A	K03094	S-phase kinase-associated protein 1	NONE
AS9874	A	K03950	NADH dehydrogenase (ubiquinone) 1 alpha subcomplex 6	1.6.5.3, 1.6.99.3
AS10534	A	K10563	formamidopyrimidine-DNA glycosylase	3.2.2.23, 4.2.99.18
AS5307	R	K04072	acetaldehyde dehydrogenase / alcohol dehydrogenase	1.2.1.10, 1.1.1.1
AS6532	R	K01441	alkaline ceramidase	3.5.1.23
AS2025	A	K02537	mitotic spindle assembly checkpoint protein MAD2	NONE
AS75	R	K00637	sterol O-acyltransferase	2.3.1.26
AS8837	A	K12580	CCR4-NOT transcription complex subunit 3	NONE
AS9062	A	K02973	small subunit ribosomal protein S23e	NONE
AS4373	R	K03360	F-box and leucine-rich repeat protein GRR1	NONE
AS4378	R	K06655	negative regulator of the PHO system	2.7.11.22
AS21299	A	K08764	sterol carrier protein 2	2.3.1.176
AS5956	R	K03131	transcription initiation factor TFIID subunit D5	NONE
AS1306	A	K12827	splicing factor 3A subunit 3	NONE
AS3891	R	K03124	transcription initiation factor TFIIB	NONE
AS7449	A	K01897	long-chain acyl-CoA synthetase	6.2.1.3
AS4756	A	K03139	transcription initiation factor TFIIF beta subunit	NONE
AS8936	A	K13341	peroxin-7	NONE
AS5598	A	K03140	transcription initiation factor TFIIF small subunit	NONE
AS1654	R	K04710	Acyl-CoA-dependent ceramide synthase	2.3.1.24
AS8692	R	K10808	ribonucleoside-diphosphate reductase subunit M2	1.17.4.1
AS4033	A	K10703	3-hydroxy acyl-CoA dehydratase	4.2.1.-
AS1527	A	K01620	threonine aldolase	4.1.2.5
AS1447	A	K03029	26S proteasome regulatory subunit N10	NONE
AS9261	A	K01074	palmitoyl-protein thioesterase	3.1.2.22
AS6692	A	K12189	ESCRT-II complex subunit VPS25	NONE
