# Supplementary material for: Genomic Analysis of the Hydrocarbon-Producing, Cellulolytic, Endophytic Fungus Ascocoryne sarcoides
Source: PLoS Genet. 2012 Mar 1;8(3):e1002558. doi: 10.1371/journal.pgen.1002558 (PMC3291568; doi:10.1371/journal.pgen.1002558)
Supplement: Table S14 — Expression values (log2 RPKM Quantile Normalized) for the potential fabG genes (IP011284). For each Gene ID, expression levels are listed for each culture condition. Genes correlated with C8 production are highlighted in yellow. (PDF) [file pgen.1002558.s028.pdf]

Gene Id	CB	PD4	PD14	AMM	CELL	OAC	PD9
AS5565	1.229	0.389	10.429	0.098	0.389	0.135	0.16
AS1593	0.175	0.603	5.371	0.051	0.166	0.064	0.073
AS4820	1.344	0.72	4.14	1.321	0.649	1.147	0.708
AS20601	0.938	1.82	2.758	1.579	1.174	1.73	0.339
AS09422	1.34	1.365	1.331	2.183	1.578	2.179	1.589
AS03598	2.531	1.65	0.603	0.885	0.496	0.786	0.837
AS00836	2.776	4.091	0.122	3.765	1.588	3.493	4.184
AS10725	0.134	0.455	0.097	0.32	0.245	0.202	0.118
AS03457	0.055	0	0.041	0	0.044	0	0
AS05895	0.521	0.062	0.04	0.662	1.385	0.453	0
