# Supplementary material for: Genomic Analysis of the Hydrocarbon-Producing, Cellulolytic, Endophytic Fungus Ascocoryne sarcoides
Source: PLoS Genet. 2012 Mar 1;8(3):e1002558. doi: 10.1371/journal.pgen.1002558 (PMC3291568; doi:10.1371/journal.pgen.1002558)
Supplement: Table S15 — Summary Statistics of Differential Gene Expression. The number of genes expressed (quantile normalized log2RPKM) in each culture condition (Illumina RNA-seq) relative to expression in PD9 (454 long reads). Gene counts are given for six RPKM fold thresholds from 2 to −2. (PDF) [file pgen.1002558.s029.pdf]

QN $\log_2(X/PD9)$	> 2	> 1.5	>1	< -1	< -1.5	< -2	No Change (0)
**CB**	72	190	450	325	86	28	563
**PD4**	19	53	157	78	16	3	602
**PD14**	183	369	697	652	241	90	546
**AMM**	4	16	98	64	17	2	737
**CELL**	20	49	135	65	16	4	579
**OAC**	3	13	94	69	16	2	673
