# Supplementary material for: Genomic Analysis of the Hydrocarbon-Producing, Cellulolytic, Endophytic Fungus Ascocoryne sarcoides
Source: PLoS Genet. 2012 Mar 1;8(3):e1002558. doi: 10.1371/journal.pgen.1002558 (PMC3291568; doi:10.1371/journal.pgen.1002558)
Supplement: Text S1 — Supporting information including additional methods used for and results from the Expanded Association Analysis, Genome Annotation, Models and Clusters, Comparative Genomics, and Transcriptome. (DOCX) [file pgen.1002558.s030.docx]

Text S1

Gianoulis, Griffin and Spakowicz, *et al*

**1 Expanded Association Analysis 3**

1.1 Compound Gene Co-expression Scoring . . . . . . . . . . . . . . . . . . . . . . . . . . . . . 3

1.2 Compound Consistency and Co-occurrence . . . . . . . . . . . . . . . . . . . . . . . . 4

1.3 Retrosynthesis to Infer Pathway Trajectories . . . . . . . . . . . . . . . . . . . . . . . . 4

1.4 Clustered Gene Co-expression . . . . . . . . . . . . .. . . . . . . . . . . . . . . . . . . . . . . 5

**2 Genome Annotation 5**

2.1 RNASilencing . . . . . . . . . . . . . . . . . . . . . . . . . . . . . . . . . . . . . . . . . . . . . . . . 5

2.2 Transposable Elements . . . . . . . . . . . . . . . . . . . . . . . . . . . . . . . . . . . . . . 5

**3 Models and Clusters 5**

3.1 Identifying Gene Clusters and CAZY annotated Distribution . . . . . . . . . . . . . . 5

3.2 Polyketide Synthase (PKS) Predictions . . . . . . . . . . . . . . . . . . . . . . . . . . . . . 6

**4 Comparative Genomics 6**

4.1 Comparison with Plant Genomes . . . . . . . . . . . . . . . . . . . . . . . . . . . . . . . . . . 6

4.2 Comparison with Other Sequenced Fungi . . . . . . . . . . . . . . . . . . . . . . . . . . . . . 7

4.3 Syntenic Analysis . . . . . . . . . . . . . . . . . . . . . . . . . . . . . . . . . . . . . . . . . . . 7

**5 Transcriptome 7**

5.1 General and Mapping Statistics . . . . . . . . . . . . . . . . . . . . . . . . . . . . . . . . . . 7

5.2 Differential Gene Expression . . . . . . . . . . . . . . . . . . . . . . . . . . . . . . . . . . . . . . 7

5.3 TAR Building and Sensitivity Analysis . . . . . . . . . . . . . . . . . . . . . . . . . . . . . 7

5.3.1 Assessing Gene Structure Prediction and Sensitivity Analysis . . . 7

5.3.2 Novel TARs . . . . . . . . . . . . . . . . . . . . . . . . . . . . . . . . . . . . . . . . . . . 8

**6 References 8**

List of Supplemental Figures

1 All vs All Comparison of Illumina Runs

2 Technical Replicates

3 TAR Sensitivity Analysis

4 Example Tracks for TARs

5 Illustrative Example

6 Inferring Product/Reactant Pairs through Compound Profile Consistency

7 Clustering Compound Co-occurrence

8 Example of Inferred Trajectory

9 ClusteredGeneCo-expression001

10 ClusteredGeneCo-expression101

11 ClusteredGeneCo-expression111

12 Fraction of Annotation Covered following Subsampling

13 Comparison of Metabolism of Close Relatives

14 Synteny Analysis

List of Supplemental Tables

1 Volatile Compounds detected

2 Growth Conditions for GC/MS Profiling and RNA Harvesting

3 Transcriptome Mapping Statistics

4 Comparison with CAZY Matches in Close Fungi

5 Plant Genome Comparison

6 Targeted PKS Search for KS and AT domains

7 Targeted PKS Search for ER/DH/KR domains

8 Gene Subset Co-expressed with 001 Compounds

9 Gene Subset Co-expressed with 010 Compounds

10 Gene Subset Co-expressed with 100 Compounds

11 Gene Subset Co-expressed with 101 Compounds

12 Gene Subset Co-expressed with 110 Compounds

13 Gene Subset Co-expressed with 111 Compounds

14 FabG Expression

15 Summary Statistics of Differential Gene Expression

1 Expanded Association Analysis

1.1 Compound Gene Co-expression Scoring

Three of the six conditions profiled by GC/MS resulted in detection of compounds of particular interest. For simplicity, we refer broadly to these three conditions, CB, PD4, and PD14, as the P set for “producing.” The remaining three conditions, CELL, OAC, and AMM, are referred to as the N set for “not producing”. One transcription profile (PD9) was not sampled via GC/MS and is thus excluded from this analysis. It is important to emphasize that volatile products were detected in the N set; however, as we chose to focus on those compounds of potential relevance as biofuels, the synthesis of the N set compounds was not explored further in this analysis. In order to examine the “co-expression” of compounds and a gene, we represented each compound by a six-element vector. The first three elements are binary values for the absence/presence of the particular compound in each condition of the P-set as described in the *Results*. The remaining three elements indicate the lack of compound production in the N-set. Therefore, any single product can be assigned one of seven possible vectors describing its production in the sequenced condition: 001000, 010000, 011000, 100000, 101000, 110000, and 111000. Since both up and down regulation of genes may contribute to compound production, the “1” element in our analysis is not assumed to indicate solely increased gene expression. By including the N conditions symmetric profiles of the first three elements were deconvoluted. For example, a gene expression profile that correlates with a compound profile of 001 could also be correlated with its inverse production profile (110); however, since there are no 110111 compound profiles (because of the N conditions all profiles are ---000), the directionality of the expression can be assigned.

This directionality is best illustrated in Figure S5. In panel A, the green and purple lines (circles and squares, respectively) represent two possible compound profiles (001 and 110, respectively). The black line (diamonds) represents the expression of a gene across these same conditions. *A priori* it is not possible to tell if this gene is correlated with 001 or 110. It can either be positively correlated with 001 or negatively correlated with 110. In B, we include the gene expression from one null state, which disambiguates the correlation. Now the purple line represents the 1100 compound profile and the green line represents 0010 (note, as described above, the actual compound production and gene expression analysis included three null states). Upon addition of the null state data, the gene (black line) now correlates best with the profile represented by the green line (0010). The gene is positively correlated with the 0010 profile because the gene is active when the compounds are produced. In contrast, a gene correlated with the 1100 state (purple line) would be “ON” or “OFF” in the first two conditions when compounds are produced, and the opposite in conditions 3 and 4 where there is no production.

For each of the 7 possible compound profiles described above, we computed the correlation between the expression of each gene in quantile normalized log_2_ RPKM. Each gene was then assigned to the compound profile with which it correlated. To test the significance of this correlation we shuffled the gene expression and its corresponding compound profile 10,000 times to give a random dataset correlation value and compared the two values using a t-test. Only those genes with *p* < .01 were considered significantly co-expressed with the particular compound profile (see Tables S8-S13 for a subset of genes for each profile with their associated KEGG orthologs [1]. No compounds of interest in this analysis had a 011 profile). As mentioned in the text, this analysis is only able to determine if correlation exists between gene expression and a compound profile, not for a specific compound. In order to further parse the results to compound-specific co-expression, we incorporated two additional data points as described below.

1.2 Compound Consistency and Co-occurrence

Compounds were grouped into possible reactant/product pairs by reasoning that the precursor of a product would be consistently observed in all conditions where the product was observed. This is illustrated with a color-mixing example (see Figure S6). Production profiles in which the compound is observed in only a single condition are shaded in the primary additive colors. Products observed in the first condition only (profile 100, colored blue) and products that occur in the second condition only (profile 010, colored red) can only share a common precursor compound or gene if the precursor has a 110 condition profile, colored purple. In this scheme, it is not possible to derive a green product (001) from a purple (110) precursor. We denoted any of these precursor-product pairings as “inconsistent” and did not use them in any further analysis. However, it is recognized that a number of circumstances would yield a false “inconsistent” result. For example, a precursor product may not be not observed because it never accumulates to detectable levels, resulting in a purple (110) profile when in actuality it follows the black (111) profile.

To address these false negatives, we examined the co-occurrence of compounds in a meta-analysis of the *Ascocoryne* genus [2]. We hypothesized that the variation in enzyme expression and kinetics between genus members would result in different unobservable intermediates. By examining the compound consistency across a broader range of conditions and species, we were able to infer some of these “unobservable” intermediates. We represented each compound as binary vector in this compound co-occurrence meta-analysis as described above. A label was also assigned to each compound vector on the basis of the compound’s P-set profile color determined from the present *A. sarcoides* data. If compound co-occurrence is a general feature, we expect that compounds with similar colors will cluster more closely than those of different colors. The Lance & Williams Bray-Curtis nonmetric coefficient (DLW) was used to compute the distance between each compound.

DLW = (L − BI)/(Bi + Bj) *(1),*

where L is the length of the bit string, or vector, BI is the total number of times two vectors agree, Bi is the total number of “1” or ON bits in compound i and corresponding Bj is the number of ON bits in compound j. The DLW metric is effectively a weighted XNOR logic gate, where more weight is given to matching ON states then matching OFF states. For example we can compare two profiles; compound A with a profile 0001 and compound B with also with profile 0001. Using a classic distance metric, such as Euclidean, these two profiles would be considered as closely related as two 1011 vectors. However, a DLW metric will give more weight to a matching observable (“1”) than to matching unobservables (“0”) and the matching 1011 vectors will now be considered more closely related.

Using the DLW metric, we clustered the compound list across the different species and conditions. This approach is particularly useful because there is more GC/MS data available than is coupled with RNA sequencing profiles. We first color-coded the compounds from the conditions with available RNA-seq data based on their production profiles, then added the compounds only detected in the previous analysis of production across the *Ascocoryne* genus (colored in Brown) [2]. Through this scheme we were able to infer how consistently two compounds co-occurred. As an example, when we cluster our high interest compounds by this method, similar product profiles do indeed group together (Figure S7). In particular, the green compounds (001) and the majority of purple compounds (110) cluster together. The tight compound co-occurrence observed by this clustering demonstrates the efficacy of our “compound consistency” method for inferring metabolic pathways.

1.3 Retrosynthesis to Infer Pathway Trajectories

To further inform the pathway trajectories inferred from the product-precursor pairs made in the “Compound Consistency and Co-occurrence” analysis, we used knowledge of known chemical reactions. Although for many particular reactions specific enzymes have yet to be discovered, known chemical functions or activities can be used to infer additional precursor/product pairings. As an example, in Figure S8,A, we propose a hypothetical schema for the production of octane based on our compound co-occurrence analysis. This proposed pathway is supported by results in the meta-analysis of the *Ascocoryne* genus production, where octane production was coupled with the intermediates seen in Figure S8,A in seven of nine instances. As discussed above, we hypothesize that differences in the enzyme expression and kinetics between members and conditions may result in the variation of unobserved intermediates.

Figure S8,B illustrates possible side reactions that may occur in the cell and that would likely need to be engineered during optimization of alkane production to remove unwanted alkane derivatives. The meta-analysis of *A. sarcoides* and others from the *Ascocoryne* genus, suggests that ester formation diverts the primary alcohol compounds (e.g., 1-heptanol). from the pathway that forms the dehydrated alkenes (e.g., heptene) . Griffin *et al* 2010 reported production of the propyl, hexyl, octyl, and nonyl acetate esters, suggesting that increasing production of all chain length alkanes may require the down regulation of the enzyme that converts primary alcohols to esters [2].

1.4 Clustered Gene Co-expression

For each compound profile, the set of genes was further partitioned using a k-means clustering to identify co-expressed genes. This co-expression can be used to assign functions for genes that do not have known homologs or annotations. Figures S9-11 illustrate the clustering obtained for three of the seven compound profiles. The genes for the 001 profile partition most simply, where two clusters were identified that represent the up and down regulated states (Figure S9). However, other profiles, such as 101 encompass genes that partition into six clusters based on their expression patterns (Figure S10).

2 Genome Annotation

2.1 RNA Silencing

Given that *A. sarcoides* is a non-model organism, we also searched for auxotrophic markers and RNA silencing machinery, which are important tools for the genetic manipulation of organisms. Putative identification of both argonaute (IPR021103) and dicer proteins was made through alignment with known fungal genes [3] . In addition, the genes used for the following auxotrophic markers were identified: ADE2, CAN1, LEU2, LYS2, MET15, and URA3.

2.2 Transposable Elements

We identified 16 families of transposable elements using the PALS pipeline [4].

3 Models and Clusters

3.1 Identifying Gene Clusters and CAZY annotated Distribution

In fungal genomes, many biosynthetic genes involved in the production, export and regulation of a compound exhibit genomic co-localization resulting in higher than average gene density within the co-localized region (reviewed in [5]). This feature was used to identify potential biosynthetic gene clusters within the *A. sarcoides* genome. First, the number of base pairs of exons, introns and intergenic regions were counted for 10 kb segments taking 1 kb steps. The gene density was calculated as the total number of exon base pairs within a given 10 kb segment. To determine if a given gene density was higher than average, a null model was generated by randomly shuffling the placement of the genes, introns and intergenic regions 10,000 times and calculating the resulting gene density as before. From this distribution, a *p*-value was calculated representing the gene density in the real scaffolds. If *p* < .05 for adjacent windows, we merged them. In this manner, we identified 77 gene clusters, many of which contain known secondary metabolite genes. It is interesting to note that often MFS (major facilitator superfamily, IPR011701) and other transporters co-localized with these metabolic enzymes. We also noted many cellulases and other CAZY annotated genes within the gene clusters [6]. To examine the distribution of these particular genes within *A. sarcoides*, we implemented a modified version of the above gene density computation as follows.

(1) Identified all the genes associated with CAZY entries and looked at their placement along the scaffolds. All CAZY matches (C=c_1,_,...,c_n_) were sorted based on their scaffold and placement.

(2) Computed the distance between all adjacent pairs (c_1_,c_2_), (c_2_,c_3_), ...,c(_n-1_,c_n)_. If the gene occurred at the end of a scaffold, this distance was set to 0.

(3) Generated a null distribution by extracting a random set of genes, with the total number of genes equal to that of the CAZY match subset (253 genes).

(4) Computed the distances between random set as above.

(5) Repeated steps 3 and 4, 10,000 times.

(6) Used the null distribution to compute a *p*-value for each ”real” match pair distance.

(7) Merged adjacent pairs sharing a *p*-value < .05.

(8) After visual inspection of distances, extended some clusters to include an additional

CAZY match, where the cluster size < 50 kb.

We found that the CAZY matches exhibit a non-random distribution within the genome, as has been previously reported for other fungal genomes [7].

3.2 Polyketide Synthase (PKS) Predictions

Polyketide synthases are known to produce a wide array of secondary metabolites. To more sensitively search the genome for these elements, we performed the following procedure to build fungal specific Hidden Markov Models (HMM) for the β-ketosynthase (KS) and acyltransferase (AT) domains.

(1) Built an anchored alignment around the 13 conserved residues in 1MLA using the eukaryotic PKS identified by Gaffoor *et al* and iteratively realigned following manual curation [8][9].

(2) Discarded those potential PKSs missing the known AT catalytic residue S92.

(3) Split alignment into separate KS & AT domains and realigned.

(4) Used HMMer to build fungal-specific HMMs [10].

We validated the HMM by first applying it to a set of known PKSs not used in building the model [11]. We identified 13 KS domains and 14 AT domains (Table S6). A similar approach was used to build models for dehydratase (DH) and enoyl reductase (ER) domains resulting in 22 genes. Interestingly, genes with KR or ER domains were often single domain proteins, but a search 3 kb upstream and downstream of these genes showed co-localization of genes with acyl carrier protein (ACP), KS, and AT domains, as well as transporters and oxidoreductases (Table S7).

4 Comparative Genomics

4.1 Comparison with Plant Genomes

Endophytes are found within the interstitial tissues of living plants and are known to share DNA through horizontal gene transfer events [12]. When we compared the known plant genomes with that of *A. sarcoides* we identified five PKS clusters involved in the production of plant secondary metabolites, including phenylpropanoid biosynthesis, chalcone synthase, two separate clusters for patatin, and cinnamoyl CoA. We also identified 136 genes with known plant orthologs. Of this subset, eight genes were plant-lineage specific and have not previously been identified outside of plants. Three genes are predominantly found in plants but had known fungal orthologs (see Table S5). Of particular note are the plant-only orthologs with known enzymatic activities, such as alcohol dehydrogenase and alpha hydroxy acid oxidase, magnesium and cobalt transporters.

4.2 Comparison with Other Sequenced Fungi

In order to compare our analysis with the metabolism of other fungi, we mapped *A. sarcoides*, *Scerlotinia sclerotiorum*, *Gibberella zeae* , and *Saccharomyces cerevisiae* to their respective KEGG orthologs (Figure S13) [13]. In comparison, we can see that *A. sarcoides* has more secondary metabolite biosynthesis than *S. cerevisiae*, and there appear to be subtle differences in lipid biosynthesis. Figures for comparison were generated via iPath [14].

4.3 Syntenic Analysis

OrthoCluster, using standard parameters, was run to compute the synteny between *A. sarcoides* and four of its closest sequenced relatives: *Aspergillus niger*, *Aspergillus nidulans*, *Gibberella zeae*, and *Sclerotinia sclerotiorum* [15]. The total number of orthologs and syntenic blocks for each fungus is listed in Figure S14.

5 Transcriptome

5.1 General and Mapping Statistics

Table S3 shows the total number of reads that mapped to gene models or directly to genome. This resulted in a total of 92,530,490 reads mapping to gene models and 132,820,579 mapping to the genome. Mapping of Illumina and 454 long reads and calculations of RPKM values were performed as described in the Materials & Methods, *RNA-seq Analysis.*

5.2 Differential Gene Expression

A table summarizing the changes in gene expression was generated through computing the log_2_ (X/PD9) on the quantile normalized RPKM scores, where X = condition of interest (Table S15) and counting the total number of genes that had 1, 1.5, and 2-fold difference, respectively.

5.3 TAR Building and Sensitivity Analysis

5.3.1 Assessing Gene Structure Prediction and Sensitivity Analysis

As explained in the Materials & Methods, we built a database of transcriptionally active regions (TARs) and performed a sensitivity analysis (Figure S3). We found the most influential parameter in this analysis to be the threshold value, as measured by the number of exon base pairs covered by TARs. The threshold, or minimum number of required reads, was varied from one to four reads and each increase in threshold value resulted in approximately a four percent decrease in the coverage metric. In contrast, an incremental adjustment to either the minRun or maxGap parameters only altered the observed exon coverage by TARs by approximately a tenth of a percent. The threshold of two reads, maximum gap of three base pairs, and a minimum run of 35 base pairs was selected as the TAR parameter set for further studies of the singly mapped RNA-seq reads (Figure S3). The threshold value was selected to be sufficiently permissive for the discovery of many novel RNA transcripts, but not so low as to include every single transcript mapped beyond the gene boundaries.

5.3.2 Novel TARs

As described in the text, the set of TARs not overlapping with exons and at least one kb from any open reading frame were theorized to belong to one of three possible groups: (1) missed novel gene call, (2) untranslated region, and (3) unknown (Figure S4). Among the identified TARs, more than ten percent associated with regions of the genome outside of the annotated genes. Most of these TARs can likely be attributed to untranslated regions (UTRs) flanking active genes. If we exclude all of the TARs within 1 kb of a called gene, 602 TARs remain unaccounted for, approximately 2.4% of TARs.

6 References

1. Moriya Y, Shigemizu D, Hattori M, Tokimatsu T, Kotera M, et al. (2010) PathPred: an enzyme-catalyzed metabolic pathway prediction server. Nucleic Acids Research. Available: http://nar.oxfordjournals.org/content/early/2010/04/30/nar.gkq318.abstract. Accessed 7 Apr 2011.

2. Griffin MA, Spakowicz DJ, Gianoulis TA, Strobel SA (2010) Volatile organic compound production by organisms in the Ascocoryne genus and a reevaluation of myco-diesel production by NRRL 50072. Microbiology: mic.0.041327-0. doi:10.1099/mic.0.041327-0

3. Hammond TM, Bok JW, Andrewski MD, Reyes-Domínguez Y, Scazzocchio C, et al. (2008) RNA Silencing Gene Truncation in the Filamentous Fungus Aspergillus nidulans. Eukaryotic Cell 7: 339 -349. doi:10.1128/EC.00355-07

4. Edgar RC, Myers EW (2005) PILER: identification and classification of genomic repeats. Bioinformatics 21: i152-i158. doi:10.1093/bioinformatics/bti1003

5. Keller NP, Turner G, Bennett JW (2005) Fungal secondary metabolism - from biochemistry to genomics. Nat. Rev. Microbiol. 3: 937-947. doi:10.1038/nrmicro1286

6. Cantarel BL, Coutinho PM, Rancurel C, Bernard T, Lombard V, et al. (2009) The Carbohydrate-Active EnZymes database (CAZy): an expert resource for Glycogenomics. Nucleic Acids Res 37: D233-238. doi:10.1093/nar/gkn663

7. Martinez D, Berka RM, Henrissat B, Saloheimo M, Arvas M, et al. (2008) Genome sequencing and analysis of the biomass-degrading fungus Trichoderma reesei (syn. Hypocrea jecorina). Nature biotechnology 26: 553–560.

8. Yadav G, Gokhale RS, Mohanty D (2003) SEARCHPKS: a program for detection and analysis of polyketide synthase domains. Nucleic Acids Research 31: 3654 -3658. doi:10.1093/nar/gkg607

9. Gaffoor I, Brown DW, Plattner R, Proctor RH, Qi W, et al. (2005) Functional Analysis of the Polyketide Synthase Genes in the Filamentous Fungus Gibberella zeae (Anamorph Fusarium graminearum). Eukaryotic Cell 4: 1926 -1933. doi:10.1128/EC.4.11.1926-1933.2005

10. Eddy SR (2009) A new generation of homology search tools based on probabilistic inference. Genome Inform 23: 205-211.

11. Castoe TA, Stephens T, Noonan BP, Calestani C (2007) A novel group of type I polyketide synthases (PKS) in animals and the complex phylogenomics of PKSs. Gene 392: 47-58. doi:10.1016/j.gene.2006.11.005

12. Richards TA, Soanes DM, Foster PG, Leonard G, Thornton CR, et al. (2009) Phylogenomic Analysis Demonstrates a Pattern of Rare and Ancient Horizontal Gene Transfer between Plants and Fungi. The Plant Cell Online 21: 1897 -1911. doi:10.1105/tpc.109.065805

13. Kanehisa M, Araki M, Goto S, Hattori M, Hirakawa M, et al. (2008) KEGG for linking genomes to life and the environment. Nucleic Acids Res 36: D480-484. doi:10.1093/nar/gkm882

14. Letunic I, Yamada T, Kanehisa M, Bork P (2008) iPath: interactive exploration of biochemical pathways and networks. Trends Biochem. Sci. 33: 101-103. doi:10.1016/j.tibs.2008.01.001

15. Ng M-P, Vergara IA, Frech C, Chen Q, Zeng X, et al. (2009) OrthoClusterDB: an online platform for synteny blocks. BMC Bioinformatics 10: 192. doi:10.1186/1471-2105-10-192
